# Algorithmic targeting in the Iranian–Israeli confrontation: Technical realities, legal thresholds, and the boundaries of human control

**DOI:** 10.12688/f1000research.169794.2

**Published:** 2026-05-16

**Authors:** Lamia faris

**Affiliations:** 1 College of Dentistry, Basic Science, University of Anbar, Ramadi, Al Anbar Governorate, Iraq

**Keywords:** Artificial Intelligence, Military Targeting, Autonomous Weapons, Israeli Defense Forces, Iranian Military Technology, International Humanitarian Law

## Abstract

**Background:**

The integration of artificial intelligence (AI) algorithms in military targeting systems represents one of the most significant technological shifts in modern warfare, fundamentally altering the speed, precision, and scale of military operations. In the context of the Iranian-Israeli conflict, both nations have emerged as early adopters of AI-enhanced military technologies, albeit through markedly different strategic approaches and technological implementations.

**Method:**

This study employs a mixed-method analytical approach that combines technical system assessment, comparative case study analysis, and evaluation of the international legal framework. Data collection utilized cross-checking methodology across multiple source types, including investigative journalism, open-source intelligence (OSINT) platforms, military data, and peer-reviewed studies to address three core research questions regarding technical architecture differences, operational effectiveness metrics, and compliance with International Humanitarian Law.

**Results:**

The analysis reveals substantial differences in both approach and capability. Israeli systems exhibit advanced data integration and surveillance architectures, emphasizing precision and human machine collaboration through platforms such as the Iron Dome, Lavender, and various targeting support technologies. In contrast, Iranian systems prioritize cost-effectiveness and asymmetric capabilities, focusing on autonomous drone operations and cyber warfare integration, albeit with limited independently verified performance metrics. Notable divergences exist in operational doctrine, human oversight mechanisms, and legal compliance frameworks. While field results reveal that the use of artificial intelligence technologies in warfare must be subject to international law to avoid significant civilian casualties.

**Conclusion:**

While both countries have incorporated AI technologies into military operations, critical gaps in international governance underscore the urgent need for robust regulatory mechanisms to oversee autonomous weapon systems. These findings contribute to academic understanding of military AI implementation patterns and inform policy debates on the ethical and legal dimensions of algorithmic warfare

## Key Terminology Definitions

To be consistent throughout this manuscript, the following definitions are taken and upheld:

**Autonomous Weapons Systems (AWS)**: Weapon systems that on activation are able to choose and attack targets without any additional human intervention. This term is also defined as Lethal Autonomous Weapons Systems (LAWS) when discussing the issue of international law.

**AI-supported targeting systems** are military systems that integrate artificial intelligence algorithms to enhance the process of identifying, prioritizing, and engaging targets, with obtaining human authorization such as Lavender and Besorah.

**Defensive Autonomous Weapons Systems**: Autonomous systems developed with defensive functions, including the interception of missiles (e.g., Iron Dome), where the high-tempo threat environment requires automated responses.

**Meaningful Human Control (MHC)**: A criterion that human beings should make knowledgeable and deliberate choices on the lethal force to be employed, and have adequate time and data to make a true judgment. This definition is consistent with ICRC Guidelines NO. 36

**Human-in-the-Loop (HITL)**: Systems that require human operators to authorize each engagement decision before execution.

## 1. Introduction

The integration of artificial intelligence in military targeting systems represents one of the most significant technological shifts in modern warfare, fundamentally altering the speed, precision, and scale of military operations ( Gul et al., 2020). In the context of the Iranian–Israeli conflict, both nations have emerged as early adopters of AI-enhanced military technologies, albeit through markedly different strategic approaches and technological implementations. The Israeli Defense Forces (IDF) have developed sophisticated AI platforms capable of processing vast datasets to identify, track, and engage targets with unprecedented accuracy Systems such as Lavender, which reportedly analyzed data on 2 3 million Gaza residents and generated up to 37,000 potential targets, exemplify a paradigm shift toward algorithmic warfare ( Guardian, 2024). In contrast, Iran has pursued an asymmetric strategy, leveraging AI for drone swarm coordination, cyber warfare enhancement, and autonomous weapon systems designed to counter conventional military superiority ( Gilli & Gilli, 2019).

The expansion of AI-enabled military systems raises critical issues related to operational effectiveness, strategic stability, and compliance with international humanitarian law. Recent developments in the military capabilities of both Israel and Iran highlight a fundamental difference in approaches to integrating AI, the implications of which extend beyond regional security to global governance frameworks for autonomous weapons systems ( Davison, 2020).

This technological arms race unfolds in a complex geopolitical landscape where both countries face existential security concerns, resource limitations, and international judgment. The integration of AI into targeting systems raises fundamental issues related to accuracy, responsibility, and compliance with international humanitarian laws, especially those concerning civilian safety and the need for human oversight in deadly decision-making processes.

This paper aims to provide a detailed overview of how AI algorithms can be used in targeting systems within the Iranian-Israeli conflict, examining technical details, operational effectiveness, ethical and human implications, and strategic impacts of such applications. By analyzing existing data on system deployment and operational patterns, the study aims to address critical gaps in our understanding of AI’s role in contemporary warfare and its broader impact on regional and global security.

RQ1: What are the main differences in the technical architecture and operational mechanisms of AI-enhanced targeting systems used in Israeli and Iranian military applications?

RQ2: How do the operational efficiency indicators and strategic impacts of these systems differ, and what are their implications for regional security and stability?

RQ3: To what extent do current military AI implementations adhere to international legal standards, particularly international humanitarian law and established governance frameworks?

### 1.2 Research objectives

The primary objectives of this research are to: (1) provide a systematic comparative analysis of documented AI military systems in Israeli and Iranian defense applications; (2) evaluate the technical, operational, and strategic characteristics of these systems within their respective doctrinal contexts; (3) assess compliance with international legal frameworks governing autonomous weapons; and (4) identify implications for regional security stability and global governance mechanisms.

## 2. Literature review

### 2.1 Analytical techniques

Academic research in the field of military artificial intelligence has undergone a remarkable transformation over the past decade, progressing from largely theoretical debates about the feasibility and ethics of autonomous weapons to empirical investigations of systems already deployed in active conflict environments. This evolution reflects both the accelerating pace of AI development and its increasingly visible role in shaping modern military doctrine. The growing body of scholarship draws from computer science, international law, strategic studies, and ethics, reflecting the inherently interdisciplinary nature of the subject. Foundational contributions to the field have sought to establish conceptual clarity in a domain prone to definitional ambiguity. In this context ( King, 2024), proposes a conceptual framework that distinguishes between the uses of AI in data analysis, decision support, and autonomous execution, noting that the predominant use of AI in modern military contexts is primarily to enhance intelligence analysis capabilities, rather than to automate combat decisions.This view challenges usual stories that focus on fully autonomous killing machines, instead pointing out AI’s main role in boosting intelligence and speeding up targeting. Scharre (2018) in his foundational analysis of autonomous weapons, offers a taxonomy of human-machine interaction models by dividing between directly decision human-in-the-loop and human-out-of-the-loop systems as examples, proving that the extent of the human control lies on a spectrum, and is not a binary categorization. This paradigm becomes crucial in the analysis of Israeli and Iranian systems that are at the opposite sides of this scale. Horowitz (2019) extends this analysis by examining how military AI adoption patterns vary according to national innovation capacity, arguing that states with robust civilian technology sectors—such as Israel—are better positioned to develop sophisticated AI military applications, while resource-constrained states pursue asymmetric approaches.

The theoretical framework for understanding AI military applications, as identified by Joshi (2018) encompasses three primary domains: data processing and analysis, decision support systems, and autonomous execution capabilities. Research indicates that militaries worldwide have prioritized the first two domains, utilizing AI to accelerate and enhance military targeting through improved data fusion and pattern recognition, rather than complete automation of lethal decision-making. The theoretical understanding of the use of artificial intelligence in the military field includes three analytical axes, as pointed by Joshi (2018). Technical efficiency, which provides for algorithm development and system integration; operational doctrine, which addresses human-machine interaction mechanisms and usage strategies; and strategic impact, which focuses on the role of AI in enhancing deterrence and controlling escalation dynamics. This model provides a basic analytical framework for comparing different systems ( King, 2024). Bode and Huelss (2018) which study the problem of autonomous weapons as a challenge to traditional norms of international security governance, holding that the dissemination of such technologies creates a normative uncertainty that is hard to resolve by current legal frameworks. These contributions collectively establish the theoretical foundation upon which this study’s comparative analysis is built.

### 2.2 Case studies in AI military implementation

Empirical analysis of AI deployment in military contexts reveals diverse implementation strategies across different operational environments King’s research examined two significant cases: the British Army’s COVID-19 testing operation in Liverpool (2020) and the U S Security Assistance Group-Ukraine operations (2022), demonstrating how AI systems process multi-source data to optimize targeting and resource allocation ( Emegha & Okoli, 2025). These case studies identified the key principles when it comes to AI usage in the military sphere: the vital role of data quality and integration, the need of human agents to be present in the sphere of strategic decision-making as well as the possibility of the AI systems to greatly increase operational tempo and precision when properly utilized and guided by limits.

The article by Ekelhof (2019) explores the role of autonomous weapons in the modern war by stating that the speed with which AI-based targeting is accomplished radically changes the connection between decision-making and responsibility, ( Sharkey, 2012) notes the challenge of the responsibility gap in lethal autonomous weapons, in which attributing any legal or moral responsibility is difficult when algorithmic systems are involved in the process of making target decisions. These scholarly contributions present the necessary critical background into which the given systems can be assessed within the framework of this research.

### 2.3 Regional AI military development patterns

Recent scholarly analysis of Middle Eastern military AI development reveals distinct national approaches shaped by strategic priorities, technological capabilities, and resource constraints. Research on Israel’s AI military systems indicates a focus on defensive applications and precision targeting, driven by security imperatives and technological advantages ( Journal, 2024). Conversely, Iranian military AI development follows an asymmetric strategy emphasizing cost-effective force multipliers and unconventional capabilities. Studies indicate Iran’s approach prioritizes “rapid deployment of novel technologies, often through unpredictable proxies” as a means of countering conventional military disadvantages ( Rocks, 2021).

### 2.4 Ethical and legal frameworks

The debate about autonomous weapons systems in law and ethics has intensified due to the advancement of artificial intelligence. The International Committee of the Red Cross has articulated specific concerns regarding human control requirements and accountability mechanisms in AI-enabled military systems ( ICRC, 2019). It notes that no law can be delegated to a machine, computer program, or weapons system that would have any legal obligation to comply with international humanitarian law. As reported by the current research, the conflict between the operational utility provided by AI systems and the significant level of human control over lethal decision-making is emphasized. This conflict is especially sharp in high-tempo warfare, when the pace of AI-supported targeting may outpace human ability to monitor and take action. Roff and Moyes (2015) produce the notion of meaningful human control as a principle of regulation and suggest that the critical question is not to make sure that a human remains somewhere in the decision chain, but to make sure that human interference is meaningful to a point where it is considered genuine oversight. This difference is especially applicable on hearing that there are 20-second review times in Israeli targeting systems, which calls into question the notion that nominal human-in-the-loop processes are meaningful control. Recent legal analyses highlight the necessity of effective human control as a fundamental regulatory principle. However, the lack of consensus on practical definitions and implementation standards hinders the realization of this goal ( Roff & Moyes, 2015). This regulatory ambiguity complicates the assessment of system compliance and obstructs efforts to develop appropriate governance frameworks.

### 2.5 Identified research gaps

While growing, the current literature has several deficiencies that this paper seeks to tackle. First, existing comparative studies of AI military systems tend to concentrate on their technical architecture or legal compliance, with little attempted integration of the two dimensions. Furthermore, even though it is one of the most active theatres for the utilization of AI-enabled military action, the Iranian-Israeli context has benefitted from only limited systematic academic attention. Although major investigations and journalistic pieces on systems like ‘LAVENDER’ and ‘BESORAH’ appeared in the media, an academic analysis contextualising them within existing theories is lacking. The relation of claimed system capabilities to observed humanitarian outcomes have not been studied systematically in the academic literature. Through a structured comparative analysis that integrates technical, operational, ethical and legal dimensions, this study seeks to fill this gap.

## 3. Methodology

### 3.1 Research design

This study employs a hybrid qualitative–quantitative analytical framework to investigate the role of artificial intelligence algorithms in military targeting systems within the Iranian–Israeli conflict. The methodology integrates technical system analysis, comparative architecture evaluation, and ethical–legal framework synthesis. More specifically, this study adopts a comparative case study methodology that combines qualitative system analysis with quantitative performance assessment where verifiable data is available. The research design integrates three analytical approaches: (1) technical evaluation of the system based on documented capabilities and architectural specifications; (2) assessment of operational effectiveness using available performance metrics and deployment patterns; and (3) legal compliance analysis with international humanitarian law standards.

### 3.2 Data collection and sources

Information on conflict areas is both extremely biased and imprecise due to restricted access to the field, the prevalence of propaganda, and the absence of comprehensive ground-truth verification. Bias may manifest in source selection when reporting disproportionately reflects one side’s narrative, or when unrecorded events are omitted from casualty statistics. This distortion may also occur intentionally, as governmental and non-governmental entities may release false or misleading information, resulting in the overstatement or understatement of AI system accuracy and effects. To counter such problems, this research employs a cross-checking methodology across numerous source types, including peer-reviewed literature, open-source intelligence (OSINT) platforms, military data, and investigative journalism. Sources supported by verifiable evidence such as photographs, leaked documents, and confirmed eyewitness testimony are given priority. In the event of contradictions between sources, the contested nature of the information is clearly noted, and no definitive judgments are made based on a single source. The data collection process employed various source categories to ensure comprehensive coverage. The principal categories of sources were as follows:

• Academic database: Web of Science, Scopus, JSTOR, Google Scholar.
• OSINT platforms: Bellingcat, Jane’s Defence Intelligence, OSINT Techniques, Global Security, Center for Strategic and International Studies (CSIS), RAND Corporation, Stockholm International Peace Research Institute (SIPRI).
• Authority documents of the government and the army: Israeli Ministry of Defense publications, IDF official statements, Iranian state media (with appropriate verification caveats), U.S. Department of Defense reports.
• Investigative journalism outlets: The Guardian, +972 Magazine, Al Jazeera Investigative Unit, New York Times, Washington Post.
• Legal and humanitarian sources: ICRC publications, UN reports, Human Rights Watch, Amnesty International.

**3.2.1 Language limitations**

English, Arabic, and Hebrew sources were used. English translations or summaries of respectable analytical institutions were incorporated in the sources in the Persian language. This restriction can lead to bias in favor of sources that are available to Western researchers, and this has been admitted in the limitations section.

**3.2.2 Inclusion criteria**

Studies were included in case they contained the following criteria:

Technical accounts: Documents that give technical specifications, architecture or operational parameters of AI-enabled military systems that are detailed enough to be compared with other systems.

Evidentiary support: Sources that are backed by verifiable facts like photographs, leaked documents, eyewitness testimonies confirmed, or official admissions.

Source credibility: Published by an accepted academic institution, reputable media outlet, editorial standards of quality, well-known research organization or official government/military source.

Cross-verification: Data that has been supported by two or more independent sources, unless it is mentioned as being a single-source claim.

**3.2.3 Exclusion criteria**

The exclusion criteria were that sources must:

- Derived from information whose authenticity is misinformed or propagated without corroboration.
- Only speculative or theoretical abilities and not recorded.
- Were not attributable to known persons or schools.
- Had serious internal contradictions that it could not explain.

### 3.3 Analytical framework

Systems were evaluated across six analytical dimensions to enable systematic comparison, as shown in Table A. Systems were evaluated across six analytical dimensions. Each dimension was scored on a 0-100 scale with the following capability categories: Low (0-30), Moderate (31-60), and High (61-100). The scoring criteria for each dimension are defined below:

**Table A.** Scoring criteria for six-dimension framework.

Dimension	Low (0-30)	Moderate (31-60)	High (61-100)
Data Integration	Single or limited data sources; minimal real-time capability	Multiple data sources with partial integration; some real-time processing	Comprehensive multi-modal fusion; full real-time processing across sensor types
Algorithmic Architecture	Rule-based systems; limited ML application	Standard ML algorithms; moderate neural network depth	Advanced deep learning; sophisticated predictive models; multi-task architectures
Human Oversight	Minimal oversight; autonomous execution without authorization requirements	Human-on-the-loop with intervention capability; time-constrained review	Human-in-the-loop with meaningful review time; robust override mechanisms
Deployment Scale	Limited deployment; experimental or pilot programs	Operational deployment in specific contexts; moderate target generation	Large-scale operational deployment; high-volume target generation
Operational Accuracy	Accuracy <70%; high error rates; limited validation	Accuracy 70-85%; moderate validation; some independent verification	Accuracy >85%; robust validation protocols; independent verification available
Ethical/Legal Compliance	Significant IHL concerns; minimal distinction/proportionality mechanisms	Partial compliance mechanisms; some documented concerns	Robust IHL compliance architecture; documented precautionary measures

Systems were evaluated across six analytical dimensions to enable systematic comparison:

1. **Data integration capabilities:** Assessment of multi-source data fusion, sensor integration, and real-time processing capacity.
2. **Algorithmic architecture:** Analysis of machine learning approaches, neural network structures, and decision-making frameworks.
3. **Human oversight mechanisms:** Evaluation of human-machine interaction protocols, authorization requirements, and override capabilities.
4. **Deployment scale:** Documentation of operational deployment extent, target generation capacity, and engagement frequency.
5. **Operational accuracy:** Analysis of reported performance metrics, error rates, and validation methodologies.
6. **Ethical and legal compliance:** Assessment against International Humanitarian Law principles including distinction, proportionality, and precaution.

The process of reconciling the sources will involve the following procedures:

In the case of conflicting information about the capabilities or performance of the system given by the sources, the process of reconciliation was performed as follows:

### 3.4 Priority level

(a) Technologically independent confirmation of the technical documentation;

(b) Government/military records which have been proved true;

(c) Verifiable investigative journalism; names on the sources;

(d) OSINT performance of a developed establishment;

(e) Source reporting anonymously (with sufficient reservations).

Conflict notation: In case of any serious disagreement despite the implementation of the hierarchy, conflicting claims are clearly stated in the text using suitable uncertainty language.

Minimum estimation: In situations where the quantitative estimations were contradictory, the lesser estimation was used unless there was particular evidence of greater estimations.

## 4. Results

### 4.1 AI systems in Israeli military operations

**4.1.1 Lavender system**

The Lavender system represents one of the most advanced applications of AI-driven target identification in modern warfare. Lavender is a machine learning algorithm developed by the elite Unit 8200 in the IDF that uses mass surveillance data of the approximately 2.3 million people of Gaza to produce individual threat scores between 1 and 100, according to the convictions of the Israeli army, based on behavioral patterns, communication networks, and movement patterns ( Shabbir, 2024; Guardian, 2024).

The Lavender system represents one of the most advanced applications of AI-driven target identification in modern warfare. Developed by the IDF’s elite Unit 8200, Lavender employs machine learning algorithms to analyze mass surveillance data of Gaza’s approximately 2.3 million residents, generating individual threat scores ranging from 1 to 100 based on behavioral patterns, communication networks, and movement patterns ( Shabbir, 2024).

**Technical architecture:** Lavender is built on a multi-layered data fusion system that integrates visual surveillance, cellular communications, social media activity, and battlefield intelligence. The system uses supervised learning to identify patterns associated with militant profiles, analyzing features indicative of combatant status such as WhatsApp group memberships, frequency of address changes, and communications with known targets ( Shabbir, 2024). The machine learning model was trained on historical data from confirmed combatants and refined through iterative validation processes.

**Operational capabilities:** During peak operational periods, Lavender generated approximately 37,000 potential human targets. According to IDF sources, the system achieved approximately 90% accuracy in target identification. System outputs feed directly into operational planning, with human operators spending an average of 20 seconds per target verification, primarily to confirm gender rather than to scrutinize targeting rationale or evaluate civilian risk factors.

**Performance metrics:** Unit 8200 internal validation studies suggested that Lavender could reach approximately 90 percent accuracy in detecting valid military targets. This figure has been challenged by external analysts who note that the system relies on statistical correlation rather than verified intelligence. As noted above, the 10% error rate applied to the reported 37,000-target list yields a hypothetical misidentification figure of approximately 3,700 individuals—an extrapolation that must be treated with caution given that the underlying accuracy claim itself remains unverified through independent assessment. Independent validation of the system’s performance remains unavailable due to operational secrecy.

One clear indication of the inaccuracy of AI targeting is the high number of civilian casualties in Gaza. Moreover, the system does not account for whether targets are among civilians or near children at the time of engagement, which presents serious ethical and legal concerns regarding compliance with distinction and proportionality principles in International Humanitarian Law. Information on the internal validation methodology is scanty, and whether operational accuracy was actual as reported has not been established. According to investigative reporting, a high portion of the targeted people are usually hit while at home ( Patel, 2024). An automated search engine named Where’s Daddy? was applied together with Lavender to identify targets in households. Basing their claim on the claim of anonymous Israeli intelligence officers, the IDF often preferred to attack their targets in their homes because operations were custom-made to handle such attacks.

**4.1.2 Besorah: Accelerated target production**

The Besorah system serves as a complementary platform to Lavender, focusing on infrastructure and high-value target identification through automated analysis of drone imagery, intercepted communications, and behavioral pattern recognition. According to technical documentation, Besorah can generate up to 100 prioritized targets daily, utilizing advanced computing and machine learning algorithms to evaluate target significance and strike feasibility ( Gul et al., 2020).

**Algorithmic approach:** Besorah employs a traffic-light classification system (red/yellow/green) to prioritize targets based on military value, civilian risk assessment, and operational feasibility. The system integrates real-time intelligence feeds with historical pattern analysis to predict target behavior and optimal engagement windows. This predictive capability enables proactive targeting while theoretically maintaining consideration of civilian protection requirements.

**Integration with human oversight:** Unlike fully autonomous systems, Besorah maintains human command authority for final targeting decisions. Military commanders review system recommendations and must explicitly authorize strikes. However, compressed decision timelines—often minutes rather than hours—limit the depth of human analysis possible. This temporal constraint raises questions about the meaningfulness of human oversight when system-generated recommendations arrive at rates exceeding thorough human evaluation capacity.

**4.1.3 SMASH family: Precision engagement systems**

The SMASH (Smart Shooter) family represents AI-enhanced fire control systems designed for precision engagement across multiple platforms. These systems combine computer vision, predictive algorithms, and automated fire control to enhance accuracy and reduce collateral damage in complex operational environments ( Soldier Systems Daily, n.d.).

**Technical components:** SMASH systems integrate several AI-driven capabilities:

• **Target acquisition algorithm:** Employs computer vision to identify and classify potential targets in real-time, distinguishing between combatants, civilians, and objects.
• **Tracking algorithm:** Continuously computes target trajectory while accounting for environmental variables including wind, humidity, and target velocity.
• **Lock-and-fire control:** Enables autonomous firing when target engagement probability exceeds predetermined thresholds.

Table 1 presents the technical specifications and key features of the SMASH family components.

**Table 1.** SMASH technical components.

SMASH variant	Platform	Caliber support	Key features
SMASH 2000	Handheld Extension	5.56 mm, 7.62 mm	Individual soldier targeting system
SMASH Hooper	Remote Controlled	5.56 mm, 7.62 mm	Safe-distance operation capability
SMASH Dragon	Heavy Weapons	40 mm Grenades	Vehicle and UAV integration

Source: (Soldier Systems Daily, n.d.).

**4.1.4 Iron Dome: Predictive defense architecture**

The Iron Dome system exemplifies defensive AI application, employing artificial intelligence to predict incoming projectile trajectories and calculate optimal interception parameters. Radar data is analyzed by machine learning programs to distinguish between threatening and benign projectiles, with engagement resources allocated only to genuine threats.

**Algorithmic components:**

Algorithmic components are essential for enhancing threat response capabilities in modern autonomous defense systems:

1. **Trajectory prediction models:** Neural networks calculate projectile paths by integrating ballistic properties with dynamic environmental conditions
2. **Threat assessment algorithms:** AI-powered systems analyze impact probabilities and expected damage severity to prioritize engagement decisions
3. **Interception optimization:** Real-time algorithms determine optimal timing and targeting strategies for interceptor deployment, maximizing accuracy while minimizing collateral risks

**Performance metrics:** The Israeli military reports Iron Dome achieves approximately 90% interception rate for short-range rockets and mortars. The AI interface reportedly reduces time required for threat assessment and engagement by approximately 40% compared to previous systems, enabling faster response to evolving threats. The system’s selective engagement algorithm reduces operational costs by avoiding interception of projectiles predicted to land in uninhabited areas ( Ramachandra, 2023).

**System limitations:** Despite high performance, Iron Dome faces several constraints. Each interceptor missile costs approximately $40,000-$50,000, making sustained use financially burdensome during prolonged or frequent attacks. While highly optimized for short-range threats, the system demonstrates reduced effectiveness against long-range ballistic missiles or complex multi-directional assault patterns. The system can be overwhelmed by high-volume simultaneous rocket launches, potentially reducing interception coverage and effectiveness. The October 2023 Hamas assault and subsequent Iranian-Israeli exchanges demonstrated that Iron Dome’s interception range and effectiveness can be degraded under sustained, high-volume attack conditions ( Al Jazeera, n.d.).

**4.1.5 Specialized systems: IRIS and Goshawk**

Recent advancements in autonomous robotics have led to specialized systems for reconnaissance and counter-drone operations:

**IRIS reconnaissance robot:** Features AI-driven surveillance capabilities in confined spaces through computer vision algorithms that identify personnel, objects, and explosive devices. The system is compact and lightweight (1.85 kg, 20 × 23 × 11 cm dimensions), with 200 m communication range, 5 km/h maximum speed, and 1 kg payload capacity. IRIS enables reconnaissance in urban environments where human entry presents elevated risk.

**Goshawk autonomous interceptor:** Serves as a fully autonomous counter-unmanned aerial vehicle (UAV) system. It includes day and night detection capabilities, non-lethal drone capture methods, and smart nest recovery systems, allowing interception without collateral damage or continuous human involvement. These platforms collectively indicate evolution toward integrated, precision-oriented robotic systems emphasizing scalability and operational adaptability.

### 4.2 AI Deployment in Iranian defense strategy

**4.2.1 Strategic asymmetric approach**

Iran’s military AI development follows a fundamentally different paradigm from Israel’s precision-focused systems, emphasizing asymmetric capabilities designed to counter conventional military superiority through cost-effective force multipliers and unconventional deployment methods. This strategy reflects Iran’s resource constraints, technological limitations, and strategic doctrine of “forward defense” through proxy networks ( Rocks, 2021). Figure 1 illustrates the organizational structure of Iranian cyber and information operations, demonstrating integration of AI capabilities across multiple operational domains.

**Figure 1.**
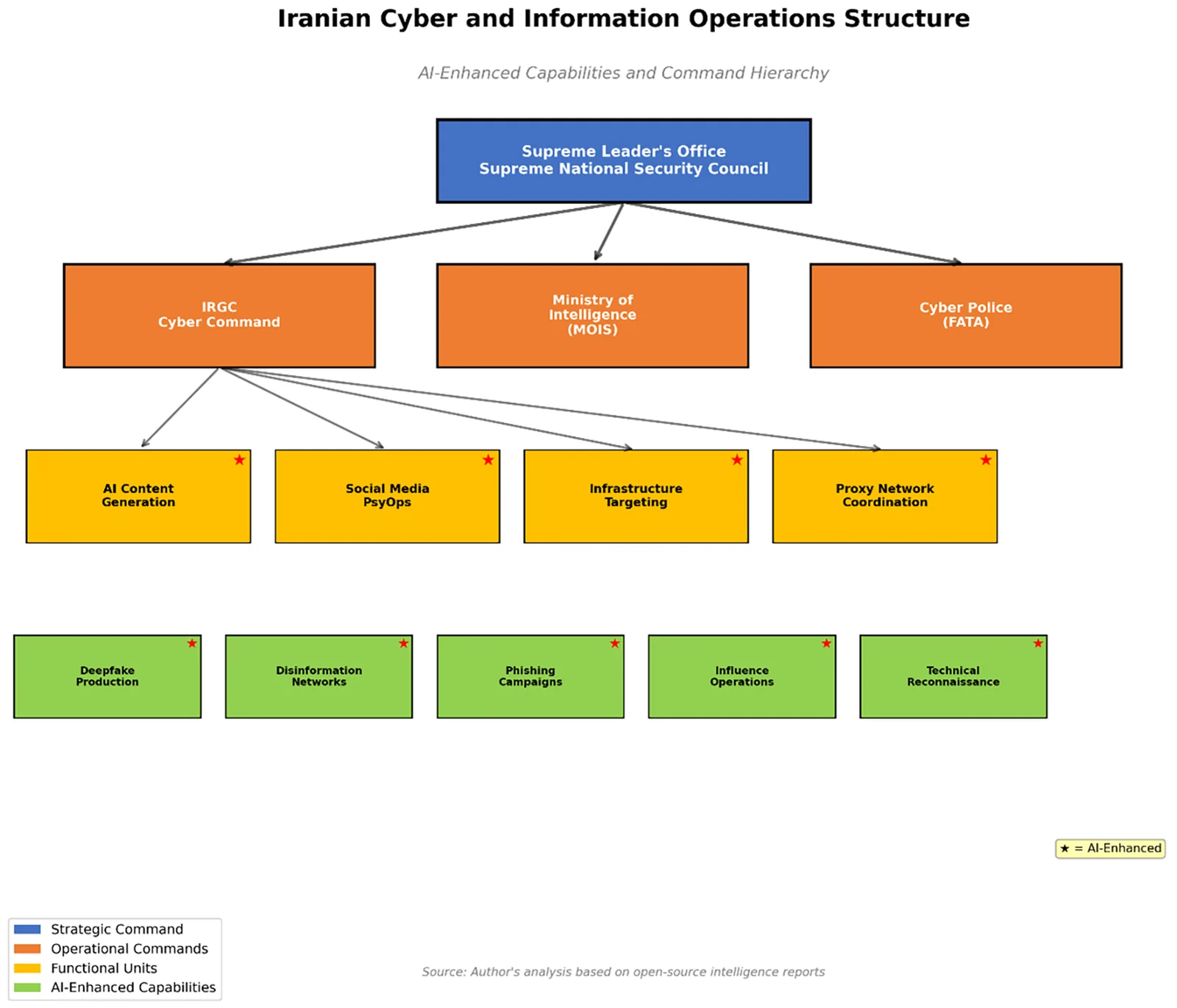
Iranian cyber and information operations structure. This is a conceptual diagram constructed by the author based on organizational analysis from Rocks (2021), Gen et al. (2025), and Khorrami (2024).

Iranian military authorities explicitly describe AI as a force multiplier, analogous to their proxy network approach throughout the Middle East. Consistent with strategic analysis, Iran has mobilized significant numbers of highly educated computer engineers to pursue AI capabilities despite hardware and infrastructure limitations, concentrating on rapid deployment of basic autonomous capabilities rather than complex precision platforms ( Mieses & Noelle Kerr, 2024).

**4.2.2 Autonomous drone systems ( Mieses & Noelle Kerr, 2024)**

**Suicide drone technology:** Iran has deployed suicide drones utilizing artificial intelligence for autonomous target detection and engagement through image processing capabilities and pattern recognition algorithms. The Iranian Army Ground Forces and Islamic Revolutionary Guard Corps (IRGC) have identified these systems as primary components of Iran’s autonomous weapons arsenal.

**Swarm Intelligence:** Iran has developed autonomous drone swarms featuring mother platforms controlling multiple smaller suicide drones. This system represents Iranian advancement in distributed autonomous control and coordination, though capabilities remain incompletely verified through independent technical assessment. The swarm architecture enables coordinated attacks capable of overwhelming air defenses through numerical advantage and distributed attack vectors, complicating defensive responses ( Ramachandra, 2023). Table 2 summarizes the types of autonomous drone systems deployed by Iran, their AI integration levels, and operational status.

**Table 2.** Autonomous Drone systems.

System type	Capability	AI integration	Operational status
Autonomous Suicide Drones	Target Detection & Engagement	Image Processing AI	Operational
Drone Swarms	Coordinated Attack	Swarm Intelligence	Demonstrated
Mobin Cruise Missile	Autonomous Navigation	DSMAC Guidance	Operational
Ground Robots	Armed Reconnaissance	Autonomous Network	Development

Source: Based on Iranian military announcements and open-source intelligence, 2020-2024.

Figure 2 provides a visual representation of Iranian AI drone system capabilities across different operational parameters.

**Figure 2.**
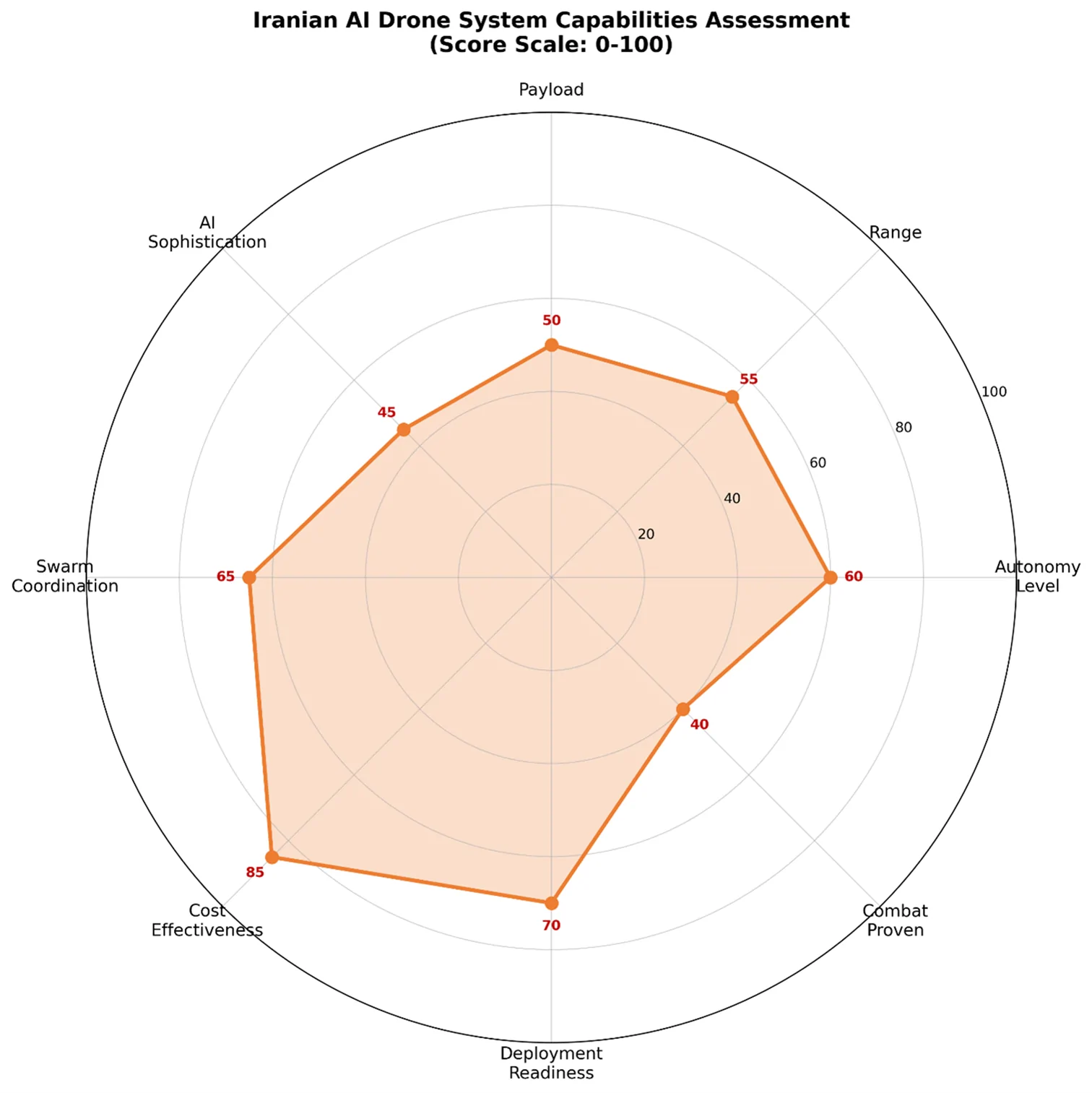
Iranian AI Drone system capabilities.

Comparative assessment of Iranian AI-enabled drone systems across autonomy level, range, payload capacity, AI sophistication, and operational readiness. Source: Author’s analysis based on available technical intelligence.

**4.2.3 AI-Enhanced missile systems**

The Mobin cruise missile system exemplifies Iran’s incorporation of artificial intelligence into long-range precision strike capabilities. This system utilizes Digital Scene Matching Area Correlation (DSMAC) guidance, an AI technology initially developed in the 1980s and subsequently refined for modern applications. This advancement facilitates autonomous navigation to predetermined targets without relying on external guidance systems, providing resilience against electronic warfare and GPS denial ( Rocks, 2021). DSMAC technology enables the missile to compare real-time terrain imagery with stored reference images, making autonomous course corrections to reach designated targets. While representing relatively mature technology, DSMAC integration demonstrates Iran’s capacity to adapt existing AI approaches to overcome technological and resource constraints.

**4.2.4 Cyber warfare integration**

Iran has significantly integrated artificial intelligence into its cyber warfare capabilities, representing one of the most advanced applications of AI in its military arsenal. According to security analysis, Iran’s cyber operations exhibit “increasing sophistication and potentially more dangerous cyber capabilities” through AI integration ( Emegha & Okoli, 2025).

**AI-Enhanced cyber operations:** Iranian cyber capabilities encompass advanced digital techniques designed to influence, deceive, and manipulate information spaces:

• **Deepfake content creation:** Production of highly realistic synthetic media for propaganda and influence operations
• **Sophisticated phishing campaigns:** AI-powered targeting of strategically significant individuals and institutions with personalized social engineering attacks
• **Fake news platforms:** Establishment of AI-curated disinformation networks targeting specific population segments
• **AI-powered personas:** Deployment of autonomous virtual characters conducting psychological operations and shaping public opinion trends on social media platforms ( Gen et al., 2025)
  These capabilities represent significant force multiplication in information warfare, enabling Iran to project influence and conduct operations despite conventional military disadvantages.

### 4.3 Limitations and constraints of Iranian AI systems

Despite technological developments, Iranian military AI capabilities remain constrained by structural and technical factors limiting system sophistication and operational effectiveness:

**Hardware constraints:** International sanctions severely limit Iran’s access to advanced semiconductors and high-performance computing hardware, adversely affecting AI processing sophistication and precision. While advanced military systems employ specialized processors, Iranian systems typically utilize commercial-grade components, resulting in reduced operational efficiency. Sanctions have also resulted in absence of unified monitoring and sensing infrastructure, hindering provision of sufficient and diverse training data for AI applications. This negatively affects efficiency of Iranian AI-supported targeting systems and their adaptability to varied operational contexts ( Khorrami, 2024).

In contrast, Israeli systems benefit from extensive surveillance networks and sophisticated data environments, receiving technical support from leading technology companies, particularly in communications surveillance. These capabilities significantly enable collection of actionable intelligence on specific targets, enhancing effectiveness of AI-driven military operations (CSIS; RAND Corporation, n.d.).

**Integration issues:** Institutional fragmentation within Iranian military organization—divided among Revolutionary Guard, regular army, and proxy forces—generates substantial challenges in AI system harmonization and data exchange across entities. This fragmentation inhibits systems’ capacity to operate in integrated, efficient manner, reducing overall operational effectiveness and limiting strategic coordination capabilities.

### 4.4 Comparative analysis

**4.4.1 Technological sophistication comparison**

Figure 3 presents a comprehensive comparison of technological capabilities between Israeli and Iranian AI military systems across multiple dimensions.

**Figure 3.**
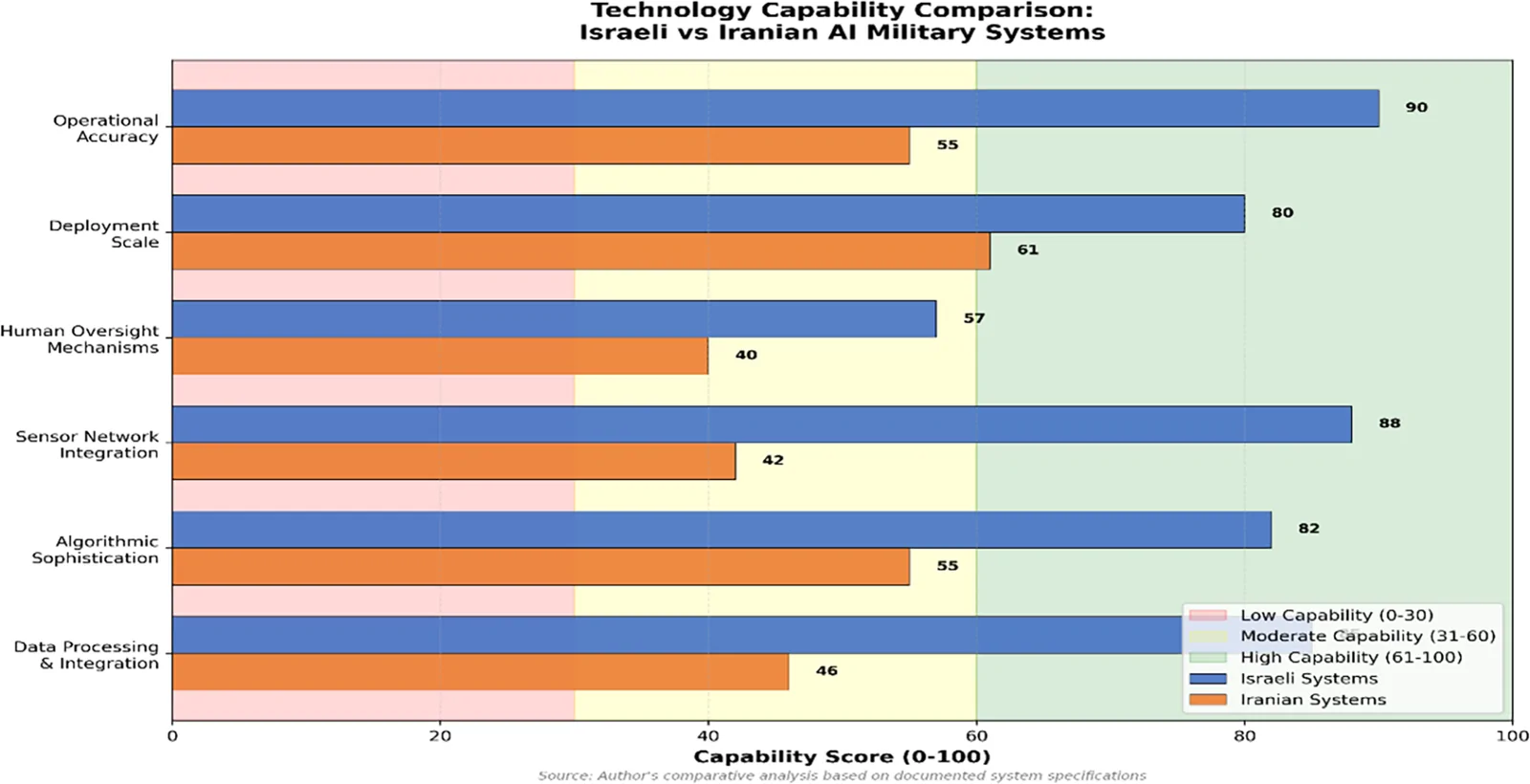
Technology capability comparison (Israel and Iran).

Multi-dimensional comparison illustrating relative technological sophistication between Israeli and Iranian AI military systems across data processing, algorithmic complexity, sensor integration, human oversight, deployment scale, and operational accuracy. Source: Author’s comparative analysis based on documented system specifications.

The comparative analysis reveals substantial disparities in technological sophistication.

**4.4.2 Strategic implementation approaches**

Table 3 outlines fundamental differences between Israeli and Iranian approaches to AI military implementation.

**Table 3.** Technological sophistication comparison.

Israeli approach: Precision and integration	Iranian approach: Asymmetric and distributed
1. Focus on precision, accuracy, and system integration	1. Rapid deployment and cost-effectiveness
2. Emphasis on data fusion and human oversight	2. Asymmetric advantage through swarm capabilities
3. Systematic validation and testing protocols	3. Cyber operations integration
4. Minimizing collateral damage priority (doctrinal)	4. Proxy network utilization
5. High-cost, high-precision systems	5. Lower-cost, moderate-effectiveness systems

Figure 4 compares operational effectiveness metrics between Israeli and Iranian AI targeting systems, highlighting performance differences in accuracy, response time, and deployment scale.

**Figure 4.**
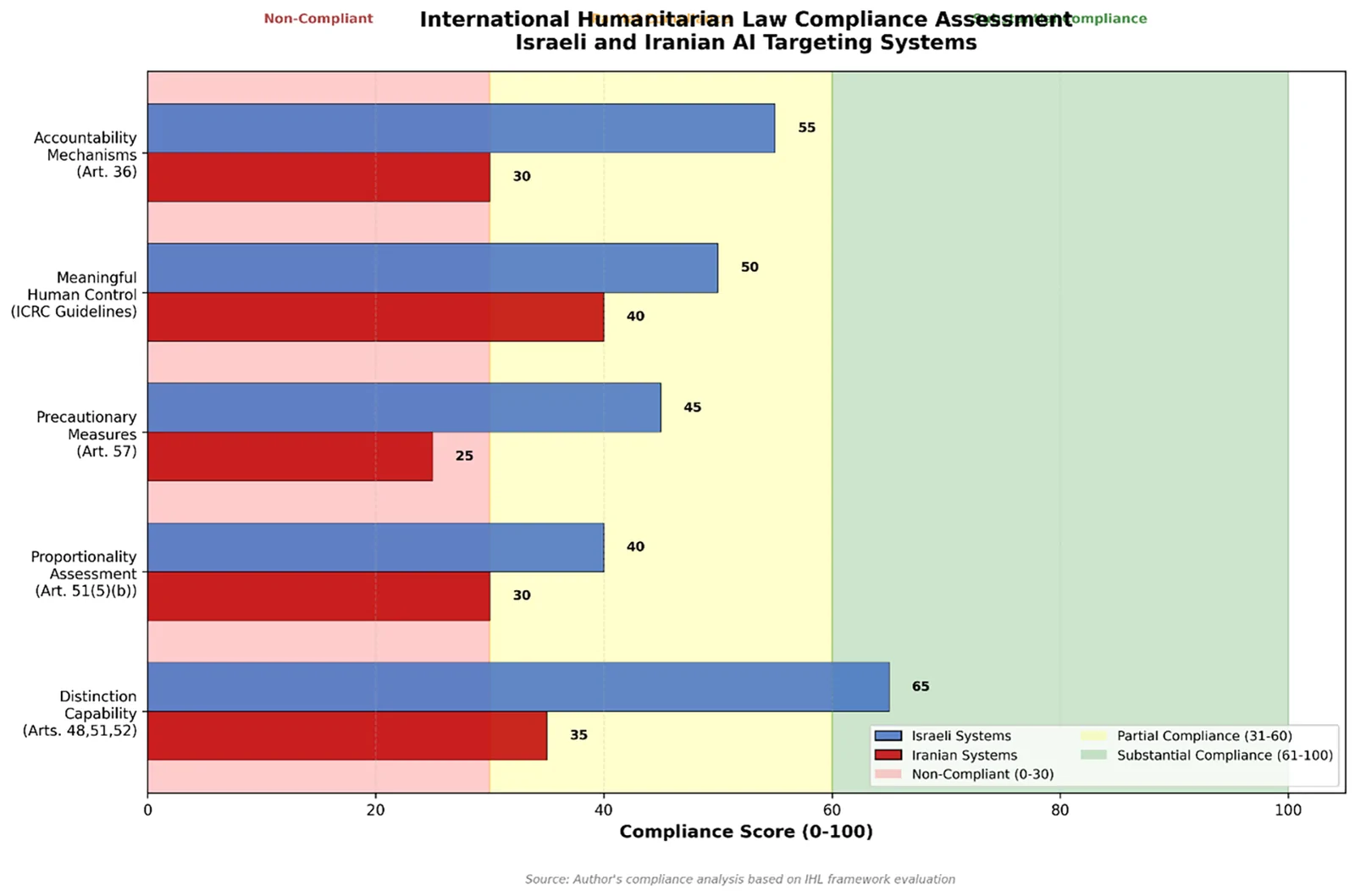
Operational effectiveness comparison.

## 5. Technical and ethical considerations

### 5.1 Algorithmic foundations and technical architecture

The technical application of AI in military targeting systems relies on several core algorithmic approaches, each presenting distinct advantages and limitations in operational contexts. Analysis of documented systems reveals three primary technical paradigms deployed across both Israeli and Iranian implementations.

**5.1.1 Machine learning implementation strategies**

Both countries employ supervised learning algorithms that learn from historical engagement data and target characteristics, though the scope and sophistication of implementations differ substantially.

**Israeli systems (Lavender platform):** Israeli systems demonstrate advanced feature extraction capacity across multi-modal data sources including visual reconnaissance, communication intercepts, and behavioral pattern analysis. System architecture enables real-time processing of diverse data streams, employing sophisticated correlation engines to provide accurate target identification and threat assessment. This multi-modal approach enables cross-validation of target identification across sensor types, increasing accuracy and reducing false positive rates.

The Lavender system specifically employs deep learning architectures trained on extensive datasets compiled from surveillance operations, creating predictive models that identify combatant-associated behavioral patterns. The system’s reliance on correlation rather than causation, however, introduces risks of misidentification when civilians exhibit behavioral patterns statistically associated with combatants.

**Iranian systems:** Iranian applications focus primarily on computer vision-based target recognition and autonomous navigation. Image processing emphasis reflects reliance on open-source computer vision libraries and necessity of operating with limited sensor diversity. To overcome sensor limitations, Iranian systems have enhanced image processing algorithms and pattern recognition capabilities, optimized for specific operational environments where systems deploy.

Iranian autonomous drones employ convolutional neural networks for real-time target identification from aerial imagery, with training datasets developed through operational experience and simulation. The systems demonstrate adaptation to resource constraints through algorithmic efficiency prioritization over computational complexity.

**5.1.2 Neural network architecture and implementation**

The Iron Dome system exemplifies advanced neural network applications in defensive military AI, demonstrating sophisticated real-time processing capabilities essential for ballistic threat interception. Table 4 provides detailed comparative analysis of AI targeting architectures, contrasting Israeli and Iranian implementations across critical technical dimensions.

**Table 4.** Comparative analysis of AI targeting architectures: Israeli vs Iranian implementations.

Technical aspect	Israeli implementation	Iranian implementation
**Processing Speed**	Sub-second response times through optimized convolutional neural networks for trajectory calculation	Focus on efficient processing with limited computational resources, prioritizing reliability over speed.
**Data Processing**	Advanced radar return processing through specialized CNN architectures optimized for ballistic prediction	Emphasis on robust signal processing algorithms that function effectively in contested electromagnetic environments
**Network Architecture**	Deep learning networks with multiple hidden layers for complex pattern recognition and prediction	Simplified network architectures optimized for specific operational scenarios and limited hardware capabilities
**Training Data**	Extensive datasets from real-world engagements and comprehensive simulation environments	Limited training data supplemented by synthetic data generation and simulation-based learning

**Israeli neural network implementation:** Iron Dome employs specialized convolutional neural networks optimized for rapid trajectory prediction from radar data. The network architecture processes radar returns in real-time, predicting impact points and calculating optimal interception trajectories within milliseconds. Training data encompasses thousands of actual engagement scenarios supplemented by extensive simulation data, enabling high prediction accuracy across diverse threat profiles.

**Iranian neural network approaches:** Iranian systems utilize simplified neural network architectures optimized for specific threat scenarios and computational resource constraints. Training data limitations are addressed through synthetic data generation and transfer learning approaches, leveraging publicly available datasets and simulation environments to develop baseline capabilities subsequently refined through operational experience.

**5.1.3 Fuzzy logic systems and decision-making architecture**

Fuzzy logic controllers provide advanced capabilities for managing uncertainty and incomplete information in military targeting systems. Israeli platforms particularly excel in this domain, implementing fuzzy logic engines for target prioritization and engagement decisions under uncertainty conditions.

**Israeli fuzzy logic implementation:** Israeli platforms employ advanced fuzzy logic controllers optimized for urban warfare environments where civilian-military distinction presents significant challenges. These systems enable nuanced decision-making when faced with incomplete sensor data, conflicting information, or ambiguous scenarios. The fuzzy logic model enables graduated responses rather than binary choices, which proves critical in urban settings with substantial civilian-military integration where binary classification systems would fail.

Fuzzy logic enables the system to assign probability scores to target classifications rather than definitive categorizations, incorporating uncertainty into decision recommendations presented to human operators. This approach theoretically enhances civilian protection by flagging ambiguous cases for heightened human scrutiny.

**Iranian adaptive fuzzy logic:** Iranian systems employ simplified fuzzy logic approaches oriented toward operational adaptively within resource-constrained environments. The emphasis is on resilient decision-making algorithms functioning effectively with limited computational resources while maintaining operational effectiveness against diverse threat profiles. Iranian implementations prioritize robustness and operational continuity over sophisticated uncertainty quantification.

### 5.2 Ethical framework analysis

As autonomy in weapon systems increases, the temporal and spatial distance between human operators and selected targets expands, effectively delegating life-and-death decisions to algorithms. This raises fundamental ethical questions about human responsibility in the use of lethal force and the moral dimensions of algorithmic warfare ( Mauri, 2022).

**Distinction principle:** International Humanitarian Law requires clear distinction between combatants and civilians. AI systems must not undermine commanders’ capacity to make required legal determinations regarding target legitimacy. The AI-enhanced Lavender system sparked controversy due to reports that some military leaders treated civilian deaths as acceptable statistics rather than requiring case-by-case proportionality assessments.

According to reports, operational protocols permitted killing up to 100 civilians in bombing raids targeting senior Hamas or Islamic Jihad officials. The system was designed to conduct attacks when targets were inside homes at night, increasing likelihood of successful engagement but substantially increasing risks of killing family members and civilian neighbors.( G.Pascual, 2024). This approach raises serious questions about compliance with proportionality requirements under International Humanitarian Law.

A prominent example is the 2025 killing of journalist Anas al-Sharif in his tent. The Israeli army accused him of Hamas affiliation when bombing his shelter, killing five other journalists in the strike. This incident exemplifies concerns regarding protection of civilians and proper application of distinction principles in AI-assisted targeting ( Mahase, 2025).

Table 5 examines operational integration approaches of AI targeting systems, comparing Israeli and Iranian architectures in command structure, communication systems, and decision authority.

**Table 5.** Operational integration of AI targeting systems: comparative approaches in Israeli and Iranian architectures.

Integration aspect	Israeli approach	Iranian approach
**Command Structure**	Hierarchical integration with human oversight and authorization protocols	Distributed command structure with emphasis on autonomous operation capabilities
**Communication Systems**	Sophisticated communication networks enabling real-time coordination and data sharing	Hardened communication systems designed to operate in contested environments
**Decision Authority**	AI systems provide recommendations, with human authorization required for engagement	Greater emphasis on autonomous decision-making to reduce communication vulnerabilities
**Interoperability**	NATO-compatible systems with standardized protocols for allied integration	Proprietary systems optimized for specific regional operational requirements

**Proportionality principle:** International Humanitarian Law requires that anticipated civilian harm not be excessive relative to concrete and direct military advantage expected from an attack. AI systems must enable commanders to make proportionality assessments, yet compressed decision timelines and high target generation rates may undermine meaningful proportionality analysis. When human operators spend only 20 seconds verifying targets, as reported for Lavender system operations, the capacity for meaningful proportionality assessment becomes questionable.

**Precautionary principle:** Parties to conflict must take all feasible precautions to minimize civilian harm. This includes verification that targets are military objectives, selection of means and methods minimizing civilian harm, and provision of advance warning when circumstances permit. AI systems that prioritize operational efficiency over verification thoroughness may conflict with precautionary obligations, particularly when strikes are conducted in civilian-dense environments or against targets in family residences.

### 5.3 Accountability and legal responsibility

**Command responsibility:** Military commanders remain legally accountable for targeting decisions regardless of AI system involvement. However, the complexity and opacity of AI algorithms may prevent commanders from fully comprehending and validating system recommendations, potentially undermining effective command and control. When AI systems generate targets at rates exceeding human capacity for thorough review, command responsibility becomes difficult to exercise meaningfully.

**Algorithmic transparency:** The “black box” problem of many machine learning systems creates accountability and legal scrutiny challenges. Targeting systems must be capable of legal review consistent with Article 36 of Additional Protocol I to the Geneva Conventions, which requires legal review of new weapons, means, and methods of warfare. When algorithms cannot explain their targeting rationale in human-interpretable terms, such legal review becomes problematic. Table 6 summarizes international legal and ethical frameworks governing AI-based weapon systems, including compliance requirements for military applications.

**Table 6.** International legal and ethical frameworks governing AI-based weapon systems.

Legal framework	Application	Compliance requirements
**Geneva Conventions**	Treaty IHL and Article 36 weapons reviews	Legal review of new weapons systems
**Martens Clause**	Principles of humanity and public conscience	Ethical considerations in system design
**CCW Framework**	Convention on Certain Conventional Weapons	International negotiations on LAWS
**State Responsibility**	International wrongful acts	Liability for IHL violations
**Criminal Liability**	Individual accountability	Programmer/commander responsibility

**Criminal liability framework:** Under international criminal law, programmers may be held criminally liable when designing systems intended to violate International Humanitarian Law, and commanders may face criminal liability when deploying systems incapable of lawful operation. However, proving intent and establishing causal links between algorithmic decisions and unlawful outcomes presents significant evidentiary challenges. The distributed nature of AI system development—involving multiple programmers, data scientists, and military planners—further complicates attribution of individual criminal responsibility. As shown in Figure 5.

**Figure 5.**
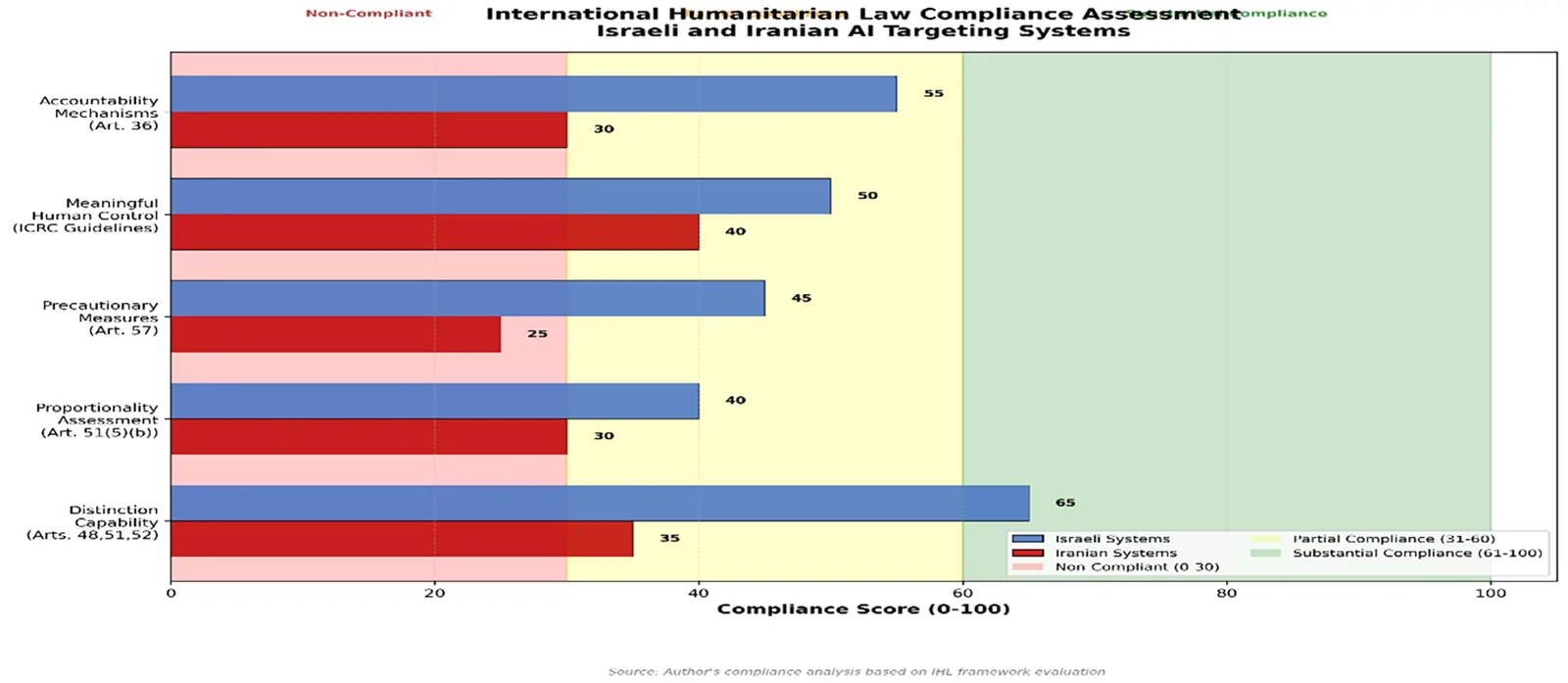
International legal compliance assessment framework.

### 5.4 Civilian protection mechanisms

**Positive target identification:** Effective civilian protection requires reliable identification of lawful military targets and distinction between military objectives and protected persons and objects. Current AI systems demonstrate varying capabilities in this domain, with reported error rates of 5-10% raising concerns about accidental civilian casualties. Even systems achieving 90% accuracy imply substantial misidentification risks when applied at scale.

**Temporal and spatial contexts:** AI systems must account for warfare’s dynamic nature, which introduces factors affecting target legitimacy such as combatants who have laid down arms, hors de combat status, or presence in protected locations like hospitals. Static algorithmic models may not adequately reflect these dynamic legal and ethical considerations. Training data reflecting historical patterns may not capture contextual factors determining lawfulness of engagement in specific circumstances.

**Collateral damage estimation:** Targeting systems incorporate predictive models attempting to estimate civilian casualties from prospective attacks. However, such models rely on assumptions regarding building occupancy, civilian behavior patterns, and weapon effects that may not accurately reflect operational realities, potentially producing inaccurate risk assessments. The “Where’s Daddy?” system’s focus on striking targets in family homes exemplifies prioritization of operational convenience over civilian protection, despite predictable presence of family members and neighbors ( *Israel-Gaza war death toll: Live tracker*, 2025).

## 6. Regional security implications and strategic assessment

### 6.1 Strategic stability and escalation dynamics

Deployment of AI-powered targeting systems in the Iranian-Israeli context introduces novel escalation dynamics that differ significantly from conventional approaches to strategic stability. The automation of target generation and engagement compresses decision-making timelines, potentially reducing opportunities for diplomatic intervention and crisis management. When AI systems identify and prioritize targets within minutes or hours rather than days, windows for de-escalation narrow substantially.

The speed advantage provided by AI systems creates incentives for preemptive action during crises, as states may perceive advantages in striking before adversaries fully mobilize AI-enhanced capabilities. This dynamic introduces crisis instability, where both parties face pressures to act quickly rather than pursue diplomatic resolution. The proliferation of AI military systems without accompanying arms control frameworks risks accelerating regional arms races and increasing conflict probability.

### 6.2 Humanitarian impact assessment

Reported error rates in AI targeting systems (5-10% for most documented systems) translate to substantial civilian casualty risks when applied at scale. The Lavender system’s generation of 37,000 targets with a reported 10% error rate implies potential misidentification of approximately 3,700 individuals. This has been substantiated through increasing civilian and child casualties in Gaza during recent conflicts ( *Israel-Gaza war death toll: Live tracker*, 2025).

According to humanitarian affairs reports and local health authorities, densely populated areas and critical infrastructure including water distribution facilities and residential buildings were repeatedly targeted. This resulted in mass civilian casualties that contradict stated precision targeting objectives. The magnitude of civilian harm raises serious questions about actual operational compliance with International Humanitarian Law principles, despite technological sophistication of deployed systems.

The disconnect between claimed precision capabilities and observed humanitarian outcomes suggests either systematic failures in AI targeting accuracy, inadequate human oversight mechanisms, or operational decisions prioritizing military objectives over civilian protection requirements. Regardless of cause, humanitarian impact assessment indicates urgent need for enhanced accountability mechanisms and independent verification of AI system performance.

### 6.3 Strategic recommendations

Table 7 presents a strategic roadmap for establishing ethical and legal oversight of AI military systems, organized from immediate measures to long-term governance frameworks.

**Table 7.** Strategic roadmap for ethical and legal oversight of AI military systems: From immediate measures to long-term governance.

Immediate measures	Medium-term initiatives	Long-term development
Technical requirements mandating human oversight on all lethal autonomous systems	**Global Discourse:** Multilateral negotiations within existing arms control frameworks	**Legal Framework:** International mandatory treaties on autonomous weapons
Transparency requirements for AI targeting system deployments	**Technical Standards:** International safety standards for AI military systems	**Ethical Guidelines:** Universal principles on ethics of AI in military applications
Independent humanitarian monitoring mechanisms	**Capacity Building:** Enhanced monitoring and verification capabilities	**Enforcement Mechanisms:** International accountability systems for violations
Moratorium on fully autonomous lethal systems	**Regional Confidence-Building:** Information sharing on AI military capabilities	**Compliance Verification:** Independent assessment protocols for AI system compliance

## 7. Conclusions

The integration of artificial intelligence in military targeting systems represents a fundamental transformation in warfare, with implications extending beyond operational effectiveness to strategic stability, international law, and humanitarian protection. Analysis of the Iranian-Israeli conflict demonstrates both the potential and peril of algorithmic warfare, underscoring urgent need for international governance frameworks capable of managing this technology’s trajectory.

The comparison reveals substantial divergences in technological sophistication, strategic implementation, and operational doctrine. Israeli systems demonstrate advanced data integration, precision targeting capabilities, and sophisticated human-machine collaboration architectures. However, concerns emerge regarding compressed human oversight timelines, high-volume target generation potentially exceeding meaningful human review capacity, and operational decisions that appear to prioritize efficiency over civilian protection. Iranian systems reflect asymmetric strategic approaches emphasizing cost-effectiveness, distributed autonomous capabilities, and cyber warfare integration. Resource constraints and technological limitations shape Iranian implementations, resulting in systems prioritizing operational resilience over precision.

Both approaches raise distinct governance challenges. Israeli precision systems demonstrate that even technologically sophisticated AI implementations may fail to ensure adequate civilian protection when operational pressures compress decision timelines and systematic target generation outpaces human oversight capacity. Iranian asymmetric approaches demonstrate that AI military capabilities are accessible even to resource-constrained states, suggesting inevitable proliferation regardless of international control efforts.

Recent Israeli military operations in Gaza highlight the critical gap between claimed precision capabilities and humanitarian outcomes. While official accounts emphasize accurate targeting, unprecedented civilian casualties—particularly among children—substantially exceed losses in previous conflicts. According to humanitarian organizations and local health authorities, attacks on densely populated areas and civilian infrastructure resulted in mass casualties contradicting distinction and proportionality principles. This stark contradiction between stated intent and operational results underscores the necessity of international oversight mechanisms capable of independently verifying adherence to International Humanitarian Law principles.

The international community faces a narrow opportunity window to develop norms, standards, and legal frameworks governing AI military applications while still enabling legitimate defensive capabilities. Decisions made today will largely determine whether AI contributes to international security and humanitarian protection or undermines both in coming decades.

The regulated nature of Israeli systems, emphasizing precision and human oversight in doctrine, suggests potential pathways for responsible AI military development. However, substantial work remains in ensuring that compressed decision timelines do not undermine meaningful human control and that civilian protection maintains priority over operational efficiency. Iran’s asymmetric model demonstrates that AI military capabilities will proliferate regardless of resource constraints, emphasizing urgency of establishing international governance mechanisms before widespread diffusion renders control impractical.

Ultimately, the challenge is not preventing AI military system development—which appears inevitable—but ensuring development and deployment occur in ways preserving meaningful human control, respecting civilian population protection, and maintaining strategic stability. This represents an unprecedented challenge requiring collaboration among technologists, policymakers, legal experts, and the international community. The success or failure of these efforts will fundamentally shape warfare’s future character and humanity’s capacity to maintain ethical and legal constraints on armed conflict in the algorithmic age.

## Data Availability

The datasets supporting the conclusions of this article have been deposited in the Zenodo repository and are publicly accessible at: https://doi.org/10.5281/zenodo.17385265 ( Faris, 2025). The repository contains:
• Structured data extraction tables documenting technical specifications, operational capabilities, and performance metrics of AI targeting systems deployed by Israeli and Iranian military forces • Systematic evaluation matrices assessing systems across six dimensions: data integration, algorithmic architecture, human oversight mechanisms, deployment scale, operational accuracy, and ethical compliance • International Humanitarian Law compliance assessment frameworks evaluating adherence to principles of distinction, proportionality, precautionary measures, meaningful human control, and accountability • Complete source documentation index with reliability assessments and citation trail • Methodological codebooks explaining evaluation criteria and scoring methodologies All supplementary data are released under a CC-BY 4.0 license. This study employs a secondary data analysis approach, systematically reviewing and synthesizing publicly available information from peer-reviewed academic journals, verified investigative journalism, open-source intelligence platforms, technical documentation, and international legal frameworks. No primary data collection, experimental research, or work with human participants was conducted. All source materials are comprehensively cited in the manuscript’s References section and remain accessible through their original publishers. For data-related queries, contact Den.lamia.faris@uoanbar.edu.iq.
