# Algorithmic Targeting in the Iranian–Israeli Confrontation: Technical Realities, Legal thresholds, and the Boundaries of Human Control

**DOI:** 10.12688/f1000research.169794.4

**Published:** 2026-10-09

**Authors:** Lamia faris

**Affiliations:** 1 College of Dentistry, Basic Science, University of Anbar, Ramadi, Al Anbar Governorate, Iraq

**Keywords:** Artificial Intelligence, Military Targeting, Autonomous Weapons, Israeli Defense Forces, Iranian Military Technology, International Humanitarian Law

## Abstract

**Background:**

The adoption of AI for military targeting, defence and engagement operations changes the tempo, magnitude and scope of lethal decision making. Both Iran and Israel have introduced systems that incorporate AI in different ways, technologically and doctrinally, in the Iranian-Israeli confrontation. The issue that is being analysed here is not about which state is technologically more advanced but if meaningful human control of the application of force can be maintained in a high volume, under compressed decision time algorithmic system.

**Method:**

The individual AI-enabled system or application is the primary unit of analysis, whereas Israel and Iran are higher-level comparative contexts used for interpreting the findings at the system level. Systems are first broken down into five functional categories (AI-supported target generation, automated defence interception, assisted fire control at the weapon, autonomous platforms and swarms, and cyber and information applications) based on decision tempo, the location of the human in the decision cycle, and the legal test that must be used. A six indicator assessment is conducted for each system across four dimensions of the system: technical capability, operational employment and effectiveness, human control, and legal compliance. Five indicators are measured as an ordinal capability band with a within band rubric, while legal compliance is not scored but is measured qualitatively. There is an explicit level of confidence attached to each assessment, differentiating between documented and assessed competence. Sources were identified and sourced from peer-reviewed literature, OSINT, official documents and investigative journalism and then prioritized on a five-tiered scale.

**Results:**

The two national sets don't diverge quite as evenly as the aggregate comparison implies within the categories that make up functional sets. The undetermined Iranian capability in target generation is in contrast with the documented high level of Israeli capability. In defensive interception, high automation comes from the physics of the threat. With assisted fire control, AI will be added to the fire control system, but it will only reinforce human control – its authority will be purely negative. For both autonomous platforms and swarms, the capability sets are closest and the control problem is most acute for them. In cyber and information applications the evidentiary asymmetry inverts. Technical accuracy, operational effectiveness, legal compliance and humanitarian outcome are treated as separate constructs throughout.

**Conclusion:**

However, the limitation on meaningful human control is not the sophistication of the algorithm, but the ratio of the number of engagement options that can be provided by a system in a given time to the time taken to interrogate them. Two paths of degraded human control are at work, one from advanced military forces, one from resource-poor ones, both moving toward the same end point. Governance should thus directly control the tempo of decisions, the speed at which decisions pass through, and the review of decisions, and not leave autonomy as an on/off switch on the weapon. A set of 17 specific assignable mechanisms are suggested.

## Key Terminology Definitions

To be consistent throughout this manuscript, the following definitions are taken and upheld:

**Autonomous Weapons Systems (AWS)**: Weapon systems that on activation are able to choose and attack targets without any additional human intervention. This term is also defined as Lethal Autonomous Weapons Systems (LAWS) when discussing the issue of international law.

**AI-supported targeting systems** are military systems that integrate artificial intelligence algorithms to enhance the process of identifying, prioritising, and engaging targets, with obtaining human authorisation such as Lavender and Besorah.

**Defensive Autonomous Weapons Systems**: Autonomous systems developed with defensive functions, including the interception of missiles (e.g., Iron Dome), where the high-tempo threat environment requires automated responses.

**Meaningful Human Control (MHC)**: A criterion that human beings should make knowledgeable and deliberate choices on the lethal force to be employed, and have adequate time and data to make a true judgment. This definition follows the formulation of meaningful human control developed by Roff and Moyes (2015) for the NGO Article 36, and is consistent with the position of the International Committee of the Red Cross on human control in autonomous weapon systems ( ICRC, 2021). It is distinct from Article 36 of Additional Protocol I, which imposes an obligation to review new weapons, means and methods of warfare and is not a source of any definition of meaningful human control.

**Human-in-the-loop (HITL) /human-on-the-loop (HOTL)**: Systems requiring human authorisation of each engagement; systems executing autonomously under human supervision with an ability to intervene within the engagement window.

**Artificial intelligence (AI), algorithmic automation and autonomy**: In this study, a function is treated as AI-enabled only where its outputs are produced by models learned from data (machine learning). Algorithmic automation denotes the execution of rules or algorithms specified in advance, such as trajectory calculation or terrain matching. Autonomy denotes the capacity of a system, once activated, to perform a function such as navigation, target detection or engagement without further human input. These properties are independent: an autonomous function need not involve AI (terrain-matching guidance such as DSMAC is one example), and an AI component need not be autonomous (a target nomination reviewed by a human is one example). AI-assisted targeting and autonomous weapon systems are defined above. Where a source documents automation or autonomy but not machine learning, the system is described accordingly and AI-specific functionality is not attributed to it.

## 1. Introduction

The integration of artificial intelligence in military targeting systems represents one of the most significant technological shifts in modern warfare, fundamentally altering the speed, precision, and scale of military operations ( Gul et al., 2020). In the context of the Iranian–Israeli conflict, both nations have emerged as early adopters of AI-enhanced military technologies, albeit through markedly different strategic approaches and technological implementations. The Israeli Defence Forces (IDF) have developed sophisticated AI platforms capable of processing vast datasets to identify, track, and engage targets at a speed and scale not previously available Systems such as Lavender, which reportedly analysed data on 2 3 million Gaza residents and generated up to 37,000 potential targets, exemplify a paradigm shift toward algorithmic warfare ( Guardian, 2024). In contrast, Iran has pursued an asymmetric strategy, leveraging AI for drone swarm coordination, cyber warfare enhancement, and autonomous weapon systems designed to counter conventional military superiority ( Gilli & Gilli, 2019).

The expansion of AI-enabled military systems raises critical issues related to operational effectiveness, strategic stability, and compliance with international humanitarian law. Recent developments in the military capabilities of both Israel and Iran highlight a fundamental difference in approaches to integrating AI, the implications of which extend beyond regional security to global governance frameworks for autonomous weapons systems ( Davison, 2020).

This technological arms race unfolds in a complex geopolitical landscape where both countries face existential security concerns, resource limitations, and international judgment. The integration of AI into targeting systems raises fundamental issues related to accuracy, responsibility, and compliance with international humanitarian law, especially those concerning civilian safety and the need for human oversight in deadly decision-making processes.

### 1.1 Research aim and scope

Aim To compare individual AI-based military systems and applications deployed or credibly attributed to Israel and Iran in terms of target generation, defending intercepts, helping with fire control, self-controlled platforms and swarms, and cyber and information operations across four analytical dimensions (technical capability, operational employment and effectiveness, human control, legal compliance), determine the form and sufficiency of meaningful human control at different stages and levels of the system and its function, tempo and scale, and implications for the governance of military AI beyond the two cases.

We specify what is compared (focusing on individual systems rather than states, and deliberately extending beyond narrowly defined “targeting systems”, because the governance problem spans all five functions), across which dimensions (four higher-order dimensions operationalised through six indicators, interpreted category-specifically), and for what purpose (not to rank two militaries, but to isolate the conditions under which meaningful human control is preserved, attenuated or lost).

**Scope conditions**

The study includes only publicly reported or credibly reported systems prior to the analytical cut-off. It excludes certain capabilities, such as classified, with no public evidence of, as well as AI applications in logistics, personnel, training and maintenance. It does not reliably adjudicate disputed casualty counts, assign specific attacks to specific systems or make any legal judgments about specific attacks; instead it discusses whether the law will be complied with and then it deals with how the compliance is being carried out, not with the legality of the attack itself, which requires evidence not obtainable from open sources.

### 1.2 Research questions

RQ1 (technical capability): What are the main differences in the technical architecture and operational mechanisms of AI-enhanced targeting systems used in Israeli and Iranian military applications?

RQ2 (operational employment and effectiveness): What are the limitations and strengths of the evidence for operational accuracy and effectiveness for deployment scale?

RQ3 (human control). What are the various forms of human control and under what conditions of volume and decision tempo do they no longer meet the requirements for meaningful human control?

RQ4 (compliance and governance). How well do the compliance architectures of these systems meet the principles of distinction, proportionality, precaution and Article 36 review and what means could fill the gaps found?

### 1.3 Research objectives

The primary objectives of this research are to: (1) provide a systematic comparative analysis of documented AI military systems in Israeli and Iranian defence applications; (2) evaluate the technical and operational characteristics of these systems within their respective doctrinal contexts; (3) assess compliance with international legal frameworks governing autonomous weapons; and (4) derive governance mechanisms from the control failures identified, discussing their implications for regional security as an interpretive extension rather than a tested finding.

### 1.4 Analytical chain and structure of the article

The analysis proceeds through an explicit chain in which each element constrains the next such as in Figure 1 which represents the chain, so that a reader can trace how evidence about individual systems supports the broader conclusions, and identify where they would dissent.

**Figure 1.**
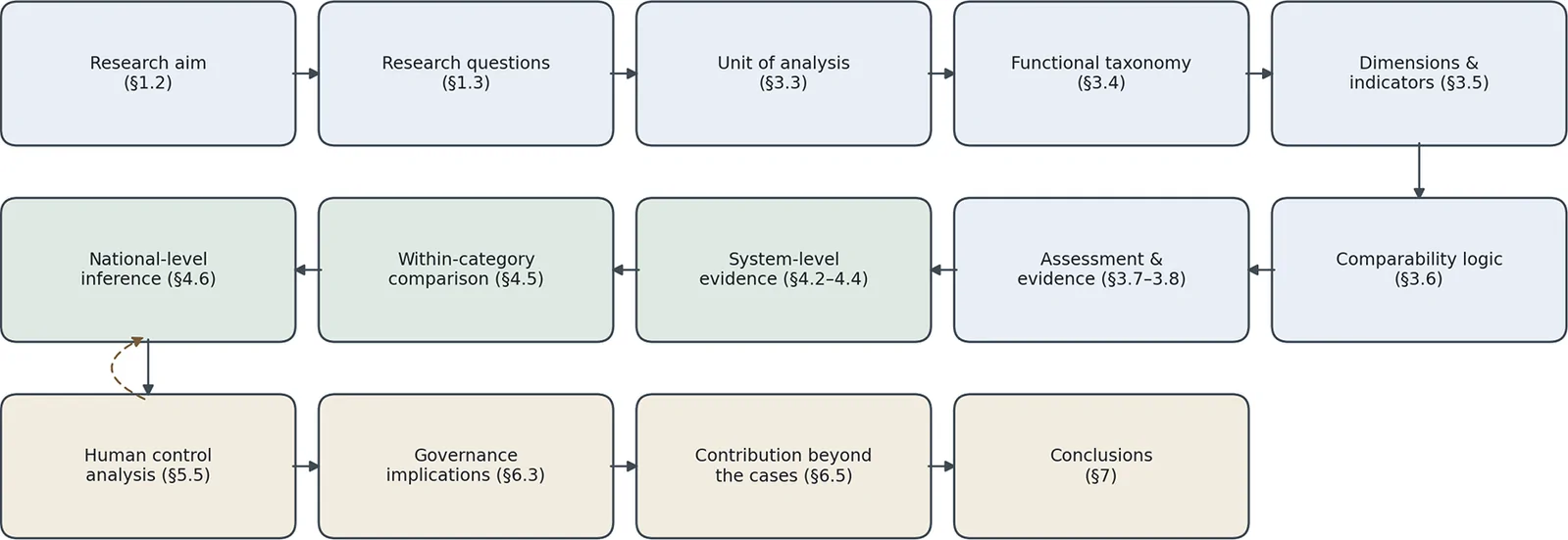
Analytical chain of the study.

## 2. Literature review

### 2.1 Analytical techniques

Academic research in the field of military artificial intelligence has undergone a remarkable transformation over the past decade, progressing from largely theoretical debates about the feasibility and ethics of autonomous weapons to empirical investigations of systems already deployed in active conflict environments. This evolution reflects both the accelerating pace of AI development and its increasingly visible role in shaping modern military doctrine. The growing body of scholarship draws from computer science, international law, strategic studies, and ethics, reflecting the inherently interdisciplinary nature of the subject. Foundational contributions to the field have sought to establish conceptual clarity in a domain prone to definitional ambiguity. In this context ( King, 2024), proposes a conceptual framework that distinguishes between the uses of AI in data analysis, decision support, and autonomous execution, noting that the predominant use of AI in modern military contexts is primarily to enhance intelligence analysis capabilities, rather than to automate combat decisions. This view challenges usual stories that focus on fully autonomous killing machines, instead pointing out AI’s main role in boosting intelligence and speeding up targeting. Scharre (2018) in his foundational analysis of autonomous weapons, offers a taxonomy of human-machine interaction models by dividing between directly decision human-in-the-loop and human-out-of-the-loop systems as examples, proving that the extent of the human control lies on a spectrum, and is not a binary categorization. This paradigm becomes crucial in the analysis of Israeli and Iranian systems that are at the opposite sides of this scale. Horowitz (2019) extends this analysis by examining how military AI adoption patterns vary according to national innovation capacity, arguing that states with robust civilian technology sectors—such as Israel—are better positioned to develop sophisticated AI military applications, while resource-constrained states pursue asymmetric approaches.

The theoretical framework for understanding AI military applications, as identified Scharre (2018) encompasses three primary domains: data processing and analysis, decision support systems, and autonomous execution capabilities. Research indicates that militaries worldwide have prioritized the first two domains, utilizing AI to accelerate and enhance military targeting through improved data fusion and pattern recognition, rather than complete automation of lethal decision-making. Three analytical axes are likewise applied, again as the author’s synthesis rather than as a framework attributable to a prior publication Technical efficiency, which provides for algorithm development and system integration; operational doctrine, which addresses human-machine interaction mechanisms and usage strategies; and strategic impact, which focuses on the role of AI in enhancing deterrence and controlling escalation dynamics. This model provides a basic analytical framework for comparing different systems ( King, 2024). Bode and Huelss (2018) which study the problem of autonomous weapons as a challenge to traditional norms of international security governance, holding that the dissemination of such technologies creates a normative uncertainty that is hard to resolve by current legal frameworks. These contributions collectively establish the theoretical foundation upon which this study’s comparative analysis is built.

### 2.2 Case studies in AI military implementation

Empirical analysis of AI deployment in military contexts reveals diverse implementation strategies across different operational environments King’s research examined two significant cases: the British Army’s COVID-19 testing operation in Liverpool (2020) and the U.S. Security Assistance Group-Ukraine operations (2022), demonstrating how AI systems process multi-source data to optimize targeting and resource allocation ( Emegha & Okoli, 2025). These case studies identified the key principles when it comes to AI usage in the military sphere: the vital role of data quality and integration, the need of human agents to be present in the sphere of strategic decision-making as well as the possibility of the AI systems to greatly increase operational tempo and precision when properly utilised and guided by limits.

The article by Ekelhof (2019) explores the role of autonomous weapons in the modern war by stating that the speed with which AI-based targeting is accomplished radically changes the connection between decision-making and responsibility, ( Sharkey, 2012) notes the challenge of the responsibility gap in lethal autonomous weapons, in which attributing any legal or moral responsibility is difficult when algorithmic systems are involved in the process of making target decisions.

### 2.3 Regional AI military development patterns

Recent scholarly analysis of Middle Eastern military AI development reveals distinct national approaches shaped by strategic priorities, technological capabilities, and resource constraints. Research on Israel’s AI military systems indicates a focus on defensive applications and precision targeting, driven by security imperatives and technological advantages ( Anugrah, 2024). Conversely, Iranian military AI development follows an asymmetric strategy emphasizing cost-effective force multipliers and unconventional capabilities. Studies indicate Iran’s approach prioritizes “rapid deployment of novel technologies, often through unpredictable proxies” as a means of countering conventional military disadvantages ( Lisman, 2021).

### 2.4 Ethical and legal frameworks; meaningful human control, accountability and the legal debate

The debate about autonomous weapons systems in law and ethics has intensified due to the advancement of artificial intelligence. The International Committee of the Red Cross has articulated specific concerns regarding human control requirements and accountability mechanisms in AI-enabled military systems ( ICRC, 2019).

The legal baseline. AI-based systems are not subject to any changes in the targeting rules. Additional Protocol I (1977) distinguishes civilians from combatants and civilian objects from military objectives (Art. 48); prohibits attacks likely to cause incidental civilian harm disproportionate to the expected concrete and direct military advantage (Art. 51(5) b); requires verification of the target and planning for the attack and the choice of means and methods that minimise incidental harm (Art. 57); and stipulates that new weapons, means and methods must be subject to legal review (Art. 36). A residual standard is provided in the Martens Clause. Schmitt and Thurnher (2013) think the rules are fine as long as there is employment, Asaro (2012) and Human Rights Watch (2012) believe that some delegations of lethal decision-making are inherently incompatible with the rules’ premises, whether or not they involve employment. This study does not endorse or oppose prohibition, but shares a common interest in both arguments: the legal tests are directed at human decisions, and the object of the test must be the human decision making process rather than the decision making system.

The control debate. The term originates in policy rather than treaty language, but it has been developed mainly through NGO and academic work: the treaty obligation attaches to the review of new weapons, means and methods of warfare under Article 36 of Additional Protocol I, not to the exercise of control over any particular use of a weapon. The NGO Article 36 — named after that provision, although its work concerns human control rather than weapons review — and Roff and Moyes (2015) introduced meaningful human control to clarify what is needed in addition to nominal human presence: predictable technology, accurate information, timely judgement and intervention, and accountability. This is reworded by Santoni de Sio and van den Hoven (2018) as tracking (the system reacts to relevant human reasons) and tracing (the result follows to a human with suitable moral capacity and knowledge). Ekelhof (2019) suggests that abstract formulations have gotten ahead of operational reality, and that control needs to be spread throughout the multi-stage targeting process, as it is actually conducted by militaries. Where the aim is to identify control requirements by system type, Amoroso and Tamburrini (2020) offer a taxonomy of control requirements based on system type and context that is directly parallel with the taxonomy here. The norms of acceptable control are not being negotiated, but rather are emerging through operational practice, Bode and Huelss (2018) note, suggesting that existing employment patterns in ongoing conflicts are setting the precedent for what is considered acceptable. Accountability and transparency. Sparrow (2007) and Sharkey (2012) provide the concept of the responsibility gap – where the operators of a system would not be able to predict how their system would act; Crootof (2016) counters that the remedy of state responsibility does not require any individual fault and is an underused tool. Horowitz (2019) analyses the stability consequences of speed. The concept that “accuracy” is a technical characteristic of the algorithmic system and that the more important questions of “who” and “why” are disregarded is a point of relevance directly to Section 5.4, as made by Suchman (2020). Boulanin and Verbruggen (2017) chart the technical aspects of autonomy; Holland Michel (2020) makes a distinction between predictability and understandability, and makes the case for operationally relevant transparency rather than interpretability; Ekelhof and Persi Paoli (2020) describe in practice how the human element is dispersed throughout the targeting process.

Accountability and transparency. Sparrow (2007) and Sharkey (2012) view the responsibility gap as one where the operators of a system have no full control over the system’s behaviour; Crootof (2016) argues that there is a lack of understanding of the potential of state responsibility as a remedy in such situations and that no fault need be attributed to any single person. Horowitz (2019) analyses the stability consequences of speed. This is the same point Thisbrook (2020) makes about the notion of ‘accuracy’ in algorithmic warfare, where it becomes a technical characteristic of the system rather than a substantive one of the war. Boulanin and Verbruggen (2017) chart the technical aspects of autonomy; Holland Michel (2020) shows how predictability differs from understandability, and that the notion of operationally relevant transparency is narrower and more attainable than that of interpretability; Ekelhof and Persi Paoli (2020) report on the distribution of the human element in the targeting cycle in practice.

### 2.5 Identified research gaps

While growing, the current literature has several deficiencies that this paper seeks to tackle. First, existing comparative studies of AI military systems tend to concentrate on their technical architecture or legal compliance, with little attempted integration of the two dimensions. Furthermore, even though it is one of the most active theatres for the utilization of AI-enabled military action, the Iranian-Israeli context has benefitted from only limited systematic academic attention. Although major investigations and journalistic pieces on systems like ‘LAVENDER’ and ‘BESORAH’ appeared in the media, an academic analysis contextualising them within existing theories is lacking. The relation of claimed system capabilities to observed humanitarian outcomes have not been studied systematically in the academic literature. Through a structured comparative analysis that integrates technical, operational, ethical and legal dimensions, this study seeks to fill this gap.

There are four gaps in this study that are presented. First, the normative literature on meaningful human control is typically insufficiently empirically grounded in actual operational systems in terms of more than one function, and the comparative literature on military AI in the region is primarily descriptive and seldom connects to the control literature. Second, current comparative evaluations tend to sum up functionally heterogeneous systems, giving results of rankings of unclear analytical value. Third, the link between the volume of targets generated and the need for human review has been observed but has not been formulated as a proposition to be analysed. Fourth, it is well established that there is evidence asymmetry between transparent and opaque military systems that is often recognized as a limitation but is seldom analysed as a variable to account for interpretation of the findings.

## 3. Methodology

### 3.1 Research design

This study employs a structured qualitative comparative framework to investigate the role of artificial intelligence algorithms in military targeting systems within the Iranian–Israeli conflict. The methodology integrates technical system analysis, comparative architecture evaluation, and ethical–legal framework synthesis. More specifically, this study adopts a comparative case study methodology that combines qualitative system analysis with ordinal, rubric-based assessment; reported performance figures are recorded with their source and evidentiary confidence rather than treated as measurements. The research design integrates three analytical approaches: (1) technical evaluation of the system based on documented capabilities and architectural specifications; (2) assessment of operational effectiveness using available performance metrics and deployment patterns; and (3) legal compliance analysis with international humanitarian law standards.

**Design summary**

RQ1 (technical capability) draws on technical documentation, official statements, investigative reporting and OSINT analysis, is measured by the data-integration and algorithmic-architecture indicators (D1), and is answered in Sections 4.1–4.4 and 5.1. RQ2 (operational employment and effectiveness) draws on deployment and performance reporting, is measured by the deployment-scale and operational-accuracy indicators (D2), and is answered in Section 4.4. RQ3 (human control) is measured by the human-oversight indicator (D3), read against the category-specific standard in Table 1, and is answered in Sections 4.4 and 5.4 and Table 8. RQ4 (compliance and governance) is answered by the qualitative classification of documented compliance mechanisms (D4; Figure 6) in Sections 5.2–5.3 and by the governance mechanisms in Section 6.3 and Table 9. The unit of analysis is the individual system; every assessment carries an evidentiary-confidence rating; and the answers to all four questions are drawn together in Section 7.

**Table 1.** Functional taxonomy of AI-enabled military systems and applications.

Category and assigned systems	Primary function	Tempo and human position	Category-specific meaning of accuracy and oversight	Principal legal test
**FC1. Target generation and prioritisation** — Lavender; Besorah	Identify, rank and nominate candidate targets from fused intelligence	Minutes–hours; human authorises each engagement (HITL in form)	Accuracy = share of nominations correctly identifying a lawful objective. Oversight = capacity to interrogate and reject one nomination	
**FC2. Automated defensive interception** — Iron Dome	Detect, discriminate and intercept incoming munitions	Seconds; HOTL at engagement, control via the pre-set envelope	Accuracy = threat discrimination and interception. Oversight = siting, authorisation rules and firing envelope, set in advance	Minimally engaged; object is a munition, not a person
**FC3. Assisted fire control** — SMASH family; IRIS (detection)	Constrain release of a round already aimed at a human-selected target	Sub-second; human selects, aims, initiates; system authority negative	Accuracy = round placement and inhibition of unaimed fire. Oversight = complete at selection; system cannot initiate	
**FC4. Autonomous platforms and swarms** — Goshawk; autonomous UAVs; Iranian loitering munitions, swarm mother-platform architectures, DSMAC cruise missiles	Navigation, coordination, recognition and, in some configurations, engagement after commitment	Seconds–hours post-launch; control expires at commitment absent abort	Accuracy = recognition and engagement against pre-launch parameters. Oversight = pre-launch encoding of target class, geography, time window, plus abort	Distinction; precaution; Art. 36 review
**FC5. Cyber and information operations** — Iranian AI-assisted cyber ops, synthetic media, persona networks; Israeli cyber applications	Effects on networks, data and information environments	Variable; control at campaign rather than action level	Accuracy = effect achieved on the intended system or audience. Oversight = campaign authorisation and effect monitoring	

**Table 8.** Meaningful human control by functional category.

Category	Decision tempo	Feasible form of human control	Component most at risk	Responsibility gap
**FC1** Target generation and prioritization	Minutes–hours per nomination; generation rate may exceed review capacity	Deliberative review per nomination, if throughput stays below certified review capacity	**Time** — collapses under volume (nominal authorisation). **Understanding** — degraded where the basis cannot be reconstructed in the window	
**FC2** Automated defensive interception	Seconds	The deployment envelope: siting, authorisation rules, firing criteria — set in advance	**Capacity to intervene** — intrinsically unavailable, and appropriately so	Narrow — rests with those who set the envelope
**FC3** Assisted fire control	Sub-second; human has already selected and aimed	Complete at selection; system authority is negative only	None materially degraded; the system augments rather than substitutes	
**FC4** Autonomous platforms and swarms	Seconds to hours post-launch	Pre-commitment encoding of target class, envelope and criteria; abort where communications permit	**Capacity to intervene** — expires at commitment. **Understanding** — terminal situation unknowable at decision time	Migrates forward from launch; the decisive act is commitment and its encoded parameters
**FC5** Cyber and information applications	Variable; effects may unfold over weeks	Campaign authorisation with effect monitoring and termination	**Understanding** — effects diffuse and hard to attribute even for the operator	

**Figure 6.**
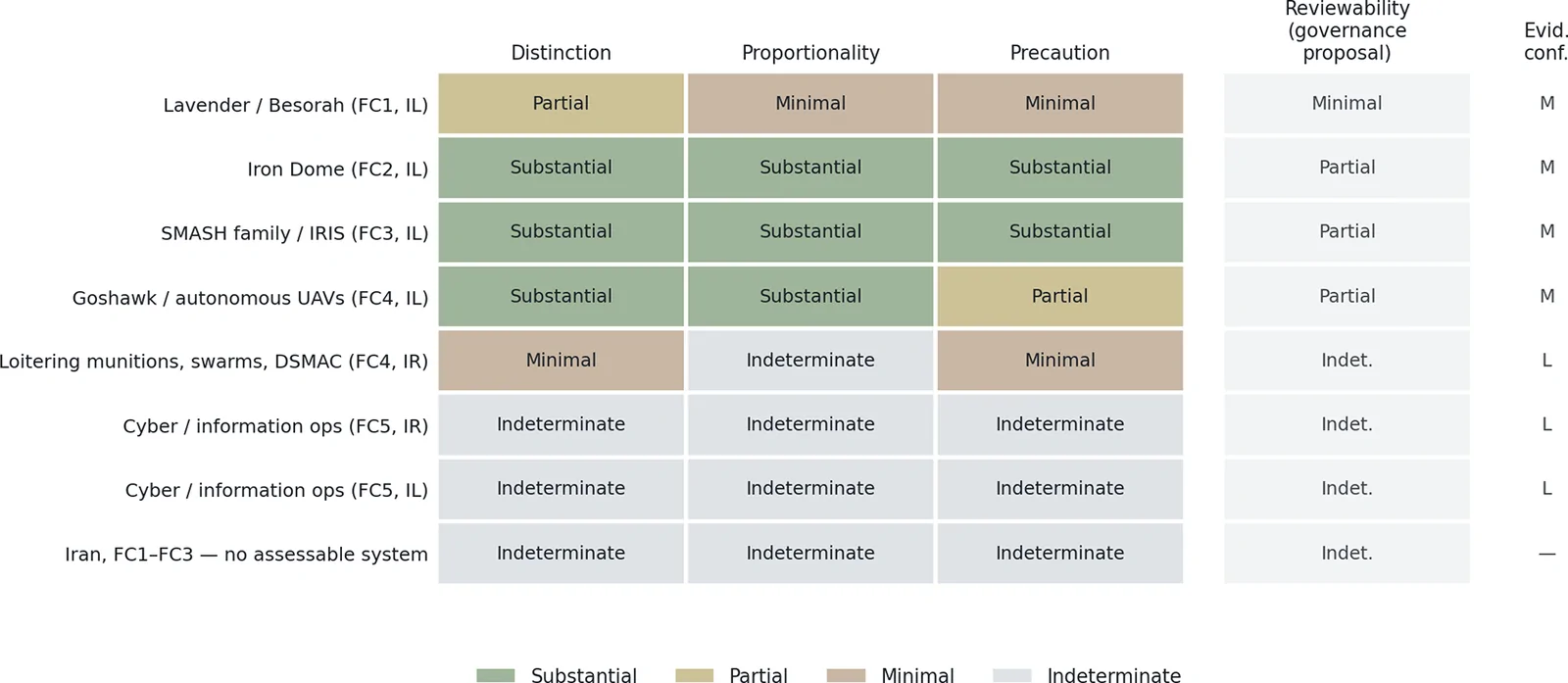
Qualitative assessment of documented compliance mechanisms.

**Table 9.** Seventeen governance mechanisms for AI-enabled military systems.

ID	Mechanism	Actor and instrument	Verification indicator	Feasibility
**A. System design and procurement**				
A1	Reviewability specification: a nominating system must present the principal factors behind each nomination within the review window	Defence ministries; procurement standard	Audit of reconstruction for sampled decisions	High
A2	Throughput ceilings tied to certified review capacity, configured in the system rather than left to practice	Armed forces; configuration + standing order	Nominations per certified reviewer-hour, auto-recorded	High
A3	Minimum deliberation time per nomination class, enforced by withholding further nominations	Armed forces; ROE annex	Timestamped review records, sampled	High
A4	Abort or recall capability for post-launch autonomous systems where communications permit	Defence ministries; design standard	Abort demonstrated at type approval	Moderate
A5	Encoded engagement limits (target class, geography, time window) required before commitment, FC4	Defence ministries; design standard	Parameter record preserved with mission log	High
**B. Operational employment**				
B1	Organisational separation of nomination from strike authorisation	Armed forces; command directive	Audit of separate reporting chains	Moderate
B2	Reviewer certification in the failure modes of the system reviewed, periodically renewed	Armed forces; training standard	Certification and examination records	High
B3	Recording of collateral damage estimation inputs and assumptions per engagement	Armed forces; targeting directive	Auditable post-operation record	High
B4	Automatic escalation requiring command re-authorisation when throughput exceeds review capacity	Armed forces; standing order + system flag	Escalation log and disposition	Moderate
**C. Legal review and accountability**				
C1	Article 36 review extended to employment tempo, throughput and review protocol	AP I states party; national procedure	Scope documented in national procedure	Moderate
C2	Re-review on model update or retraining; version-controlled model registry	States; national procedure	Registry with review status per version	High
C3	Independent technical investigation access after incidents of significant civilian harm	States with ICRC or agreed body; protocol	Access granted or refused, recorded	Low–Moderate
C4	Preservation of targeting logs, nomination records and review timestamps	States; obligation + third-party audit	Audit of retention and integrity	Moderate
**D. International coordination**				
D1	Definitional standard by functional category, so obligations attach to function not to “autonomy”	CCW GGE; declaration then protocol	States adopting the standard	Moderate
D2	Transparency register of national human-control policies by functional category	UNGA or CCW; reporting mechanism	Submissions filed	Moderate
D3	Prohibition on autonomous engagement of persons on pattern-of-life classification alone	CCW; protocol or new instrument	Ratification count	Low
D4	Reciprocal notification of deployment envelopes of AI-enabled defensive systems	Regional states; CBM agreement	Notifications exchanged	Low–Moderate

### 3.2 Data collection and sources

Information on conflict areas is both extremely biased and imprecise due to restricted access to the field, the prevalence of propaganda, and the absence of comprehensive ground-truth verification. Bias may manifest in source selection when reporting disproportionately reflects one side’s narrative, or when unrecorded events are omitted from casualty statistics. This distortion may also occur intentionally, as governmental and non-governmental entities may release false or misleading information, resulting in the overstatement or understatement of AI system accuracy and effects. To counter such problems, this research employs a cross-checking methodology across numerous source types, including peer-reviewed literature, open-source intelligence (OSINT) platforms, military data, and investigative journalism. Sources supported by verifiable evidence such as photographs, leaked documents, and confirmed eyewitness testimony are given priority. In the event of contradictions between sources, the contested nature of the information is clearly noted, and no definitive judgments are made based on a single source. The data collection process employed various source categories to ensure comprehensive coverage. The principal categories of sources were as follows:

• Academic database: Web of Science, Scopus, JSTOR, Google Scholar.
• OSINT platforms: Bellingcat, Jane’s Defence Intelligence, OSINT Techniques, Global Security, Center for Strategic and International Studies (CSIS), RAND Corporation, Stockholm International Peace Research Institute (SIPRI).
• Authority documents of the government and the army: Israeli Ministry of Defense publications, IDF official statements, Iranian state media (with appropriate verification caveats), U.S. Department of Defense reports.
• Investigative journalism outlets: The Guardian, +972 Magazine, Al Jazeera Investigative Unit, New York Times, Washington Post.
• Legal and humanitarian sources: ICRC publications, UN reports, Human Rights Watch, Amnesty International.

**3.2.1 Language limitations**

English, Arabic, and Hebrew sources were used. English translations or summaries of respectable analytical institutions were incorporated in the sources in the Persian language. This restriction can lead to bias in favor of sources that are available to Western researchers, and this has been admitted in the limitations section.

**3.2.2 Inclusion criteria**

Studies were included in case they contained the following criteria:

Technical accounts: Documents that give technical specifications, architecture or operational parameters of AI-enabled military systems that are detailed enough to be compared with other systems.

Evidentiary support: Sources that are backed by verifiable facts like photographs, leaked documents, eyewitness testimonies confirmed, or official admissions.

Source credibility: Published by an accepted academic institution, reputable media outlet, editorial standards of quality, well-known research organisation or official government/military source.

Cross-verification: Data that has been supported by two or more independent sources, unless it is mentioned as being a single-source claim.

**3.2.3 Exclusion criteria**

The exclusion criteria were that sources must:

- Derived from information whose authenticity is misinformed or propagated without corroboration.
- Only speculative or theoretical abilities and not recorded.
- Were not attributable to known persons or schools.
- Had serious internal contradictions that it could not explain.

### 3.3 Unit of analysis and levels of inference

The primary unit of analysis is that of the individual AI-enabled military system or application – Lavender, Besorah, Iron Dome, the SMASH family of fire-control systems, IRIS, Goshawk, Iranian loitering-munition systems, Iranian AI-enhanced cyber and information operations. Assessment, scoring and evidentiary rating is done at this level.

The system-level findings are interpreted in the higher-level comparative context of Israel and Iran. Statements at this level describe approach, which refers to the pattern of assessments of technological options, doctrinal preferences and resource constraints that can be seen within the assessed systems, rather than the capability of the nation as a whole.

Rule of attribution. All conclusions are indicated by the level on which they are warranted. There is no national-level assessment that is derived from system-level assessments and presented as a national-level assessment. If evidence leads to a system-level conclusion, but does not lead to the generalisation, it is explicitly made clear that this is not the case.

### 3.4 Functional taxonomy of the systems compared

The systems studied are functionally heterogeneous. Lavender identifies and prioritises targets, and human oversight consists of review and authorisation of a proposed target. A response may be needed in seconds, and speed and automation are intrinsic factors in Iron Dome intercepting incoming munitions. Navigation, coordination, target recognition and potentially engagement, are provided by an autonomous platform or swarm. Cyber and Information applications are not targeting physical entities, but networks, data and populations. For instance, if you apply the same dimensions to these unqualifiedly, you get spurious comparability: “human oversight” and “operational accuracy” refer to vastly different constructs between them.

There are two decisions of classification that need to be explained. Goshawk’s autonomy level/target acquisition place it in the FC4 and not the FC2 because the level and focus of autonomy in target acquisition dictate the level of human control over the weapon and the legal issues raised, and the purpose of defence does not. SMASH-family fire control is not added to FC1, but to FC3, since it does not create or propose targets, it simply restricts release of a round that has already been fired by a human that is aimed at a human-selected target, thus decreasing the number of engagements the human is able to initiate.

### 3.5 Analytical dimensions and indicators

There are four higher-order dimensions which are each used to solve a different analytical question. Data integration and algorithmic architecture are components of D1 Technical capability — what the system can do technically. D2 Operational employment and effectiveness — how it is used and whether it has the intended effect — includes deployment scale and operational accuracy. Human control (D3) is measured by the banded human-oversight indicator, read against the category-specific standard in Table 1 and complemented by the qualitative assessment in Table 8; legal compliance (D4) is classified qualitatively against four requirements and is not banded. However, the four dimensions are not added together to form an over-arching technical capability index, as this would conceal the very relationships this study is concerned with, and as high technical capability does not require high operational effectiveness nor compliance with law. There is no composite score that is calculated at any time. The readings are taken on a category-by-category basis for each indicator. Data integration is the population-scale ingestion of surveillance data in FC1, the latencies between sensor and effector in FC2, and sharing on board and between platforms in degraded communications in FC4. In FC1, algorithmic architecture is related to ranking models, while in FC2, it is related to trajectory prediction and discrimination, and in FC4, it is related to computer vision and coordination policies. Human oversight isn’t witnessed as “more human involvement is better”, but rather the right form is within the category, and the assessment records the form and sufficiency of oversight relative to the tempo of that category, not simply the presence of oversight. Scale records of deployments include extent of deployment and/or volume of target generation or number of engagements. Operational accuracy refers to the accuracy of the main output of the system in the following categories: correct nomination (FC1); threat discrimination and interception (FC2); round placement and inhibition of unaimed fire (FC3); target recognition and engagement (FC4); effect achievement (FC5). Legal compliance is not banded or scored (like the other 5 indicators) but is treated separately. International humanitarian law principles are subjective and qualitative, and vary according to the circumstances of each attack; imputing a linear value to the compliance with the obligations under Article 51 and 57 of Additional Protocol I would give the impression of precision and comparison, which this evidence does not warrant, even if a value is attributed to it as an analytical assessment. The legal compliance is therefore reported as a qualitative classification against four requirements, which are given in Section 3.7 and applied in Figure 6.

Final analytical model. The same model is used throughout: four higher-order dimensions (D1 technical capability, D2 operational employment and effectiveness, D3 human control, D4 legal compliance); five indicators assessed in ordinal bands of Low, Moderate or High (data integration, algorithmic architecture, deployment scale, operational accuracy and human oversight); a qualitative classification of documented compliance mechanisms; and an evidentiary-confidence rating attached to every assessment. The “six indicators” counted in Table 6 are these five banded indicators plus the compliance classification. Numerical values on a 0–100 scale used in earlier versions are retained in the supplementary repository only as superseded analytical inputs, not as validated measurements.

**Table 6.** Assessed system set and banded indicator assessments.

System (functional category, state)	Ind. Assessable	Data integ.	Algorithm	Human oversight	Deploy. scale	Accuracy
**Lavender/Besorah** (FC1, IL)	6/5	H up (H)	H mid (M)	L up (M)	H up (H)	M (L)
**Iran, FC1** — none assessable	—	und.	und.	und.	und.	und.
**Iron Dome** (FC2, IL)	6	H up (H)	H mid (M)	L (H) *	H up (H)	H low (M)
**Iran, FC2** — none assessable	—	und.	und.	und.	und.	und.
**SMASH/IRIS** (FC3, IL)	6/5	M low (H)	M up (H)	H up (H)	H low (M)	H mid (M)
**Iran, FC3** — none assessable	—	und.	und.	und.	und.	und.
**Goshawk/autonomous UAVs** (FC4, IL)	5	H low (M)	H low (M)	M low (M)	M (L)	H low (L)
**Loitering munitions, swarms, DSMAC** (FC4, IR)	6/5	M low (M)	M up (M)	L mid (M)	H low (H)	M (L)
**Cyber/information ops** (FC5, IR)	6	H low (M)	H low (M)	M (L)	H mid (M)	M (L)
**Cyber/information ops** (FC5, IL)	4	M–H (L)	M–H (L)	M (L)	M–H (L)	M (L)

### 3.6 Comparability logic

Comparisons are done within functional categories, and Section 4.5 is organised in that way. Statements across categories are not made at indicator level, across different categories of indicators, and they are not statements, but interpretations. If a comparison is based on documented evidence for one system and inferred evidence for the other, then the confidence rating is given for both the documented and the inferred evidence.

Two non-comparabilities are stated here instead of being implied from a common score. Numbers are not simply equivalent between categories: no matter if the interception rate is 90 per cent or the rate of correct nominations is 90 per cent, there will be no denominator and the consequences for civilians will be different in each case. Human oversight is likewise incomparable: If an interceptor has a several-second engagement window with a nominal confirmation of a human target nominated by an algorithm, this is a different situation from the previous one, and the latter is more compliant with meaningful human control.

### 3.7 Assessment procedure: capability bands with a within-band rubric

**Change from earlier versions.** Versions 1–3 reported assessments on a 0–100 scale. Version 3 clarified that these were analytical assessments rather than validated measurements, but that clarification is insufficient: point values such as 65, 75 or 85 imply a discriminating power that open-source evidence about active military systems cannot support, and the manuscript could not specify what a ten-point difference within a band substantively meant.

**Revised procedure.** Assessment is reported ordinally. The primary result for each indicator is a capability band — **Low**, **Moderate** or **High** ( Table 2). Within a band, a qualifier of **lower**, **middle** or **upper** is assigned only where justified against the rubric in the final rows of Table 2; where it cannot be justified, the band alone is reported, and this is recorded as a result rather than a gap to be filled by estimation. Within-band position reflects the number of band criteria satisfied and whether satisfaction is documented or inferred; no arithmetic is performed on it.

**Table 2.** Capability band definitions and within-band placement rubric for the five banded indicators. Read together with Table 1: the same band label denotes a different substantive condition in different functional categories.

Indicator	Low	Moderate	High
Data Integration	Single or limited data sources; minimal real-time capability	Multiple data sources with partial integration; some real-time processing	Comprehensive multi-modal fusion; full real-time processing across sensor types
Algorithmic Architecture	Rule-based systems; limited ML application	Standard ML algorithms; moderate neural network depth	Advanced deep learning; sophisticated predictive models; multi-task architectures
Human Oversight	Minimal oversight; autonomous execution without authorisation requirements	Human-on-the-loop with intervention capability; time-constrained review	Human-in-the-loop with meaningful review time; robust override mechanisms
Deployment Scale	Limited deployment; experimental or pilot programs	Operational deployment in specific contexts; moderate target generation	Large-scale operational deployment; high-volume target generation
Operational Accuracy	Reported or assessed correctness low relative to category norms; no validation; error characteristics undocumented	Reported or assessed correctness moderate; partial validation; limited independent verification	Reported or assessed correctness high; validation protocols documented; some independent verification available
**Within-band position**	Threshold met; one or more secondary band criteria unmet or only partly met	Threshold plus most secondary criteria met, at least one documented at level (a)-(c)	All band criteria met and documented at level (a)-(c); approaches the boundary of the band above
**Band reported alone**	Assigned wherever the evidence supports the band but does not discriminate position within it. This is a result, not a gap to be filled by estimation		

**Treatment of the earlier numerical values.** These have been moved to the Zenodo supplementary dataset, where they appear explicitly as ordinal ranking inputs rather than measurements, alongside the evidence supporting each assignment ( Faris, 2025). Figures in this article use banded axes.

**Qualitative classification of legal compliance.** Legal compliance is classified, not banded, against four requirements assessed separately: *distinction mechanisms* (how the system or its employment operationalises the identification of lawful military objectives); *proportionality process* (whether and how the expected incidental harm is weighed against anticipated military advantage, and by whom); *precautionary measures* (what is done to verify targets, choose means and methods minimising incidental harm, and cancel or suspend where required); and *reviewability* (whether the basis of an engagement can be reconstructed, in the moment by the decision-maker and afterwards by an investigator, including for the purposes of Article 36 review). Each is classified as:

• **Substantial** — mechanisms operationalising the requirement are documented and structurally adequate to the system’s function and tempo;
• **Partial** — mechanisms are present but incomplete, undocumented in material respects, or inadequate to the tempo at which the system operates;
• **Minimal** — mechanisms are largely absent, or documented practice is difficult to reconcile with the requirement;
• **Indeterminate** — the available evidence does not permit classification, or the requirement is not engaged in the form in which it applies to kinetic targeting.

These are ordinal in an ordinary-language sense only. They are not converted to numbers, not aggregated, and not compared across functional categories except where the accompanying narrative states the basis for doing so. An Indeterminate classification is a statement about the evidence, not a finding of non-compliance. No classification in this study is a finding that any particular attack was or was not lawful (Section 1.1).

**Inferential limits.** These classifications assess the documented compliance architecture of a system: whether its reported design and employment procedures provide the mechanisms through which distinction, proportionality and precaution can be applied by human decision-makers. They are not determinations that a system, or any attack in which it was used, was lawful or unlawful. The rules of distinction, proportionality and precaution bind those who plan and decide upon attacks, in the circumstances of each attack; they are not properties of an algorithm. Proportionality in particular is assessed before an attack, against the expected incidental civilian harm and the concrete and direct military advantage anticipated from that attack, and cannot be inferred from a system’s general accuracy. Article 36 of Additional Protocol I concerns the legal review of new weapons, means and methods of warfare before their adoption; reviewability is assessed here because it conditions both that review and any later investigation, not as a fourth targeting rule. Four questions are therefore kept apart: whether a weapon, means or method has undergone Article 36 review; whether a system’s design can support compliance in a given context; whether reported employment procedures appear compatible with distinction, proportionality and precaution; and whether a specific attack was lawful. The classifications in Figure 6 address only the second and third, against the principal legal test identified for each functional category in Table 1, and a human authorisation step does not by itself make a system compliant.

**Single-rater limitation.** All assessments were performed by one researcher and inter-rater reliability testing was not possible. The rubric structures and documents these judgements but does not eliminate their subjectivity; the findings most sensitive to source interpretation are identified in the sensitivity note in Section 4.5. Three mitigations apply: the rubric in Table 2 was applied consistently and its application recorded in the supplementary dataset; the source hierarchy in Section 3.8 governs which evidence may support which band; and the move from point scores to bands reduces the surface on which unexamined subjective precision can operate. The limitation is not eliminated (Section 6.4).

(a) Technologically independent confirmation of the technical documentation;

(b) Government/military records which have been proved true;

(c) Verifiable investigative journalism; names on the sources;

(d) OSINT performance of a developed establishment;

(e) Source reporting anonymously (with sufficient reservations).

**Conflict notation.** In case of any serious disagreement despite the implementation of the hierarchy, conflicting claims are clearly stated in the text using suitable uncertainty language.

**Minimum estimation.** In situations where the quantitative estimations were contradictory, the lesser estimation was used unless there was particular evidence of greater estimations. This five-level hierarchy also serves as a source reliability classification system. Throughout the manuscript., All results are linked to their evidence category throughout the manuscript to enable readers to assess evidentiary weight. Claims backed by Level (a) or (b) sources are presented with more certainty, while those relying mainly on Level (d) or (e) sources are accompanied by proper hedging language like “according to reports,” “allegedly,” or “as claimed by anonymous sources.” Where findings are based solely on disputed or partly corroborated sources, this is indicated explicitly.

The information from ongoing conflict areas is systematically biased and not precise because of limited field access and classification, propaganda and the lack of ground-truth verification.

**Source priority hierarchy.** Where sources conflict: (a) technically independent confirmation of technical documentation; (b) verified government/military records; (c) attributable investigative journalism; (d) OSINT assessment by an established institution; (e) anonymous source reporting, as per reservations. If there is disagreement, disagreeing claims are represented in an uncertain manner; if there is quantitative disagreement, the lesser is used unless there is specific evidence for the higher.

**Documented, assessed, and evidentiary confidence.** Evidential asymmetry in both cases is seen as an analytical condition that affects interpretation rather than just as a limitation at the end. Documented capability is what can be established at levels (a)–(c). A reasonable interpretation of the available evidence including levels (d)–(e) is assessed capability when there is no direct evidence. Evidence of confidence is the degree of confidence that seems like a difference between the systems is not due to their differing transparency. Each of the assessments in Section 4.5 is given a confidence rating, as noted in Table 3.

**Table 3.** Evidentiary confidence criteria.

Evidentiary confidence	Assignment criteria	Effect on interpretation of the assessment
**High**	Assessment rests on technical documentation, official acknowledgement, or attributable investigative reporting corroborated by at least two independent sources (hierarchy levels a–c)	
**Medium**	Assessment rests on institutional OSINT analysis or single-source attributable reporting, consistent with but not confirmed by other evidence (level d, or a single level c source)	Assessment may be compared within category with the confidence rating stated; supports national-level inference only in combination with other evidence
**Low**	Assessment rests on manufacturer or operator claims, anonymous sourcing, or inference from structural conditions (level e, or inference)	

**Interpretive rule.** What is not done is to attribute lower capability to a lower visibility of Iran’s capabilities. When an Iranian system is measured at a lower band, the text indicates whether the system is measured with apparent ability gaps but with convergent evidence or greater uncertainty about the system being measured, which is reported as a bounded assessment with Low confidence and no national level inference. The same applies to the other side: missing Iranian paperwork like there is for Israeli human oversight is not a sign of a lack of similar problems.

### 3.8 System selection

Those systems included were those that met all of: (i) evidence of operational deployment or advanced development by Israeli or Iranian forces; (ii) a documented or credibly reported AI component material to the system’s function; (iii) a sufficient evidentiary basis for assessment of at least four of the six indicators; and (iv) assignability to a functional category. The boundary of the assessed set can be seen for systems that satisfy (i) and (ii) but (iii). The assessed set intentionally covers the five categories across both states, and not necessarily the top categories in each state as this would create the deviation that the study documents.

## 4. Results

### 4.1 AI systems in Israeli military operations

**4.1.1 Lavender system**

The Lavender system represents one of the most advanced applications of AI-driven target identification in modern warfare. Lavender is reported to be a machine-learning system developed by the elite Unit 8200 in the IDF that uses mass surveillance data of the approximately 2.3 million people of Gaza to produce individual threat scores between 1 and 100, according to the convictions of the Israeli army, based on behavioural patterns, communication networks, and movement patterns ( Shabbir, 2024; Guardian, 2024).

**Technical architecture:** Lavender is built on a multi-layered data fusion system that integrates visual surveillance, cellular communications, social media activity, and battlefield intelligence. The system uses supervised learning to identify patterns associated with militant profiles, analyzing features indicative of combatant status such as WhatsApp group memberships, frequency of address changes, and communications with known targets ( Shabbir, 2024). The machine learning model was trained on historical data from confirmed combatants and refined through iterative validation processes.

**Operational capabilities:** During peak operational periods, Lavender generated approximately 37,000 potential human targets. According to IDF sources, the system achieved approximately 90% accuracy in target identification; this is an unvalidated operator claim whose denominator has not been specified (Section 4.4.1). System outputs feed directly into operational planning, with human operators spending an average of 20 seconds per target verification, primarily to confirm gender rather than to scrutinize targeting rationale or evaluate civilian risk factors.

**Performance metrics:** Unit 8200 internal validation studies suggested that Lavender could reach approximately 90 percent accuracy in detecting valid military targets. This figure has been challenged by external analysts who note that the system relies on statistical correlation rather than verified intelligence.

Civilian casualties in Gaza have been extensively reported, but casualties do not by themselves establish algorithmic inaccuracy, and the relationship between the two is not determinable from the available evidence (see Section 5.4). What is reported is that the system does not itself account for whether nominated individuals are among civilians or near children at the time of engagement, which presents serious ethical and legal concerns regarding compliance with distinction and proportionality principles in International Humanitarian Law.

**4.1.2 Besorah: Accelerated target production**

The Besorah system serves as a complementary platform to Lavender, focusing on infrastructure and high-value target identification through automated analysis of drone imagery, intercepted communications, and behavioural pattern recognition. According to published reporting, Besorah can generate up to 100 prioritized targets daily, utilizing advanced computing and machine learning algorithms to evaluate target significance and strike feasibility ( Gul et al., 2020).

Algorithmic approach: Besorah employs a traffic-light classification system (red/yellow/green) to prioritise targets based on military value, civilian risk assessment, and operational feasibility. The system integrates real-time intelligence feeds with historical pattern analysis to predict target behaviour and optimal engagement windows. This predictive capability enables proactive targeting while theoretically maintaining consideration of civilian protection requirements.

**Integration with human oversight:** Unlike fully autonomous systems, Besorah maintains human command authority for final targeting decisions. Military commanders review system recommendations and must explicitly authorize strikes. However, compressed decision timelines—often minutes rather than hours—limit the depth of human analysis possible. This temporal constraint raises questions about the meaningfulness of human oversight when system-generated recommendations arrive at rates exceeding thorough human evaluation capacity.

**4.1.3 SMASH family: Precision engagement systems**

The SMASH (Smart Shooter) family represents AI-enhanced fire control systems designed for precision engagement across multiple platforms. These systems combine computer vision, predictive algorithms, and automated fire control to enhance accuracy and reduce collateral damage in complex operational environments ( Soldier Systems Daily, n.d.).

**Technical components:** SMASH systems integrate several AI-driven capabilities:

• **Target acquisition algorithm:** Employs computer vision to identify and classify potential targets in real-time, distinguishing between combatants, civilians, and objects.
• **Tracking algorithm:** Continuously computes target trajectory while accounting for environmental variables including wind, humidity, and target velocity.
• **Fire-release control:** The shooter selects the target, aims and holds the trigger; the system releases the round only when the fire-control solution predicts a hit, and otherwise withholds it.

Table 4: presents the technical specifications and key features of the SMASH family components.

**Table 4.** SMASH technical components.

SMASH variant	Platform	Caliber support	Key features
SMASH 2000	Handheld Extension	5.56 mm, 7.62 mm	Individual soldier targeting system
SMASH Hooper	Remote Controlled	5.56 mm, 7.62 mm	Safe-distance operation capability
SMASH Dragon	Heavy Weapons	40 mm Grenades	Vehicle and UAV integration

**4.1.4 Iron Dome: Predictive defence architecture**

The Iron Dome system exemplifies automated defensive decision support, predicting incoming projectile trajectories and calculating interception parameters. Radar data is processed algorithmically to distinguish between threatening and benign projectiles, with engagement resources allocated only to genuine threats.

**Algorithmic components:**

Algorithmic components are essential for enhancing threat response capabilities in modern autonomous defence systems:

1. **Trajectory prediction models:** Algorithms calculate projectile paths by integrating ballistic properties with dynamic environmental conditions
2. **Threat assessment algorithms:** Automated systems analyse impact probabilities and expected damage severity to prioritise engagement decisions
3. **Interception optimization:** Real-time algorithms determine optimal timing and targeting strategies for interceptor deployment, maximizing accuracy while minimizing collateral risks

**Performance metrics:** The Israeli military reports that Iron Dome achieves an interception rate of approximately 90% for short-range rockets and mortars; this is an official claim, and both the rate and its method of calculation have been contested in independent analysis. A secondary source reports, without independent verification, that the interface reduces the time required for threat assessment and engagement by approximately 40% compared to previous systems, enabling faster response to evolving threats. The system’s selective engagement algorithm reduces operational costs by avoiding interception of projectiles predicted to land in uninhabited areas ( Ramachandra, 2023).

**System limitations:** Despite high performance, Iron Dome faces several constraints. Each interceptor missile is reported to cost approximately $40,000–$50,000, although published estimates vary considerably, making sustained use financially burdensome during prolonged or frequent attacks. While highly optimised for short-range threats, the system demonstrates reduced effectiveness against long-range ballistic missiles or complex multi-directional assault patterns. The system can be overwhelmed by high-volume simultaneous rocket launches, potentially reducing interception coverage and effectiveness. The October 2023 Hamas assault and subsequent Iranian-Israeli exchanges demonstrated that Iron Dome’s interception range and effectiveness can be degraded under sustained, high-volume attack conditions ( Al Jazeera, n.d.).

**4.1.5 Specialized systems: IRIS and Goshawk**

Two further systems illustrate the trajectory toward autonomous platforms.

Recent advancements in autonomous robotics have led to specialized systems for reconnaissance and counter-drone operations:

**IRIS reconnaissance robot:** Features AI-driven surveillance capabilities in confined spaces through computer vision algorithms that identify personnel, objects, and explosive devices. The system is compact and lightweight (1.85 kg, 20 × 23 × 11 cm dimensions), with 200 m communication range, 5 km/h maximum speed, and 1 kg payload capacity. IRIS enables reconnaissance in urban environments where human entry presents elevated risk.

**Goshawk autonomous interceptor:** Described by its manufacturer as an autonomous counter-unmanned aerial vehicle (UAV) system, supervised at mission rather than engagement level. It includes day and night detection capabilities, non-lethal drone capture methods, and smart nest recovery systems, allowing interception without collateral damage or continuous human involvement. These platforms collectively indicate evolution toward integrated, precision-oriented robotic systems emphasizing scalability and operational adaptability.

### 4.2 AI Deployment in Iranian defence strategy

**4.2.1 Strategic asymmetric approach**

Iran’s military AI development follows a fundamentally different paradigm from Israel’s precision-focused systems, emphasizing asymmetric capabilities designed to counter conventional military superiority through cost-effective force multipliers and unconventional deployment methods. This strategy reflects Iran’s resource constraints, technological limitations, and strategic doctrine of “forward defence” through proxy networks ( Lisman, 2021). Figure 2 illustrates the organizational structure of Iranian cyber and information operations, demonstrating integration of AI capabilities across multiple operational domains.

**Figure 2.**
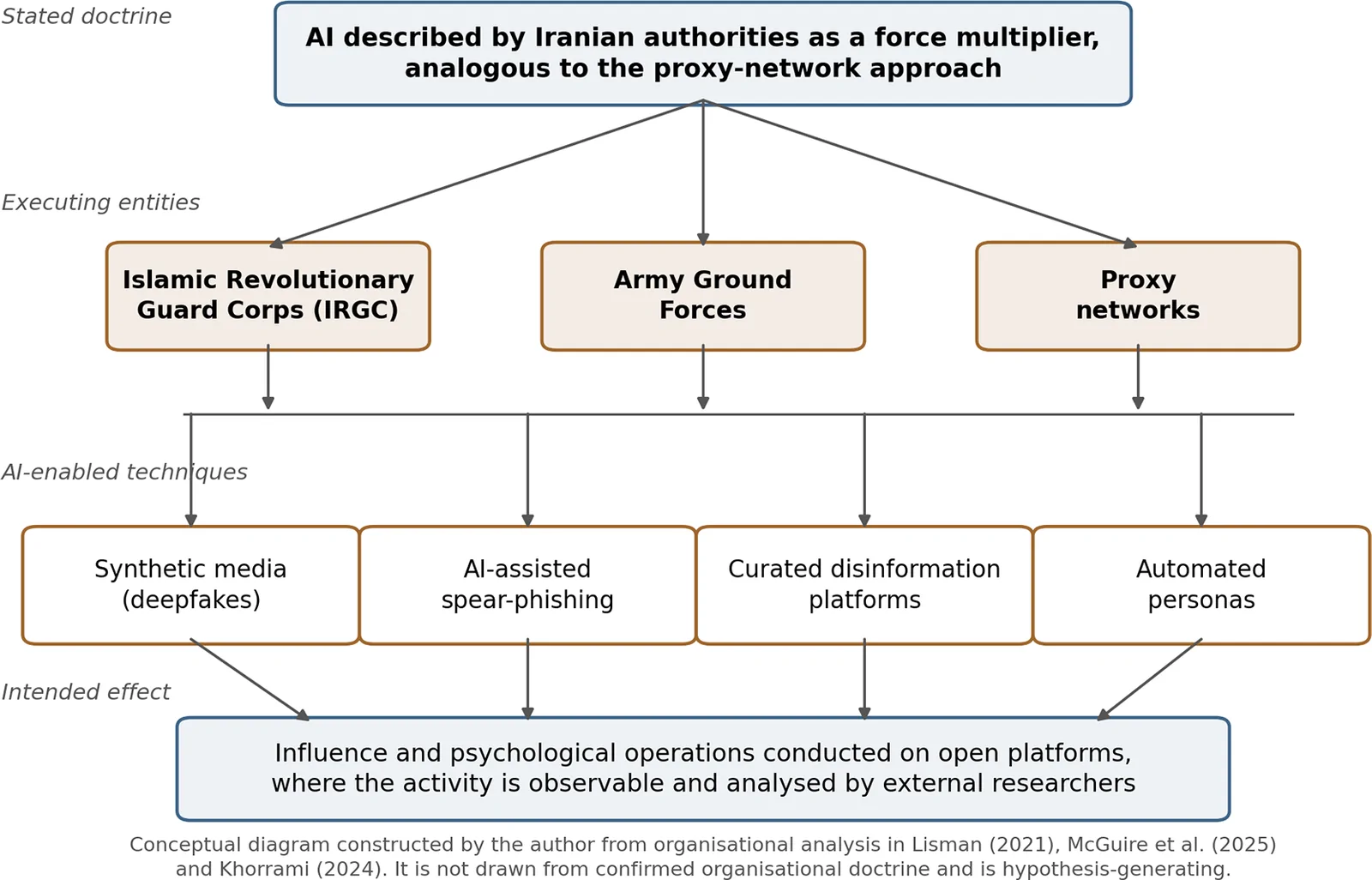
Iranian cyber and information operations structure. This is a conceptual diagram constructed by the author based on organizational analysis from Lisman (2021), McGuire et al. (2025), and Khorrami (2024).

Iranian military authorities explicitly describe AI as a force multiplier, analogous to their proxy network approach throughout the Middle East. Consistent with strategic analysis, Iran has mobilized significant numbers of highly educated computer engineers to pursue AI capabilities despite hardware and infrastructure limitations, concentrating on rapid deployment of basic autonomous capabilities rather than complex precision platforms ( Mieses & Noelle Kerr, 2024).

**4.2.2 Autonomous drone systems**

**Suicide drone technology:** Iran has deployed suicide drones utilizing artificial intelligence for autonomous target detection and engagement through image processing capabilities and pattern recognition algorithms. The Iranian Army Ground Forces and Islamic Revolutionary Guard Corps (IRGC) have identified these systems as primary components of Iran’s autonomous weapons arsenal.

**Swarm Intelligence:** Iran has developed autonomous drone swarms featuring mother platforms controlling multiple smaller suicide drones. This system represents Iranian advancement in distributed autonomous control and coordination, though capabilities remain incompletely verified through independent technical assessment. The swarm architecture enables coordinated attacks capable of overwhelming air defenses through numerical advantage and distributed attack vectors, complicating defensive responses ( Ramachandra, 2023). Table 5 summarizes the types of autonomous drone systems deployed by Iran, their AI integration levels, and operational status.

**Table 5.** Autonomous Drone systems.

System type	Capability	AI integration	Operational status
Autonomous Suicide Drones	Target Detection & Engagement	Image Processing AI	Operational
Drone Swarms	Coordinated Attack	Swarm Intelligence	Demonstrated
Mobin Cruise Missile	Autonomous Navigation	DSMAC Guidance	Operational
Ground Robots	Armed Reconnaissance	Autonomous Network	Development

Figure 3 sets out the documented attributes of these systems and the banded assessment for the category as a whole.

**Figure 3.**
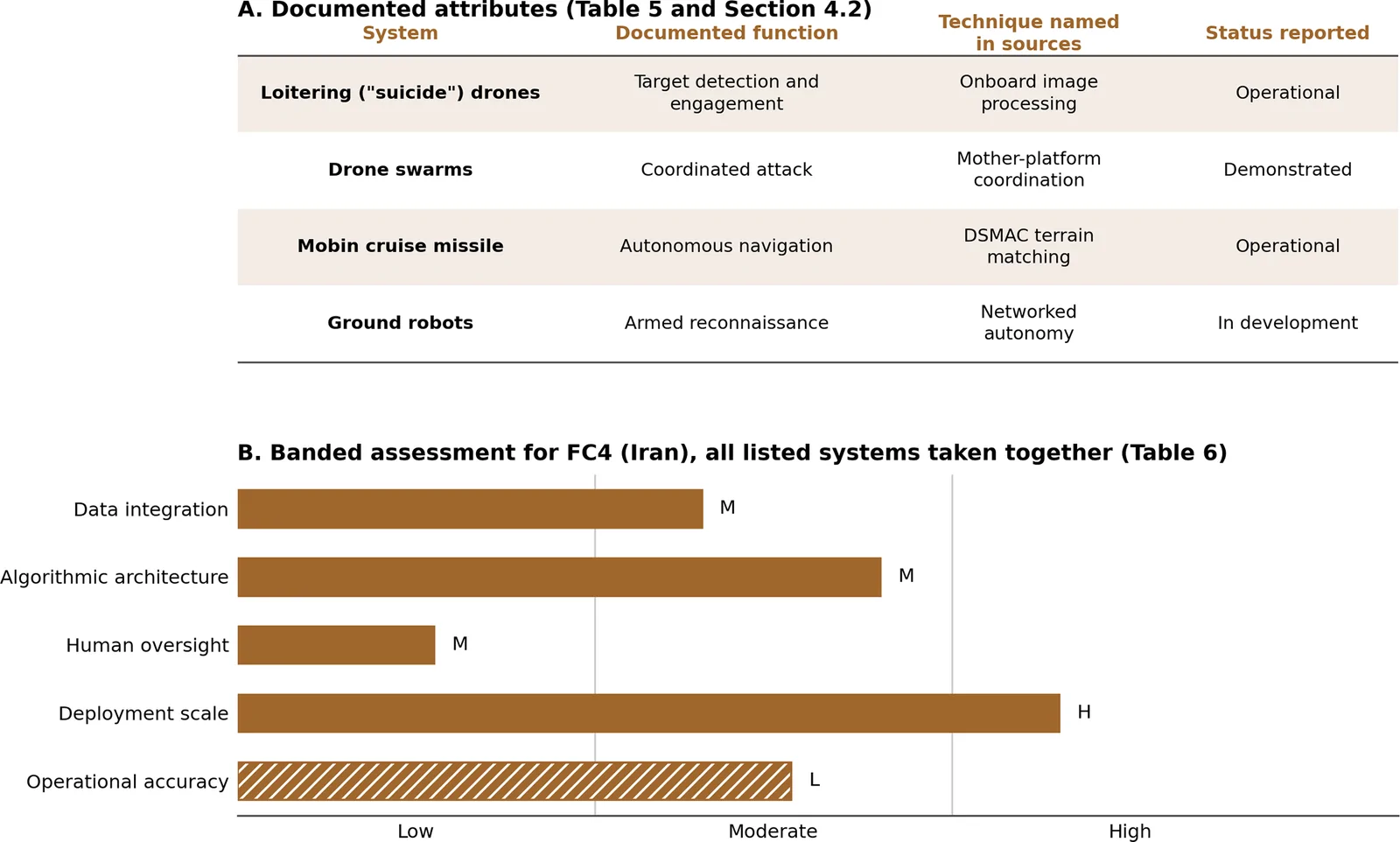
Iranian systems in functional category FC4: documented attributes and category-level banded assessment.

Panel A lists the documented function, the technique named in the cited sources, and the reported status of each system ( Table 5, Section 4.2). Panel B gives the banded assessment for the category as a whole, with evidentiary confidence ( Table 6); no per-system score is implied.

**4.2.3 AI-Enhanced missile systems**

The Mobin cruise missile system exemplifies Iran’s incorporation of artificial intelligence into long-range precision strike capabilities. This system utilizes Digital Scene Matching Area Correlation (DSMAC) guidance, an autonomous guidance technology (terrain matching rather than machine learning) initially developed in the 1980s and subsequently refined for modern applications. This advancement facilitates autonomous navigation to predetermined targets without relying on external guidance systems, providing resilience against electronic warfare and GPS denial ( Lisman, 2021). DSMAC technology enables the missile to compare real-time terrain imagery with stored reference images, making autonomous course corrections to reach designated targets. While representing relatively mature technology, DSMAC integration demonstrates Iran’s capacity to adapt existing autonomy technologies to overcome technological and resource constraints.

**4.2.4 Cyber warfare integration**

Iran has significantly integrated artificial intelligence into its cyber warfare capabilities, representing one of the most advanced applications of AI in its military arsenal. According to security analysis, Iran’s cyber operations exhibit “increasing sophistication and potentially more dangerous cyber capabilities” through AI integration ( Emegha & Okoli, 2025).

**AI-Enhanced cyber operations:** Iranian cyber capabilities encompass advanced digital techniques designed to influence, deceive, and manipulate information spaces:

• **Deepfake content creation:** Production of highly realistic synthetic media for propaganda and influence operations
• **Sophisticated phishing campaigns:** AI-powered targeting of strategically significant individuals and institutions with personalized social engineering attacks
• **Fake news platforms:** Establishment of AI-curated disinformation networks targeting specific population segments
• **AI-powered personas:** Deployment of autonomous virtual characters conducting psychological operations and shaping public opinion trends on social media platforms ( McGuire et al., 2025)

These capabilities represent significant force multiplication in information warfare, enabling Iran to project influence and conduct operations despite conventional military disadvantages.

### 4.3 Limitations and constraints of Iranian AI systems

Despite technological developments, Iranian military AI capabilities remain constrained by structural and technical factors limiting system sophistication and operational effectiveness:

**Hardware constraints:** International sanctions severely limit Iran’s access to advanced semiconductors and high-performance computing hardware, adversely affecting AI processing sophistication and precision. While advanced military systems employ specialized processors, Iranian systems typically utilize commercial-grade components, resulting in reduced operational efficiency. Sanctions have also resulted in absence of unified monitoring and sensing infrastructure, hindering provision of sufficient and diverse training data for AI applications. This negatively affects efficiency of Iranian AI-supported targeting systems and their adaptability to varied operational contexts ( Khorrami, 2024).

In contrast, Israeli systems benefit from extensive surveillance networks and sophisticated data environments, receiving technical support from leading technology companies, particularly in communications surveillance. These capabilities significantly enable collection of actionable intelligence on specific targets, enhancing effectiveness of AI-driven military operations (CSIS; RAND Corporation, n.d.).

**Integration issues:** Institutional fragmentation within Iranian military organisation—divided among Revolutionary Guard, regular army, and proxy forces—generates substantial challenges in AI system harmonization and data exchange across entities. This fragmentation inhibits systems’ capacity to operate in integrated, efficient manner, reducing overall operational effectiveness and limiting strategic coordination capabilities. A broader methodological limitation applies to both sides of the comparison: military AI systems are inherently classified, and the publicly available information represents only a fraction of actual technical capabilities and operational practices. The assessments in this study are necessarily limited to what has been revealed, leaked or independently documented. It should therefore be considered a comparative analysis of reported and documented capabilities, rather than of the totality of systems in use. This limitation is characteristic of all research on active military technologies and cannot be fully addressed through secondary data analysis alone.

### 4.4 Within-category comparison

Following Section 3.6, comparisons are made within functional categories. The five banded indicators and the evidentiary confidence ratings that were assessed are reported in Table 6; compliance was not banded, but rather classified in a qualitative manner (see Section 3.7), which is reported separately in Figure 6. The paragraphs below outline the roles that each category’s comparison plays and does not play. If there is no assessable system for one party, this is recorded as an evidentiary absence not as a finding of the absent capability.

The number of items indicated (inclusion criterion (iii) Section 3.8) is given as “Ind. assessable” but legal compliance is classified qualitatively and separately. Bands: L Low, M Moderate, H High, with within-band position (low/mid/up) added where appropriate to the rubric in Table 2 and not included where not. Evidence Confidence follows in parenthesis: (H) High, (M) Medium, (L) Low. IL = Israel, IR = Iran. Undetermined cells are not an assessment of low capability, but rather an evidentiary absence as marked in cells with the word “und.” Assessments are comparable down a column within a functional category only (Section 3.6). “Ind. assessable” counts, out of six (the five banded indicators plus the compliance classification), the indicators for which the evidence met inclusion criterion (iii); “6/5” gives the count for each of the two systems named. Reading example: “H up (H)” means High band, upper part of the band, High evidentiary confidence; “M (L)” means Moderate band, position not determinable, Low confidence.

The compliance classifications in Figure 6 are based on architecture and documented practice, rather than on outcomes. There are three points to make about reasoning. For Lavender and Besorah, there is scarcely any proportionality since the reported collateral damage parameters were applied by target class and not on a case-by-case basis, meaning that any judgment under Article 51(5)(b) was displaced, and there is also minimal precaution since the review period was of the order of seconds per nomination, which is hard to reconcile with the obligation under Article 57(2)(a)(i). In the case of Iron Dome, the key to the substantial classification is that the object acted upon is a munition and that control is exercised over the object prior to the action, that is, control before the fact. In the case of Iron Dome, substantial classification is predicated on the fact that the object acted upon is a munition and that the act of control is exercised over the object before it is acted upon, which is termed control before the fact. For Iranian FC4 systems the minimal classification is assigned a risk rating of Low (N/A) and it does not imply that the compliance architecture is weaker than that of the Israeli systems.

AI-assisted target generation 4.4.1 FC1. Israeli capability is recorded at high scale: high population-scale ingestion, and the reported generation of about 37,000 nominations is corroborated, while the model architecture and feature weighting and validation are not. There are two assessments that have increased importance. Operational accuracy is Moderate with Low confidence: the denominator (and hence the error rate) is not specified, and the error rate is about 10 per cent, which is at hierarchy level (e) and is not validated. Human oversight is Low to Medium, with Medium confidence, as short confirmatory review, seconds per nomination, is consistently attested but not officially recorded, but is assessed Low due to the lack of reconstruction of the system’s reasoning for an individual nomination. None of Iranian systems were included with the 1st criterion (iii). The only provable comparison is that Israel is capable and Iran is not. The more obvious asymmetry of the study is here, at an aggregate score of Iran being placed low in FC1, and there uncritical comparison would do the most to mislead.

4.4.2 FC2 — Automated defensive interception. The data integration and trajectory-prediction architecture of Iron Dome is described and documented, and the discrimination function used to decide whether an incoming projectile will hit a populated area (in which case, an interceptor is fired) is documented. The reported interception rates are high but have been disputed independently – both in terms of the rate as well as how it is calculated – and the assessment is banded accordingly. None of the Iranian systems qualified for criterion (iii). Interpretive rule: The Low human oversight assessment for Iron Dome is not a governance deficiency the same as the Low assessment for Lavender. Both have similar labels, but opposite content – the engagement window is seconds, the object engaged is a munition not a person, and automation is a part of the job and not a way to boost throughput. This is the most obvious case where a common score on a particular item would be misleading (Section 5.4).

Assisted fire control (4.4.3 FC3). Target lock and fire inhibition is described at specification level using computer vision. Here the word “accuracy” means “correct round placement, and no release while not in lock” and nothing else. Human oversight is rated High because the human has to choose the target, point the weapon and pull the trigger while the function of the system is to suppress the shot if it misses the target. No evaluable Iranian system. In FC3, the AI is most clearly enhancing human control over the system, as its authority is negative: the AI can stop a shot but can’t start one. This is an asymmetry between negative and positive algorithmic authority, which is generalized in Section 6.5.

Autonomous platforms and swarms (4.4.4 FC4). Goshawk is reported to be an autonomous counter-UAV interceptor system that is supervised at mission level but not engagement level, and whose non-destructive capture mode is legally relevant, but whose autonomy and operational parameters have not been independently studied. Different types of autonomy exist when it comes to loitering munitions, reported swarm architectures, and DSMAC-guided cruise missiles, and they are not the same. One common feature in their analysis is that in some configurations, post-launch intervention may be limited or unavailable. Without demonstrated abort capability, the human oversight is therefore judged as being a bounded, low-confidence risk with launch decisions based on target class, geography and time window. The limited and diverse FC4 sample showed much greater uniformity of control risks than the FC1–FC3 sample, and the evidentiary record, while not as well balanced as in that sample, seemed more balanced than in the prior samples. This research is a study of the relatively robust, but still limited, cross-case inference that is available with FC4.

4.4.5 FC5 — Cyber and information applications. Evidence exists for Iranian use of synthetic media generation, AI-assisted spear-phishing, automated persona operation and curated disinformation networks, where accuracy is defined as the ability to achieve effects, not necessarily to target the right audience, and is determined as Low confidence due to the contestation over effects-based measurement within information operations, even for the information operators. International human rights law are applied differently, as are the requirements of sovereignty and non-intervention, in comparison to the targeting rules of IHL. Israeli applications are well reported, but not to the extent of documented AI-specific functionality, and are Low confidence throughout. Unlike FC1, it is important to note the asymmetry in FC5, in that the Iranian record is far better documented as these operations are carried out on open platforms where they are observable and analysed by external researchers.

The “assessed capability by indicator, within functional categories” focuses on assessing the capabilities within functional categories, broken down by indicators. A new assessment format includes ordinal bands (Low/Moderate/High) instead of the 0–100 scale used in previous versions, with the evidentiary confidence rating as suggested by the name. Panels are functional categories as comparison is valid within one panel but not across panels (Section 3.6): human oversight refers to a different construct in each panel as does operational accuracy. If no system achieved the inclusion threshold in a given category, it is considered undetermined, which indicates that there was a clear absence of evidence, but not necessarily a finding of low capability. Ranking is based on author’s estimation; information comes from the supplementary data.

### 4.5 From system-level assessment to national-level patterns

Inference – not an absolute statement, but a set of tentative exploratory propositions – is required to move from findings on individual systems to findings on national approaches. Must be seen across systems in 2 or more functional categories, consistent with evidence of structural conditions where they are seen independently, and not contradicted by an assessed system without explanation. These circumstances do not ensure that the sample is representative of either state especially if there is no assessable counterpart in a category for one party. The residual risk that a characterization is at least partially an artefact of the systems that could be characterized is therefore significant and is not contrahed by the selection rule and the identified-but-unassessed systems set out in Section 3.8, nor by the sensitivity note that closes this section.

Proposal 1 is tentative: divergent optimisation targets. Israeli systems are optimised for data integration, reachback and precision, Iranian systems are optimised for cost, attrition tolerance and operation under denial in FC1-FC3, and FC4-FC5 are the same. The pattern crosses more than two categories, which corresponds to the independently documented structural conditions, in particular, access to advanced semiconductors under sanctions on one side and a large surveillance system and defence industrial integration on the other, and there are no assessed systems that contradict the pattern.

Proposal 2 is tentative, a risk to human control is proposed to be divergent. The documented problem with Israel is one of volume and review time, or producing nominations faster than one can interrogate them, in FC1. The problem with the Iranian system, documented in FC4, is that it’s an irreversible post-launch problem: once the system is launched, it can’t be recalled. These are various ways of failing the same requirement with evidence in multiple systems.

Proposal 3 (tentative): The structure of the institution could influence the integration of AI. The Iranian security structure is fractured, which means that data sharing and system harmonisation is limited, whereas the other side, the Israeli security structure is concentrated, which allows cross-domains fusion. This is as much based on structural evidence as on the system assessments, and is the most interpretive of the three.

Not supported 1 — relative overall capability. There is no support for any statement of the form “Israel’s military AI is more advanced than Iran’s”, as this would involve aggregating across categories that Section 3.6 forbids, and would be most heavily weighted on the category of FC1, which is the one with the highest evidentiary asymmetry.

Not supported 2 — relative legal compliance. There is no general statement that can be made regarding which state’s military AI is more IHL-compliant. The assessment here is for compliance to the architectural level at system level and the categories that each state is assessed best in are not the same, so this is not a transitive comparison.

Sensitivity. The FC1 finding is the most easily subject to revision. Proposition 1 would be substantially qualified but not propositions 2 or 3 if there is documentation of an algorithmic capability at the population-scale for prioritisation by the Iranian population. This study’s primary open evidentiary question is the FC1 asymmetry, not a finding.

## 5. Technical, ethical and legal considerations

### 5.1 Algorithmic foundations and technical architecture

The technical application of AI in military targeting systems relies on several core algorithmic approaches, each presenting distinct advantages and limitations in operational contexts. Analysis of documented systems reveals three primary technical paradigms deployed across both Israeli and Iranian implementations.

**5.1.1 Machine learning implementation strategies**

Both countries employ supervised learning algorithms that learn from historical engagement data and target characteristics, though the scope and sophistication of implementations differ substantially.

**Israeli systems (Lavender platform):** Israeli systems are reported to draw on multi-modal data sources, including visual reconnaissance, communication intercepts, and behavioural pattern analysis. System architecture enables real-time processing of diverse data streams, employing sophisticated correlation engines to provide accurate target identification and threat assessment. This multi-modal approach enables cross-validation of target identification across sensor types, increasing accuracy and reducing false positive rates.

Lavender is reported to apply machine-learning models to surveillance-derived data in order to score individuals on combatant-associated behavioural features; its model architecture, training data and validation are not publicly documented. The system’s reliance on correlation rather than causation, however, introduces risks of misidentification when civilians exhibit behavioural patterns statistically associated with combatants.

**Iranian systems:** Iranian applications focus primarily on computer vision-based target recognition and autonomous navigation. Image processing emphasis reflects reliance on open-source computer vision libraries and necessity of operating with limited sensor diversity. To overcome sensor limitations, Iranian systems have enhanced image processing algorithms and pattern recognition capabilities, optimised for specific operational environments where systems deploy.

Some Iranian drones are reported to use onboard image processing for target identification; no public source documents the model architecture or the training data used. The systems demonstrate adaptation to resource constraints through algorithmic efficiency prioritization over computational complexity.

**5.1.2 Neural network architecture and implementation**

The Iron Dome system illustrates automated real-time processing in defensive systems; public sources describe the processing capabilities essential for ballistic threat interception but do not document a neural-network implementation. Table 7 provides detailed comparative analysis of AI targeting architectures, contrasting Israeli and Iranian implementations across critical technical dimensions.

**Table 7.** Comparative analysis of AI targeting architectures: Israeli vs Iranian implementations.

Technical aspect	Israeli implementation as publicly described	Iranian implementation as publicly described
Processing Speed	Reported rapid processing for trajectory calculation; underlying architecture not publicly specified.	Reported resource-efficient processing; underlying architecture not publicly specified.
Data Processing	Public descriptions attribute radar-return processing; specific ML/CNN implementation not independently verified.	Reports describe robust signal or image processing in contested environments; specific implementation not independently verified.
Network Architecture	Specific neural-network depth and architecture are not publicly documented.	Specific network architecture is not publicly documented.
Training Data	Sources describe recorded engagements and simulation; dataset composition and validation are not publicly verified.	Sources describe simulation or synthetic-data supplementation; training data and validation are not publicly verified.

**Israeli neural network implementation:** Iron Dome performs rapid trajectory prediction from radar data; secondary sources attribute this to convolutional neural networks, but no primary source documents the implementation. The network architecture processes radar returns in real-time, predicting impact points and calculating optimal interception trajectories within milliseconds. Reported training on recorded engagements supplemented by simulation data is not publicly verified.

**Iranian neural network approaches:** Iranian neural network approaches: Secondary analysis assumes that Iranian systems use simpler architectures suited to limited computational resources; no primary source documents these architectures. It is plausible, though not documented, that training data limitations are addressed through synthetic data generation and transfer learning approaches, leveraging publicly available datasets and simulation environments to develop baseline capabilities subsequently refined through operational experience.

**5.1.3 Fuzzy logic systems and decision-making architecture**

Fuzzy-logic controllers can in principle support graduated rather than binary classification under incomplete or conflicting sensor data, the condition that obtains in urban environments where civilian and military presence is intermixed; such an architecture would assign degrees of membership rather than definitive categories and could flag ambiguous cases for heightened human scrutiny. No source examined in this study, however, documents a fuzzy-logic engine in any Israeli or Iranian system in the assessed set. The technique is therefore discussed here as an architectural option relevant to the control problem this study examines, and no capability assessment in Section 4.4 rests on its presence.

### 5.2 Legal and ethical framework: applied constraints

As autonomy in weapon systems increases, the temporal and spatial distance between human operators and selected targets expands, effectively delegating life-and-death decisions to algorithms. This raises fundamental ethical questions about human responsibility in the use of lethal force and the moral dimensions of algorithmic warfare ( Mauri, 2022).

**Distinction principle:** International Humanitarian Law requires clear distinction between combatants and civilians. AI systems must not undermine commanders’ capacity to make required legal determinations regarding target legitimacy. The AI-enhanced Lavender system sparked controversy due to reports that some military leaders treated civilian deaths as acceptable statistics rather than requiring case-by-case proportionality assessments.

According to reports, operational protocols permitted killing up to 100 civilians in bombing raids targeting senior Hamas or Islamic Jihad officials. The system was designed to conduct attacks when targets were inside homes at night, increasing likelihood of successful engagement but substantially increasing risks of killing family members and civilian neighbors.( G. Pascual, 2024). This approach raises serious questions about compliance with proportionality requirements under International Humanitarian Law.

A prominent example is the 2025 killing of journalist Anas al-Sharif in his tent. The Israeli army accused him of Hamas affiliation when bombing his shelter, killing five other journalists in the strike. This incident exemplifies concerns regarding protection of civilians and proper application of distinction principles in AI-assisted targeting ( Mahase, 2025).

**Proportionality principle:** International Humanitarian Law requires that anticipated civilian harm not be excessive relative to concrete and direct military advantage expected from an attack. AI systems must enable commanders to make proportionality assessments, yet compressed decision timelines and high target generation rates may undermine meaningful proportionality analysis. When human operators spend only 20 seconds verifying targets, as reported for Lavender system operations, the capacity for meaningful proportionality assessment becomes questionable.

**Precautionary principle:** Parties to conflict must take all feasible precautions to minimize civilian harm. This includes verification that targets are military objectives, selection of means and methods minimizing civilian harm, and provision of advance warning when circumstances permit. AI systems that prioritise operational efficiency over verification thoroughness may conflict with precautionary obligations, particularly when strikes are conducted in civilian-dense environments or against targets in family residences.

### 5.3 Accountability and legal responsibility

**Command responsibility:** Military commanders remain legally accountable for targeting decisions regardless of AI system involvement. However, the complexity and opacity of AI algorithms may prevent commanders from fully comprehending and validating system recommendations, potentially undermining effective command and control. When AI systems generate targets at rates exceeding human capacity for thorough review, command responsibility becomes difficult to exercise meaningfully.

**Algorithmic transparency:** The “black box” problem of many machine learning systems creates accountability and legal scrutiny challenges. Targeting systems must be capable of legal review consistent with Article 36 of Additional Protocol I to the Geneva Conventions, which requires legal review of new weapons, means, and methods of warfare. When algorithms cannot explain their targeting rationale in human-interpretable terms, such legal review becomes problematic. The relevant international legal and ethical frameworks governing AI-based weapon systems, including compliance requirements, are set out in this section, with per-system classification given in Figure 6 for military applications.

State responsibility and personal criminal responsibility. These two paths differ in the amount of evidence they need, and in how they fail when running with an algorithm. In order to establish individual criminal responsibility, the mental element must be proved as being a part of an identifiable person, which was precisely what is doubted in the case of a reviewer under nominal authorisation, who ratified but did not decide. State responsibility is triggered by the actions of organs of the state, not by the presence of an individual fault; it is engaged by the employment of a system which is inconsistent with IHL, irrespective of the determination of any individual’s fault ( Crootof, 2016).

**Article 36 review and its limits:** As currently practised, review examines the system. Under algorithmic conditions this is insufficient in three respects: a machine learning system’s behaviour is a function of its training data and deployed model version, so review at adoption does not govern the system after retraining; the properties determining whether meaningful human control is preserved — throughput, review time, operational envelope — are properties of employment rather than of the artefact, and fall outside conventional review scope; and review outcomes are rarely published, so the process generates no external assurance. Mechanisms C1–C2 in Section 6.3.1 address all three.

**Criminal liability framework:** Under international criminal law, individual criminal responsibility requires proof that an identifiable person committed, ordered or assisted a crime with the required mental element (Rome Statute, 1998, Arts. 25 and 30), and commanders may be responsible under the conditions of superior responsibility (Rome Statute, 1998, Art. 28). Whether a programmer’s design choices could meet these standards is unsettled and untested in practice; the opacity or autonomy of a system does not by itself establish anyone’s criminal responsibility. However, proving intent and establishing causal links between algorithmic decisions and unlawful outcomes presents significant evidentiary challenges. The distributed nature of AI system development—involving multiple programmers, data scientists, and military planners—further complicates attribution of individual criminal responsibility.

### 5.4 Meaningful human control under algorithmic scale and compressed decision time

This section further explores the study’s key conceptual contribution, that is, what is being controlled by humans, at what point does human control become irrelevant, and where does responsibility transfer as a result of that?

**5.4.1 Three elements of meaningful human control**

The literature consensus is that it is not enough for the human to be included in or on the decision loop in order to have meaningful control ( Roff & Moyes, 2015; Ekelhof, 2019; Amoroso & Tamburrini, 2020). This study follows Santoni de Sio and van den Hoven (2018) and the ICRC (2019, 2021) in viewing it as having three separate parts: understanding, enough situational and system knowledge to evaluate what the system has concluded, on what basis, and with what characteristic failure modes; time, enough of it to exercise that understanding deliberately rather than to ratify a pre-formed output; and capacity to intervene, a genuine, operationally as well as technically available ability to withhold, modify or abort. Separating them is important because they can be degraded in various ways, and need various remedies; understanding is degraded by opacity, time by volume and tempo, and capacity to intervene by irreversibility. One instrument that targets only one will not provide effective human control.

**5.4.2 Volume–time ratio as a possible structural constraint**

The core argument of this research is that the number of engagement options a system can generate and the time needed for the human to investigate each one could be a key structural limitation for meaningful human control in AI-assisted targeting. There is available evidence that supports this but no universal threshold or causal priority over other factors including: rules of engagement, command pressure, doctrine, staffing or opacity. The reported Israeli practice in FC1 is the most clearly reported instance in the material examined.

The capacity for reviewing a targeting cell is finite and, over a given time period, roughly constant: it is limited by the number of reviewers who are trained to review the cell, the amount of time they can be expected to work, and the amount of cognitive time needed to reconstruct the basis of a nomination. Algorithmic generation capacity does not have such restrictions; it grows with compute and data. If the number of individuals nominating for review exceeds the number that can be reviewed, one of three things must happen: nominations are not acted on, thus losing the benefit of the review (an institutional disadvantage); the nominalisation rate decreases further; or the review is transferred downwards and/or formalised as a checklist. The latter two affect the time dimension but preserve the formal structure of the human authorisation.

The result is what is called nominal authorisation, as each engagement is formally authorised by a human, the audit trail documents a human decision at each node, but no humans have exercised the deliberative judgement that the authorisation is supposed to embody. Nominal authorisation is also legally important as it meets the procedural indicia of compliance without meeting the substantive requirement which the procedure evidences, and, for that reason, the process is hard to see from the outside of the organisation, which is a crucial aspect of the design of verification mechanisms (Section 6.3.1). The reported use of the IDF in FC1 is the clearest documented example that was found in the material examined, and it is described here as an instance of a structural condition, rather than a failure peculiar to a particular military: The fact that the argument above suggests that the same pressure applies to all militarys with high throughput generation capability, regardless of their doctrine or intent, indicates that the above is a structural condition rather than a failure idiosyncratic to them.

Table 8 provides, for each category, the decision tempo, the form of human control that is feasible, the most vulnerable component and the residual responsibility gap.

Three observations follow. *Automation is not uniformly a control problem.* In FC2 it is a consequence of the physics of the threat; requiring per-engagement deliberation would not increase civilian protection. The appropriate locus of control is the **deployment envelope** — siting, engagement authorisation, firing rules — exercised in advance rather than in the moment, which is genuine control. *The most acute problems are not in the most autonomous systems.* FC1 systems are formally the least autonomous in the set, yet exhibit the most severe documented degradation, because volume and tempo hollow out an authorisation that remains formally intact; FC3 systems exercise autonomous authority over the release of a round yet strengthen control, because that authority is purely negative. Autonomy as conventionally defined is therefore a poor predictor of where control problems arise. *Irreversibility and volume require different remedies.* FC4’s problem is that intervention capacity expires at launch — more review time before launch does not solve it, and the remedy lies in abort capability and encoded engagement limits. FC1’s problem is that review time collapses under volume — abort capability does not solve it, and the remedy lies in throughput limits and reviewability requirements.

**5.4.3 Migration of responsibility**

Where meaningful human control is attenuated, responsibility does not disappear; it migrates, and it does so differently by category. Sharkey (2012), Sparrow (2007) and Crootof (2016) frame the responsibility gap as arising where no human is sufficiently proximate to bear individual criminal responsibility. In FC1 responsibility migrates upward and backward, from a reviewer who under nominal authorisation cannot meaningfully be said to have decided, to those who set throughput expectations, collateral damage parameters and review protocols; command responsibility doctrine can reach these actors in principle, but requires evidence about tempo settings and review protocols that is precisely what is not disclosed. In FC4 it migrates forward from the moment of commitment: the legally decisive act is the launch decision and its encoded parameters, not the terminal engagement. In FC2 the gap is narrow, resting with those who set the envelope. In FC5 it is widest, because attribution is contested, the applicable framework unsettled, and the harms diffuse. The governance implication is direct: accountability mechanisms designed for the moment of engagement will systematically fail to reach the decisions that determine outcomes — a limitation Article 36 review shares, since it examines the system rather than its employment tempo.

**5.4.4 Reviewability rather than explainability**

For the purposes of meaningful human control, the transparency requirement is better specified as reviewability: the ability of a human, within the operational time available, to reconstruct the basis of a specific recommendation sufficiently to accept or reject it, and the ability of an investigator afterwards to reconstruct what the system concluded and what the human did with it ( Holland Michel, 2020). Reviewability is weaker than full model interpretability and therefore achievable, and stronger than the presence of a log, because it specifies what the log must be sufficient to reconstruct. So framed, it is specifiable in procurement, testable in Article 36 review and verifiable after the fact — properties “explainability” as usually formulated does not possess.

**5.4.5 The four constructs separated in the present cases**

Throughout this study four constructs are examined separately, and are not merged into a larger concept of the term “effectiveness”. Technical accuracy is the correctness of the main output of a system according to the definition given for its functional category. Operational effectiveness is the accomplishment of the desired military outcome. Compliance with the law means that distinction, proportionality, precaution and reviewability requirements were met in design and in use. Humanitarian outcome – is the true impact on civilians; for protected persons and objects. None of the 4 sets up any of the others. Civilian casualties are not in themselves an evidence of algorithmic inaccuracy — an attack could kill civilians, but the target was correctly identified, due to proportionality considerations, the choice of munition, the timing, the intelligence on civilian presence, or command level rules of engagement. — nor an inaccuracy of an algorithm could lead to the loss of civilians, given that the target was misidentified. On the other hand, a technically correct identification meets one requirement for distinction and does not address the question of whether there were incidentally harmful effects that were unwarranted compared to the anticipated military advantage, nor address the question of whether any practicable precautions were taken. The separation is applied to material for this section.

In relation to error rate. About 10 per cent is a loosely sound technical guess on accuracy, has no denominator and has not been independently substantiated. Multiplying the reported quantity of nominations to estimate the number of misidentified persons as earlier versions of this manuscript did is a compounding of three errors: first, that a Low-confidence figure represents a measurement; second, that all nominations led to an engagement; and third, that—from technical to humanitarian outcome—there was no warrant to move from the former to the latter. The calculation is canceled out. It can only be said that a system running at the stated size and at an unvalidated error rate and compressed review raises a structurally higher risk of mis-identification which cannot be determined from open sources.

On accuracy of claims and humanitarian results. The evidence available does not allow a clear distinction to be made between any single cause and the difference between the precision that has been claimed and civilian harm reported. There were at least five non-exclusive ways in which it could be produced: (i) technical misidentifications; (ii) correct identification followed by a proportionality judgement that allowed for significant incidental harm; (iii) correct identification and lawful proportionality judgement, but then other choices of munition, timing or method that increased harm; (iv) degradation of review under volume, so neither (i) nor (ii) was meaningfully reviewed; and (v) rules of engagement and collateral damage parameters set at command or political level. Open-source evidence shows that there is a gap, but does not differentiate between these. Reported protocols allowing high collateral damage thresholds to be met for senior targets, and the reported practice of targeting at night in residential areas, are evidence of human decision making, not of algorithmic accuracy, and so bear on (ii), (iii) and (v).

On effectiveness. The term effective operation of a system as used in this study implies that the system is effective in achieving its intended effect; no implication is intended that a system is “compliant”. The high effectiveness that was assessed for Iron Dome has nothing to do with the lawfulness of any operation it allows for, nor has the recorded scale of use of Iranian loiter munitions anything to do with their lawfulness. On the other hand, compliance issues mentioned in this report are about the quality of compliance architecture, not whether any specific attack is legal or not legal.

## 6. Regional security implications and strategic assessment

### 6.1 Strategic stability and escalation dynamics

Deployment of AI-powered targeting systems in the Iranian-Israeli context introduces novel escalation dynamics that differ significantly from conventional approaches to strategic stability. The automation of target generation and engagement compresses decision-making timelines, potentially reducing opportunities for diplomatic intervention and crisis management. When AI systems identify and prioritise targets within minutes or hours rather than days, windows for de-escalation narrow substantially.

The speed advantage provided by AI systems creates incentives for preemptive action during crises, as states may perceive advantages in striking before adversaries fully mobilize AI-enhanced capabilities. This dynamic introduces crisis instability, where both parties face pressures to act quickly rather than pursue diplomatic resolution. The proliferation of AI military systems without accompanying arms control frameworks risks accelerating regional arms races and increasing conflict probability.

### 6.2 Humanitarian impact assessment

Civilian casualties have been reported in Gaza, including children, and medical and press personnel, as well as repeated attacks on civilian infrastructure, such as water-distribution facilities, residential buildings, and in areas where people live ( Mahase, 2025). Reports are verified in areas of active conflict. They do not offer a counterfactual with which to evaluate the performance of an algorithm, rather they support investigation of whether civilian harm has been reduced due to claims of precision, target-selection procedures, weapon choice, review practices, or command policies. The defensible statement is therefore that the reported magnitude of civilian harm.

### 6.3 Strategic recommendations

**6.3.1 Specific governance mechanisms**

Each mechanism is specified by the actor involved, the instrument by which it would be implemented, the indicator by which compliance could be assessed, and its near-term feasibility. Mechanisms are proposed solutions to the deficits found in Section 5.4 and are not a form of treaty obligation. They discuss the points where control can be given or taken: system design, employment tempo, legal review, and international coordination. The complete set is presented in Table 9.

Table 9 sets out all seventeen mechanisms, grouped by the point at which control can be exercised. Each mechanism answers a failure mode identified in Section 5.4. Volume–time saturation in high-throughput target generation is addressed by throughput ceilings and minimum deliberation time (A2, A3), organisational separation of nomination from strike authorisation (B1), and automatic escalation when throughput exceeds review capacity (B4). Loss of intervention capacity at commitment in post-launch systems is addressed by abort or recall capability and encoded engagement limits (A4, A5). The inability to reconstruct the basis of an individual engagement is addressed by a reviewability specification (A1), recording of collateral damage estimation inputs (B3), and preservation of targeting logs and review timestamps (C4). Drift between the system that was reviewed and the system that is deployed is addressed by extending Article 36 review to employment tempo and throughput (C1) and by re-review on model update or retraining (C2). Reviewer certification (B2), post-incident investigation access (C3) and the international measures (D1–D4) apply across failure modes. The feasibility ratings in Table 9 are the author’s assessments of near-term practicability, not empirical findings.

There are three noteworthy features. Mechanisms D1 and D2 do not duplicate the multilateral work already underway (namely the Guiding Principles affirmed by the CCW Group of Governmental Experts, 2019, and the reporting process opened with the United Nations General Assembly Resolution 78/241, 2023) but instead build on it; D3 reaffirms, in prohibitory terms, a concern already expressed in the ICRC’s position on autonomous weapon systems ( ICRC, 2021). The most feasible mechanisms are national and unilateral (A1–A3, B2–B3, C2): they do not involve an international agreement, can be adopted by a single defence ministry, and would have a material effect on the reviewability of targeting decisions, which is relevant because the multilateral track has been slow to develop and single unilateral adoption by capable states would create the emerging norm ( Bode & Huelss, 2018). The set is weighted towards tempo and throughput, not autonomy per se, following Section 5.4, and is a departure from the usual regulatory emphasis on degree of machine autonomy, which is not the appropriate variable, the present analysis indicates. The only mechanism evaluated as being low in near-term feasibility but meeting the criteria for inclusion is D3 - a ban on autonomous targeting of individuals on the basis of pattern of life classification established by algorithms alone, which is an attack against the practice identified here as least reconcilable with distinction.

### 6.4 Limitations of the study 

Several important limitations apply to this study that should be considered in interpreting its findings.

Data Access and Verification: Information on military AI systems in active conflict zones is inherently constrained by restricted field access, the classification of operational details, the prevalence of propaganda, and the lack of robust ground-truth verification. A large portion of the technical parameters and performance figures quoted in this study are based on self-reported military data, investigative journalism or OSINT platforms and not independently verified sources.

Conflict-zone data specifically. Three problems arise beyond mere source unreliability: casualty and damage data are collected by institutions under attack, which tend to undercount when damage is likely to be greatest; performance claims from parties with an interest in deterrence signalling tend to overstate performance; and documentation depends on leak and capture or investigative access, which are none randomly distributed, so absence of evidence is not evidence of absence — treated analytically in Section 3.8, but merely disclosed here.

Methodological limitations: This research is limited to secondary data analysis only, no primary data collection, field interviews or direct technical access to military systems were conducted. All evaluations were performed by one researcher which may incorporate some subjective bias in the scoring framework despite using the rubric in Table 2. A limitation acknowledged is the lack of inter-rater reliability testing.

Asymmetry in evidence: The empirical material available on Israeli systems is considerably more detailed than that available for Iranian systems. This is a function of differences in media transparency, investigative access, and public documentation, not of differences in analytical effort. This asymmetry means that the comparative analysis is inherently unequal in evidentiary depth, and assessments of the Iranian system should be interpreted as emerging indications rather than fully documented case studies.

Scope limitations: This study is limited to two states in one region and cannot claim generalizability to military AI applications in other contexts. Due to the fast pace of AI technology development, the capabilities of the system described here may have changed since the time of analysis.

### 6.5 Contribution beyond the two cases

A two-case comparison is descriptive unless it yields propositions that travel. Three are advanced, each stated so as to be testable elsewhere.

The focus is on the volume–time ratio rather than autonomy as the primary determinant of the maintenance of meaningful human control. In the event that the number of algorithmic engagement options generated is higher than a human can review, despite a robust authorisation structure in place, with no autonomous system in the traditional sense. In contrast, a system with high degree of autonomous control may maintain or enhance control when control is negative. The proposition suggests that such control issues are not centralized in the often discussed autonomous weapons, but are more likely to be found in high throughput decision support systems, and it can be tested anywhere throughput and the time taken to review the data is available.

Proposition 2: Resource-poor and resource-rich militaries approach the same governance issue from different angles. One case is one of degradation (volume, compressed review), the other is one of post-launch irreversibility (low-cost, attrition-tolerant systems). Both pathways don’t require complex algorithms. The implication for proliferation policy is clear: there won’t be a second pathway because the systems that implement the third pathway are cheap, have mature techniques and are widely available as is.

Proposition 3: There is evidentiary asymmetry that skews a comparative ability assessment in determinate direction. Documentation relies on leak, capture, and investigative access; these are more frequently available in open media environments and in domains where systems operate in the open, thus systematically over-documenting the capabilities and the shortcomings of transparent actors compared to opaque ones. This mechanism is consistent with the FC5 inversion. The methodological implication is that the assessment of military AI should be reported as a first-class result; the same is also true of this study.

These two together rephrase the contribution of the comparison. The fact that Israel focuses on complex, integrated capabilities and Iran focuses on cheap, asymmetric and distributed capabilities is descriptive and not all that surprising. The analytical value of the two pathways is that it is the conditions in which human control over the use of force is failing that are structural rather than technological; it is the conditions on both ends of the capability spectrum, and it is the regulatory variable that is most likely to be effective, namely, decision tempo and throughput, that current governance debates have focused on.

## 7. Conclusions

This study analysed two kinds of military systems claimed to be developed by Israel and Iran, treating them as case units, identified systems by their functions and compared them before the analysis, and determined the level of the evidence behind the individual systems and built them explanatory confidence ratings on six indicators under four dimensions.

On the systems. The differences between the two national sets are not so marked as the aggregate comparison might indicate within categories. In the case of target generation, the Israeli capability is recorded at a high scale, and Iranian capability is undetermined — clearly an evidence finding, not a capability finding. High automation is a consequence of the physics of the threat in defensive interception, and is not a control deficiency. AI is reinforcing human control in assisted fire control, the AI control is negative. The evidentiary record for them is most balanced and the control problem is most acute for both of these platforms, as of now. The ability sets are closest, the evidentiary record most balanced and the control problem is most acute for both of these platforms as of now. In cyber and information applications the asymmetry inverts.

On human control. There are two fundamentally different ways meaningfully human control can fail: in the case of algorithmic volume, the review time can be rendered negligible, leaving behind nominal authorisation, in which all engagements are formally approved and no meaningful decisions are made; in the case of post-launch autonomous systems, the space for intervention capacity can be exhausted at the moment of commitment. The two documented cases illustrate these two, respectively, establishing that they are attributes of system class and employment pattern, and not of national doctrine.

On the law and accountability. There is no substantive change in the rules applying to the use of AI, but there can be a change in the way they are applied: the concept of a substantive rule for the review in Article 36 for the artefacts; the concept of a mental element for individual criminal responsibility is masked by the use of nominal authorisation; the procedural indicia of compliance remain unaffected where the substantive requirement is not met. Accountability is therefore stronger in the state responsibility route and the record preservation/reviewability route.

On outcomes. There is a clear separation between technical accuracy, operational effectiveness, legal compliance and humanitarian outcome. However, civilian casualties do not in themselves constitute algorithmic error, and technically correct identification does not constitute at the same time an attack being proportionate or lawful. The reported damage to civilians in Gaza, together with evidence for high-volume generation under compressed review, should lead to an independent investigation of the targeting process as a whole, not to any specific element on open-source evidence.

On governance. There are 17 mechanisms that are specified with responsible actors, instruments and verification indicators. The most feasible are national and unilateral, with a focus on regulating decision tempo, throughput and reviewability.

In addition to the two cases. The failure of meaningful human control is not one result of one type of military or another, but one result for both types of military, and even in both cases, it is the governance variable least regulated that is most likely to matter. The current evidence suggests that the relevant practice is being constituted operationally – that is, in the midst of conflict – and not deliberatively. The threat is not that military AI will not be developed, which is impossible, but that the number of decisions that can be made per second by the system is not more efficient than that of humans.

## Data Availability

The data underlying the assessments in this article have been deposited in the Zenodo repository and are publicly accessible at: https://doi.org/10.5281/zenodo.17385265 ( Faris, 2025). The deposit makes the coding and per-indicator decisions auditable; it does not itself establish the underlying source claims, which rest on the cited primary and secondary sources. The repository contains:
• Structured data extraction tables documenting technical specifications, operational capabilities, and performance metrics of AI targeting systems deployed by Israeli and Iranian military forces • Per-system, per-indicator assessment matrices recording the ordinal capability band (Low, Moderate, High) and within-band position for each of the six indicators grouped under four dimensions, together with the evidentiary confidence rating assigned to each assessment • Qualitative legal-compliance classifications (substantial, partial, minimal, indeterminate) for each assessed system against distinction, proportionality, precaution and reviewability, with the supporting evidence for each classification • Complete source documentation index with reliability assessments and citation trail • Methodological codebooks explaining the band rubric, the within-band placement rules, the evidentiary-confidence criteria, and the mapping from the earlier numerical inputs to the revised ordinal bands All supplementary data are released under a CC-BY 4.0 license. The following figures and tables in this article are directly derived from the Zenodo supplementary dataset. Table 2 (capability band definitions), Table 6 (assessed system set and banded indicator assessments), Figure 4 (assessed capability by indicator), Figure 5 (operational employment and assessed accuracy), and Figure 6 (qualitative legal compliance classification) are derived from the banded assessments and their documented inputs. As their captions note, Figures 2 and 3 are conceptual diagrams drawn by the author. This study employs a secondary data analysis approach, systematically reviewing and synthesizing publicly available information from peer-reviewed academic journals, verified investigative journalism, open-source intelligence platforms, technical documentation, and international legal frameworks. No primary data collection, experimental research, or work with human participants was conducted. All source materials are comprehensively cited in the manuscript’s References section and remain accessible through their original publishers. For data-related queries, contact Den.lamia.faris@uoanbar.edu.iq.
