# Supplementary material for: Algorithmic Targeting in the Iranian–Israeli Confrontation: Technical Realities, Legal thresholds, and the Boundaries of Human Control
Source: F1000Res. 2026 Oct 9;14:1200. Originally published 2025 Nov 3. [Version 4] doi: 10.12688/f1000research.169794.4 (PMC13654070; doi:10.12688/f1000research.169794.4)
Supplement: Peer review history

# Reviewer report: Reviewer response for version 4

**DOI:** 10.5256/f1000research.211354.r528512

**Published:** 2026-10-10

**Authors:** Burak Oktenli

**Affiliations:** 1 Georgetown University, Washington, DC, USA

**Updated Review of Version 4**

 I have reviewed Version 4 together with the author’s point-by-point response to my previous report. The authors have addressed the six major concerns I raised in Version 3 in a substantive and satisfactory manner.

 In particular, the numerical scoring of legal compliance has been replaced by a qualitative classification with explicit inferential limits; the unsupported extrapolation from the reported error rate to an estimated number of misidentified individuals has been removed; evidentiary confidence is now attached to each assessment and absence of evidence is distinguished from low capability; the source-claim mismatch in the analytical framework has been corrected; Article 36 review has been clearly separated from the concept of meaningful human control; and the identified bibliographic and source-traceability problems have been corrected.

 The revised functional taxonomy, within-category comparison logic, ordinal capability bands, and explicit separation of technical accuracy, operational effectiveness, legal compliance, and humanitarian outcome also materially improve the analytical coherence of the paper.

 I have only two minor residual comments. First, in Section 4.4.1 the sentence stating that “the only provable comparison is that Israel is capable and Iran is not” appears inconsistent with the revised evidentiary rule that absence of assessable public evidence should be recorded as undetermined rather than as evidence of absent capability. I suggest revising this sentence to state that Israeli FC1 capability is documented in the assessed public record, whereas no Iranian FC1 system met the evidentiary inclusion threshold. Second, the final sentence of the conclusion concerning the number of decisions per second is difficult to interpret and would benefit from rephrasing around the paper’s clearer volume–time argument.

 These are minor editorial corrections and do not affect my overall assessment.

**Updated approval status: Approved.**

Is the work clearly and accurately presented and does it cite the current literature?

Partly

If applicable, is the statistical analysis and its interpretation appropriate?

Not applicable

Are all the source data underlying the results available to ensure full reproducibility?

Partly

Is the study design appropriate and is the work technically sound?

Partly

Are the conclusions drawn adequately supported by the results?

Partly

Are sufficient details of methods and analysis provided to allow replication by others?

Partly

Reviewer Expertise:

NA

I confirm that I have read this submission and believe that I have an appropriate level of expertise to confirm that it is of an acceptable scientific standard.

# Reviewer report: Reviewer response for version 3

**DOI:** 10.5256/f1000research.202374.r507419

**Published:** 2026-08-11

**Authors:** Burak Oktenli

**Affiliations:** 1 Georgetown University, Washington, DC, USA

**Overall Assessment:**

 The manuscript investigates the integration of AI into military targeting systems in the context of the Iranian-Israeli confrontation. It provides a comparative analysis of the systems’ technical architectures, operational deployment, and compliance with international humanitarian law (IHL). Through a mixed-method approach and a six-dimensional analytical framework, the author seeks to bridge the gap between technical system capabilities and legal accountability thresholds.

 The paper has clear academic merit, particularly in its structured effort to contrast Israel’s precision-focused, data-intensive approach, exemplified by systems such as Lavender, Besorah, and Iron Dome, with Iran’s asymmetric, cost-conscious, and swarm-oriented approach, including autonomous drones and cyber operations. The revisions introduced in Version 3, particularly the source-reliability classification and the explicit acknowledgement that the 0-100 scores represent analytical assessment rather than statistically validated measurements, demonstrate meaningful responsiveness to the previous peer-review comments.

 However, certain methodological vulnerabilities and bibliographic inaccuracies will need to be corrected before the article can fully meet the approval criteria. The principal concerns relate to the conceptual validity of assigning numerical scores to qualitative legal forms, the reliance on extrapolated journalistic metrics in the humanitarian-impact analysis, the substantial evidentiary asymmetry between the two national case studies, and important source-claim mismatches within the theoretical framework. Addressing these issues would significantly strengthen the study’s analytical rigour and transparency.

**MAJOR COMMENTS**

**Major Comment 1: Conceptual Limitations of Scoring Legal Compliance**

**Location: **Section 3.3, “Analytical Framework,” and Figure 4, “International Humanitarian Law Compliance Assessment”

**Issue: **The manuscript assigns quantitative scores on a 0-100 scale to abstract legal concepts such as “Distinction Capability” and “Proportionality Assessment.”

**Why this matters: **IHL principles are inherently contextual, qualitative, and dependent on the circumstances of individual attacks. Assigning linear numerical values to compliance with principles associated with Articles 51 and 57 creates an appearance of precision and methodological validity that the available evidence cannot support. Although the manuscript states that the scores are qualitative analytical assessments, presenting them in a numerical bar chart may still convey statistical certainty and comparability.

 fpu

**Required revision: **Reframe the IHL compliance assessment as a qualitative analytical matrix in place of the 0-100 scoring exercise. The author should explain how particular operational architectures, procedures, or documented practices support or fail to support the relevant legal requirements without reducing legal compliance to a numerical value.

**Evidence needed for closure: **A revised Figure 4 and corresponding text replacing the numerical scale with a qualitative classification framework, such as substantial, partial, minimal, or indeterminate compliance, supported by clear narrative justification.

**Major Comment 2: Reliance on Extrapolated Metrics in the Humanitarian-Impact Analysis**

**Location: **Section 4.1.1, “Lavender System,” and Section 6.2, “Humanitarian Impact Assessment”

**Issue: **The manuscript extrapolates a hypothetical figure of 3,700 misidentified individuals by applying a reported 10% error rate to a list of 37,000 potential targets derived from journalistic sources.

**Why this matters: **Although Version 3 appropriately introduces cautionary language, using this calculation as a prominent element of the humanitarian-impact analysis risks giving speculative arithmetic greater evidentiary weight than the underlying sources permit. When both the target list figure and the reported accuracy rate are not independently verified, a mathematical extrapolation does not become reliable and this is unacceptable for a scientific journal.

**Required revision: **Further de-emphasise or remove the 3,700-person calculation. The analysis should focus instead on the documented mechanics of high-volume target generation, the limitations of the reported accuracy claim, and the effects of compressed human-review periods, including the reported 20-second review window. These issues can be analysed in relation to IHL precautionary obligations without presenting an unverified estimate as an operational outcome.

**Evidence needed for closure: **Removal or substantial contextualisation of the 3,700-person estimate, together with a revised discussion explaining how high-volume target generation and time-constrained human review may affect the reliability of target verification and the implementation of precautionary measures.

**Major Comment 3: Unresolved Evidentiary Asymmetry**

**Location: **Section 4.4, “Comparative Analysis,” and Figure 3

**Issue: **The comparison between Israeli and Iranian systems is affected by substantial evidentiary asymmetry. Israeli systems, including Lavender and Iron Dome, have comparatively extensive journalistic, OSINT, governmental, and military reporting. By contrast, many claims concerning Iranian swarm coordination and AI-enabled drone systems rely on state announcements and limited publicity available technical evidence. Furthermore, strict state censorship and the long-term blocking of full internet access to the public have also been significant factors contributing to this asymmetry.

**Why this matters: **Applying same capability scale to well-documented operational systems and minimally verified or emerging capabilities may create an unbalanced comparison. Although Section 6.4 acknowledges this limitations, Figure 3 does not visibly communicate the differing levels of evidentiary confidence associated with the individual assessments. Readers may therefore interpret the scores as equally well supported.

**Required revision: **Revise Figure 3 or its accompanying table so that each comparative assessment is paired with an explicit evidentiary-confidence designation derived from the source hierarchy in Section 3.4. These designation should be presented as qualitative source-confidence assessments rather than statistical confidence intervals.

**Evidence needed for closure: **A revised figure, table, or accompanying explanation identifying the evidentiary basis and confidence level for each score, using consistent classifications such as high, moderate, low or emerging evidentiary confidence.

**Major Comment 4: Source-Claim Mismatch in the Analytical Framework**

**Location: **Section 2.1, “Analytical Techniques”

**Issue: **The manuscript attributes a framework consisting of “three primary domains” and “three analytical axes” to Joshi (2018). However, the cited Joshi publication is a brief review of Paul Scharre’s *Army of None*, rather than an original study presenting the analytical framework attributed to it in the manuscript.

**Why this matter: **Attributing core theoretical and analytical categories to a source that does not present them compromises the scholarly foundation and traceability of the study’s methodology.

**Required revision: **Remove the attribution to Joshi for these frameworks. The author should either provide the correct primary source for the stated domains and analytical axes or explicitly identify and comment the framework as the author’s own synthesis.

**Evidence needed for closure: **A correct reference entry and revised text accurately identifying the origin of the analytical framework.

**Major Comment 5 : Mischaracterisation of Article 36 and the Source of Meaningful Human Control**

**Location: **“Key Terminology Definitions,” under “Meaningful Human Control,” and Section 5.3, “Accountability and Legal Responsibility”

**Issue:** The manuscript refers to “ICRC Guidelines NO. 36” as the source of its definition of meaningful human control. Article 36 of Additional Protocol I concerns the legal review of new weapons, means, and methods of warfare. It should not be cited as though it were an ICRC guideline numbered 36 or as the direct source of a specific definition of meaningful human control.

**Why this matters:** Inaccurate identification of a legal instrument in a manuscript centered on IHL compliance undermines the reliability of the legal analysis.

**Required revision: **Correct the definition and sourcing of meaningful human control by citing the relevant source, such as a specific ICRC publication, Article 36 NGO material, or appropriate academic literature. The manuscript must also clearly distinguish the legal obligation to review new weapons under Article 36 of Additional Protocol I from the separate conceptual and policy debate concerning meaningful human control.

**Evidence needed for closure:** Corrected terminology, accurate identification of the relevant legal instrument, and appropriate sourcing for the definition of meaningful human control.

**Major Comment 6 : Bibliographic Inaccuracy and Source Traceability**

**Location: **Section 2.3, “Regional AI Military Development Patterns”; Section 4.2.1 and 4.2.3; and the reference list

**Issue: **The manuscript cites “Rocks W. (2021)” as the author of “Iran’s Bet on Autonomous Weapons.” However, *War on the Rocks* is a publication platform, not an individual author.

**Why this matters:** Beyond formatting, this prevents readers from tracing the source accurately and obscures the authorship and evidentiary credibility of a publication used to support claims concerning Iranian doctrine and technical capabilities. Furthermore, *War on the Rocks* is not a peer-review journal, it is a platform for analysis and debate on strategy, defense, and foreign affairs.

**Required revision:** If you use this citation, identify the actual author of the comment published by *War on the Rocks*, correct the bibliography entry and relevant in-text citations, and verify that the corrected source supports the specific doctrinal and technical claims attributed to it with peer-review journal citations.

**Evidence needed for closure: **A corrected reference entry, corresponding in-text citations, and confirmation that the source supports the claims for which it is cited.

**Minor Comments:**

**Terminological and Typographical Consistency**

 Conduct a complete proofread of AI-related terminology. In several locations, the capital letter “I” in “AI” appears to have been replaced by a lowercase “I” or otherwise rendered inconsistently.

**Figure 1 Clarification**

 Figure 1 is described as a “conceptual diagram constructed by the author.” The manuscript should state explicitly whether the hierarchical command relationships shown in the diagram, including the links between the Supreme National Security Council and the AI-content-generation units, are based on confirmed organisational doctrine, published institutional evidence, or the author’s theoretical modelling.

**Citation and Reference Formatting**

 Several in-text citations appear irregular or incompletely formatted. In addition to correcting the substantive bibliographic problems identified above, the manuscript and reference list should undergo a comprehensive proofread to ensure consistency with the journal’s citation style and to confirm that every in-text citation corresponds accurately to a complete reference-list entry.

**Aspects I Was Unable to Assess**

 I was unable to independently verify classified or otherwise non-public information concerning the internal architectures, training data, operational performance, accuracy rates, and deployment rules of the military systems discussed in the manuscript.

 My assessment is therefore limited to the quality, consistency, and appropriate interpretation of the publicly available evidence cited by the author. I was also unable to determine whether all systems-specific technical claims accurately represent proprietary or classified implementations where no authoritative public documentation was available. I believe that writing a reasonable statement about the certainty of the interpretations made regarding these sources and technical details would be an honest academic contribution.

Is the work clearly and accurately presented and does it cite the current literature?

Partly

If applicable, is the statistical analysis and its interpretation appropriate?

Not applicable

Are all the source data underlying the results available to ensure full reproducibility?

Partly

Is the study design appropriate and is the work technically sound?

Partly

Are the conclusions drawn adequately supported by the results?

Partly

Are sufficient details of methods and analysis provided to allow replication by others?

Partly

Reviewer Expertise:

Military AI applications, autonomous weapon systems, international humanitarian law (IHL), operational targeting architecture, and defense technologies.

I confirm that I have read this submission and believe that I have an appropriate level of expertise to confirm that it is of an acceptable scientific standard, however I have significant reservations, as outlined above.

# Response

**Published:** 2026-09-23

**Authors:** Lamia faris

**Affiliations:** computer science, University of Anbar, Ramadi, Al Anbar Governorate, Iraq

I thank Dr Oktenli for a careful and constructive report. Each major comment is addressed below, with the location of the change in the revised version.

**Major Comment 1 — Conceptual limitations of scoring legal compliance**

 Accepted in full. The 0–100 scoring of legal compliance has been withdrawn. Legal compliance is now reported as a qualitative classification against four requirements — distinction, proportionality, precaution and reviewability — using the categories the reviewer proposes: substantial, partial, minimal and indeterminate. The revised figure presents this as a classification matrix with narrative justification for each cell, and it is retitled "Qualitative assessment of documented compliance mechanisms." A new paragraph beside the classification sets out its inferential limits: the classifications assess whether a system's documented design and employment procedures provide the mechanisms through which human decision-makers can apply the rules; they are not determinations that a system, or any attack, was lawful. The rules bind those who plan and decide upon attacks in the circumstances of each attack and are not properties of an algorithm. "Indeterminate" marks a limit of evidence or applicability, never a finding of non-compliance.

**Major Comment 2 — Extrapolated metrics in the humanitarian-impact analysis**

 Accepted in full. The extrapolation of approximately 3,700 misidentified individuals has been removed from the article. The underlying claim is also explicitly withdrawn: the manuscript now states that an unvalidated error rate whose denominator has never been specified cannot be multiplied by a reported target count to yield a number of misidentified people. The analysis instead addresses what the sources support — the documented mechanics of high-volume target generation, the reported 20-second review window, and the resulting pressure on verification and precaution. A related sentence asserting that civilian casualties demonstrate algorithmic inaccuracy has been replaced: casualties do not by themselves establish algorithmic inaccuracy, and the relationship between the two is not determinable from the available evidence. The 90% accuracy figure is now identified at first mention as an unvalidated operator claim.

**Major Comment 3 — Unresolved evidentiary asymmetry**

 Accepted in full. Every assessment now carries an explicit evidentiary-confidence designation (High, Medium, Low) derived from the source hierarchy, presented as a qualitative source-confidence judgement and not a statistical interval. The former multi-dimensional capability figure has been removed, since it invited exactly the cross-category and national ranking the methods reject. It is replaced by a system-by-indicator matrix in which comparisons are made only within a functional category. Where no Iranian system met the inclusion criteria, the cell records "undetermined," defined as an evidentiary absence rather than a finding of low capability. The manuscript states the interpretive rule directly: an absence of public evidence records the absence of reporting, not the absence of a system.

**Major Comment 4 — Source-claim mismatch in the analytical framework**

 Accepted. The attribution of the "three primary domains" and "three analytical axes" to Joshi (2018) has been removed, and that reference has been deleted from the reference list. The framework is now presented as the author's own synthesis and is distinguished from the frameworks attributed to other scholars.

**Major Comment 5 — Mischaracterisation of Article 36 and the source of meaningful human control**

 Accepted in full. The reference to "ICRC Guidelines NO. 36" was incorrect and has been removed. The definition of meaningful human control is now attributed to Roff and Moyes (2015), written for the NGO Article 36, and is noted as consistent with the ICRC's position on human control in autonomous weapon systems (ICRC, 2021), which has been added to the references. The manuscript now states explicitly that Article 36 of Additional Protocol I imposes an obligation to review new weapons, means and methods of warfare and is not a source of any definition of meaningful human control. The legal discussion separates four questions: whether a weapon has undergone Article 36 review; whether a system's design can support compliance in a given context; whether reported employment procedures appear compatible with distinction, proportionality and precaution; and whether a specific attack was lawful. Reviewability is assessed because it conditions both the review and any later investigation, not as a fourth targeting rule.

**Major Comment 6 — Bibliographic inaccuracy and source traceability**

 Accepted. "Rocks W." was a misreading of the publication platform. The entry is corrected to Lisman EO, "Iran's Bet on Autonomous Weapons," *War on the Rocks*, 30 August 2021, and the in-text citations are corrected accordingly. The entry notes that *War on the Rocks* is a professional analysis platform rather than a peer-reviewed journal, and it is treated at the corresponding level of the source hierarchy. Two further mangled entries were found and corrected in the same audit: a *Military Review* article previously listed as "Gen B, Mcguire RE, Army V" is now McGuire, Slonopas and Olbrych (2025), and a *Harvard International Law Journal* article listed under the journal name is now Anugrah (2024). The primary +972 Magazine / Local Call investigation behind the 37,000-target and 20-second figures has been added, so those claims no longer rest on secondary coverage alone.

**Minor comments**

 AI-related terminology has been proofread throughout and the capitalisation corrected. Figure 1 and the other author-constructed diagrams are identified in their captions as conceptual diagrams drawn by the author and not derived from confirmed organisational doctrine.

# Reviewer report: Reviewer response for version 3

**DOI:** 10.5256/f1000research.202374.r507415

**Published:** 2026-08-08

**Authors:** Drazen Smiljanic

**Affiliations:** 1 Dr.Franjo Tuđan Defense and Security University, Zagreb, Croatia

Comments

 In general, the topic is relevant and suitable for indexing. I haven’t been involved in previous revisions, but it is evident that the third version represents a substantial improvement. It is evident that, during the revision process, the transparency and reproducibility issues have largely been addressed.

 In my opinion, the remaining weakness of the manuscript is primarily the analytical architecture rather than the lack of data (the empirical material is quite extensive). In that regard, the most important revisions concern:

  

- research aim and scope;
- unit of analysis and system taxonomy;
- comparability and interpretation of the scoring framework;
- system-to-national-level inference and evidentiary asymmetry; and
- separation of technical, operational, legal, and humanitarian outcomes.

The strongest opportunity for improvement is to make meaningful human control under conditions of algorithmic scale and compressed decision time, which is the paper's clearer conceptual contribution.

 Points to be addressed (detailed suggestions):

  

- Clarify and sharpen the overall research aim of the manuscript

The aim of the research is stated in the manuscript, but it does not fully correspond to the breadth of the actual analysis. The manuscript describes the aim primarily in terms of AI algorithms in “targeting systems” while the empirical analysis encompasses targeting support, defensive interception, fire-control systems, autonomous platforms & UAVs, swarms, and cyber/information applications. The aim should explicitly state what is being compared, across which dimensions, and for what analytical purpose.

  

- Establish a clear unit of analysis

The manuscript currently appears to move between individual technologies and systems, and Israel and Iran as national cases. I suggest that the author explicitly define the individual AI-enabled military system/application as the primary unit of analysis. It seems more logical that after that, Israel and Iran may constitute the higher-level comparative cases/context within which system-level findings are interpreted.

 In addition, the manuscript should clearly distinguish conclusions derived at the system level from conclusions about broader national approaches to military AI.

  

- Introduce a coherent taxonomy of the systems being compared.

It is because, in practice, Lavender, Besorah, Iron Dome, SMASH, autonomous UAVs, swarm systems, robotic platforms, etc., are functionally heterogeneous. For example, Lavender is essentially an AI-supported target identification/prioritization system. Human oversight concerns the review and authorization of a proposed target. Iron Dome is an automated defensive interception system. Here, speed and automation are intrinsic to the function because an incoming rocket may require a response within seconds. Finally, an autonomous or semi-autonomous UAV/swarm involves yet another set of functions—navigation, coordination, target recognition, potentially engagement, etc.

 In fact, the manuscript already recognizes distinctions between AWS, AI-supported targeting systems, defensive autonomous systems, MHC, and HITL, but these distinctions are not consistently carried through the comparative analysis.

 My suggestion for the author is to consider whether the systems included in the comparison are sufficiently functionally equivalent for the same analytical dimensions and scoring criteria to have comparable meanings across systems. For example, “human oversight” and “operational accuracy” may represent substantially different constructs when applied to an AI-supported target-generation system, an automated defensive interceptor, and an autonomous UAV. On the other hand, if functionally heterogeneous systems are retained, the author should introduce and justify an appropriate functional taxonomy or other classification scheme before undertaking cross-system comparison.

 Such a classification would directly support the clarified research aim. If the study seeks to assess AI-enabled military systems and applications across technical capability, operational employment and effectiveness, human control, and legal compliance, a functional taxonomy will provide the analytical structure necessary to interpret these dimensions in relation to the different purposes and operational contexts of the systems examined. It would also provide a more defensible basis for moving from system-level observations to the higher-level comparison of Israeli and Iranian approaches to military AI.

 This is not to complicate the paper’s update but to ensure that functionally different technologies are not treated as directly equivalent analytical objects.

  

- Clarify the dimensions and indicators of comparison

The manuscript currently uses six dimensions: data integration, algorithmic architecture, human oversight, deployment scale, operational accuracy, and ethical/legal compliance. These, however, do not necessarily represent six indicators of a single underlying construct. Consequently, a clearer hierarchy could organize them into broader dimensions:

 For example, technical capability could be assessed through data integration and algorithmic architecture, while operational employment/effectiveness could encompass deployment scale and operational accuracy. Following the same logic, human control could be assessed through human oversight, and legal compliance through the existing ethical/legal compliance dimension. Such a structure would preserve the existing indicators while clarifying what broader analytical dimensions they are intended to measure.

 In short, I suggest that the author precisely explain what each dimension measures and why it permits meaningful comparison across the included systems.

  

- Strengthen the logic of comparability

This is very important because, even with a common analytical framework, not all systems are directly comparable. For example, “Operational accuracy” means something substantially different for target identification, missile interception, UAV navigation, and cyber operations.

 Similarly, meaningful human control has a different operational significance in a high-speed defensive interception system than in an AI-supported process recommending human targets.

 This is the reason why I suggest the author consider comparison within functional categories first, followed by higher-level comparison of national patterns. Where direct system-to-system comparison is impossible, this should be explicitly acknowledged rather than implied through common scores.

  

- Reconsider aggregation and the apparent precision of the 0–100 scoring framework

Version 3 appropriately clarifies that the scores are analytical assessments rather than statistically validated measurements. However, I am still somewhat concerned that numerical values such as 65, 75, or 85 may suggest a degree of precision that the underlying OSINT and secondary-source evidence cannot necessarily support.

 I therefore suggest that the author explain what analytical information the exact numerical scores provide beyond the Low/Moderate/High categories already defined in the methodology. In particular, the manuscript should clarify how differences between numerical scores within the same category (for example, 75 versus 85) are determined and what substantive meaning should be attributed to such differences. If this level of differentiation cannot be consistently justified, the author may wish to consider whether the ordinal Low/Moderate/High categories alone would provide a more appropriate representation of the evidence.

  

- Clarify how system-level findings support conclusions about national approaches

It is about the clarification that is distinct from the unit-of-analysis issue discussed above. If individual systems are assessed and scored, the manuscript should explain more clearly the analytical step by which findings about these systems are used to characterize broader “Israeli” and “Iranian” approaches to military AI.

 In this respect, the author should clarify the rationale for selecting the systems included in the analysis and the extent to which they can reasonably be considered illustrative of broader national approaches. Otherwise, there is a risk that the resulting characterization of the two national approaches is influenced, at least partly, by the selection of particular systems. The key methodological question is therefore: what justifies moving from findings about the selected systems to conclusions about broader national patterns?

  

- Treat evidentiary asymmetry as an analytical issue, not merely a limitation

The author correctly acknowledges that considerably more detailed evidence is available for Israeli than for Iranian systems and that assessments of Iranian systems should sometimes be interpreted as “emerging indications”. In my view, however, this asymmetry should not only be acknowledged as a limitation - it should also be reflected in the interpretation of the comparative findings.

 In particular, the analysis would benefit from a clearer distinction between documented capability (what can be established from the available sources), assessed capability (what the available evidence suggests about the capabilities of the systems), and evidentiary confidence (the extent to which apparent differences may reflect unequal transparency, source availability, or quality of evidence rather than actual capability differences).

 This is particularly important in the Iranian case. Lower visibility of Iranian capabilities should not automatically translate into an assessment of lower capability. Where the evidence is limited, the manuscript should make clear whether a lower assessment reflects an apparent capability gap or simply greater uncertainty about the capability being assessed..

  

- Maintain a strict distinction between technical accuracy, operational effectiveness, legal compliance, and humanitarian outcomes

Version 3, as I see it, has improved considerably in this respect and currently recognizes multiple interacting technological, operational, command and political explanations for civilian harm. This distinction should be applied consistently throughout the manuscript.

 In real life, civilian casualties cannot by themselves demonstrate algorithmic inaccuracy. Conversely, technically accurate target identification does not establish that an attack was proportionate or otherwise compliant with International Humanitarian Law. Similarly, operational effectiveness does not necessarily imply either legal compliance or favourable humanitarian outcomes. In short, an attack (as a part of the military operation) might kill civilians even though the intended military target was correctly identified. Conversely, civilian casualties do not automatically prove that the AI misidentified the target.

 These concepts should therefore be treated as analytically distinct dimensions rather than implicitly combined within a broader notion of “effectiveness”.

  

- Final considerations

The above comments and suggestions aim at streamlining the flow of logic of the manuscript:

 Research aim -> research questions -> units of analysis -> system taxonomy -> dimensions/indicators -> evidence -> comparison -> national-level findings -> conclusions.

 Currently, most of these elements are present individually, but their relationships are not sufficiently explicit. Therefore, the suggested flow intends to create traceability. A reader (who may be a strategic or defence planner, or someone from industry) can see how the evidence on individual systems leads to broader conclusions about Israeli and Iranian approaches to military AI. That is particularly important if the findings are expected to inform strategic assessment, capability development, defence planning, or policy recommendations.

 Finally, I believe that strengthening this analytical chain would also help the author articulate the scientific contribution (and the “so what?”) of the study more clearly. The main “deliverable” could be the conclusion that Israel emphasizes technologically sophisticated and highly integrated capabilities, while Iran relies more strongly on asymmetric, distributed, and cost-effective solutions. This may be an interesting comparative finding. However, it remains primarily descriptive unless the author explains what can be learned from this comparison beyond the two cases themselves. The manuscript should therefore make more explicit what the Israeli–Iranian comparison contributes to our broader understanding of the development, operational integration, effectiveness, and governance of military AI.

Is the work clearly and accurately presented and does it cite the current literature?

Partly

If applicable, is the statistical analysis and its interpretation appropriate?

Not applicable

Are all the source data underlying the results available to ensure full reproducibility?

Yes

Is the study design appropriate and is the work technically sound?

Partly

Are the conclusions drawn adequately supported by the results?

Partly

Are sufficient details of methods and analysis provided to allow replication by others?

Yes

Reviewer Expertise:

Defence and Security Studies; Military Strategy; Defence Planning and Capability Development; Defence Economics; Military Innovation and Emerging Technologies; Artificial Intelligence and Autonomous Systems in Defence

I confirm that I have read this submission and believe that I have an appropriate level of expertise to confirm that it is of an acceptable scientific standard, however I have significant reservations, as outlined above.

# Response

**Published:** 2026-09-24

**Authors:** Lamia faris

**Affiliations:** computer science, University of Anbar, Ramadi, Al Anbar Governorate, Iraq

I am grateful to Dr Smiljanic for a review that engages with the analytical architecture rather than the surface of the manuscript. His diagnosis — that the weakness lay in the architecture rather than in the evidence — shaped this revision, and his recommended chain (aim → questions → unit of analysis → taxonomy → dimensions → evidence → comparison → national findings → conclusions) is now the organising structure of the article. Each point is addressed below.

**1. Research aim.** Accepted. The aim in §1.1 now states what is compared, across which dimensions, and for what purpose, and says explicitly that the study extends beyond narrowly defined "targeting systems" because the control problem spans all five functions. The stated purpose is no longer to rank two militaries but to isolate the conditions under which meaningful human control is preserved, attenuated or lost.

**2. Unit of analysis.** Accepted in full, in the form suggested. The individual AI-enabled system or application is now the primary unit of analysis, with Israel and Iran as higher-level comparative contexts within which system-level findings are interpreted. A rule of attribution states that every conclusion is marked with the level at which it is warranted, and that no national-level claim is presented as if derived directly from system-level assessment.

**3. Functional taxonomy.** Accepted. §3.4 introduces five functional categories — AI-supported target generation, automated defensive interception, assisted fire control, autonomous platforms and swarms, and cyber and information applications — classified by decision tempo, the position of the human in the decision cycle, and the principal legal test, which is now a column in Table 1. The reviewer's own examples drove this: Lavender's oversight concerns review and authorisation of a proposed target, whereas Iron Dome's automation is intrinsic to a threat measured in seconds. §4.4.2 now carries an explicit interpretive rule that Iron Dome's Low human-oversight rating is not a governance deficiency comparable to Lavender's, since the two are alike in label and opposite in substance.

**4. Dimensions and indicators.** Accepted, and implemented exactly as proposed. The six indicators are now grouped under four higher-order dimensions: technical capability (data integration, algorithmic architecture), operational employment and effectiveness (deployment scale, operational accuracy), human control (human oversight), and legal compliance. A "Final analytical model" paragraph states this once, and the same terminology is used in the methods, tables, figures and conclusions.

**5. Comparability.** Accepted. §3.6 establishes that comparison is made within functional categories first, with national patterns considered only afterwards. Table 6 is read down a column within a category, never across categories, and where direct system-to-system comparison is not possible this is stated rather than implied by a common score.

**6. The 0–100 scoring.** Accepted in full. The numerical scoring has been withdrawn. Capability is reported in ordinal Low / Moderate / High bands with a within-band rubric governing placement, and no composite score is computed. This follows the reviewer's conclusion directly: the distinction between 75 and 85 could not be consistently justified from the underlying evidence, so the ordinal categories alone now represent it. The earlier numerical values are retained in the repository, labelled as superseded analytical inputs.

**7. From system level to national level.** Accepted. §4.5 makes this step explicit and conditional. A pattern is advanced only where it appears across at least two functional categories, is consistent with independently evidenced structural conditions, and is not contradicted by an assessed system. The three resulting propositions are labelled tentative and exploratory, and two statements are marked as *not supported*: relative overall capability, and relative legal compliance. §3.8 gives the system-selection rule and the identified-but-unassessed systems, so the reader can judge how far selection shapes the characterisation. A sensitivity note identifies the FC1 asymmetry as the study's principal open evidentiary question.

**8. Evidentiary asymmetry as an analytical issue.** Accepted, using the reviewer's three-way distinction. The manuscript now separates documented capability, assessed capability, and evidentiary confidence, with a confidence rating attached to every assessment. Where no Iranian system met the inclusion criteria, the result is recorded as *undetermined*, defined as evidentiary absence rather than low capability. The point is stated plainly: a score placing Iran low in FC1 would record the absence of journalism, not the absence of a system.

**9. Technical accuracy, operational effectiveness, legal compliance, humanitarian outcome.** Accepted and applied consistently. These are treated as four distinct constructs throughout. The earlier extrapolation from a reported error rate to a number of misidentified people has been withdrawn, and the manuscript now states that civilian casualties do not by themselves establish algorithmic inaccuracy, nor does correct identification establish proportionality or that feasible precautions were taken.

**10. The analytical chain and the contribution.** Accepted. Figure 1 sets out the chain from aim to conclusions, and §6.5 states three propositions framed to travel beyond the two cases: the volume–time ratio as the binding constraint on meaningful human control, nominal authorisation as its failure mode under high throughput, and the distinction between negative and positive algorithmic authority.

# Reviewer report: Reviewer response for version 3

**DOI:** 10.5256/f1000research.202374.r494413

**Published:** 2026-07-03

**Authors:** Ananya Pandey

**Affiliations:** 1 Christ University, Bangalore, Karnataka, India

The manuscript addresses a highly relevant and contemporary issue concerning the integration of artificial intelligence into military operations and its implications for international law, humanitarian protection, and global governance.

 The discussion demonstrates a strong understanding of the strategic, technological, and regulatory dimensions of AI-enabled warfare and presents a thoughtful comparative perspective on different approaches to military AI deployment. The writing is generally clear, well organized, and analytically engaging.

 One of the manuscript’s main strengths is its effort to balance considerations of operational effectiveness with ethical and humanitarian concerns. The comparative analysis provides useful insights into how varying levels of technological sophistication and resource availability shape military AI strategies and governance challenges.

 However, several areas could be strengthened. Some claims would benefit from more explicit evidentiary support and clearer distinction between documented findings, analytical interpretations, and normative concerns. The manuscript should also engage more deeply with relevant international legal principles and existing debates on meaningful human control, accountability, transparency, and civilian protection. Greater attention to methodological limitations, particularly regarding conflict-zone data and publicly available sources, would improve analytical rigor. In addition, the policy discussion could be enhanced by outlining more specific and actionable governance mechanisms.

Is the work clearly and accurately presented and does it cite the current literature?

Partly

If applicable, is the statistical analysis and its interpretation appropriate?

Partly

Are all the source data underlying the results available to ensure full reproducibility?

Partly

Is the study design appropriate and is the work technically sound?

Yes

Are the conclusions drawn adequately supported by the results?

Partly

Are sufficient details of methods and analysis provided to allow replication by others?

Yes

Reviewer Expertise:

Human Rights, IPRs, Climate Change, AI, Ethics

I confirm that I have read this submission and believe that I have an appropriate level of expertise to confirm that it is of an acceptable scientific standard, however I have significant reservations, as outlined above.

# Response

**Published:** 2026-10-09

**Authors:** Lamia faris

**Affiliations:** computer science, University of Anbar, Ramadi, Al Anbar Governorate, Iraq

I thank Dr Pandey for a careful reading and for the generous assessment of the manuscript's scope and organisation. Her four concerns — evidentiary support, engagement with the legal literature, methodological limitations, and the specificity of the governance proposals — each prompted substantive revision. They are addressed below.

**1. Evidentiary support, and the distinction between documented findings, analytical interpretations, and normative concerns**

 Accepted in full, and this is now a structural feature of the article rather than a matter of wording.

 Three levels are kept apart throughout. *Documented capability* is what can be established at the upper tiers of the source hierarchy; *assessed capability* is what the available evidence supports once lower-tier material is admitted; and an *evidentiary-confidence rating* (High, Medium, Low) is attached to every individual assessment. Where no system met the inclusion criteria, the result is recorded as **undetermined**, defined explicitly as an evidentiary absence rather than a finding of low capability.

 Several specific claims were withdrawn or qualified as a result. The numerical scoring of capability has been replaced by ordinal bands, so no figure now implies a precision the sources cannot carry. Descriptions of neural networks, deep-learning pipelines, convolutional architectures, training data and fuzzy-logic engines have been deleted or qualified wherever no source documents them, and the manuscript now defines artificial intelligence, algorithmic automation and autonomy separately so that automated functions are not described as AI without evidence. Reported performance figures carry their status at first mention: the Lavender accuracy figure is identified as an unvalidated operator claim whose denominator has never been specified, and the Iron Dome interception rate as an official claim contested in independent analysis.

 Normative claims are likewise separated from findings. The governance proposals in §6.3 are presented as recommendations derived from identified failure modes, and their feasibility ratings are explicitly identified as the author's assessments rather than empirical results.

**2. Engagement with international legal principles and the debates on meaningful human control, accountability, transparency and civilian protection**

 Accepted. The legal discussion has been both deepened and more tightly bounded.

 On *meaningful human control*, the definition is now attributed to Roff and Moyes (2015), written for the NGO Article 36, and noted as consistent with the ICRC's position on autonomous weapon systems (ICRC, 2021). An earlier version wrongly cited "ICRC Guidelines No. 36," conflating the concept with Article 36 of Additional Protocol I; the manuscript now states that Article 36 imposes an obligation to review new weapons, means and methods of warfare and is not a source of any definition of meaningful human control. The wider debate is engaged through Santoni de Sio and van den Hoven (2018), Amoroso and Tamburrini (2020), Ekelhof (2019), Roff and Moyes (2015), Sparrow (2007), Asaro (2012), Schmitt and Thurnher (2013) and Human Rights Watch (2012), together with the CCW Group of Governmental Experts' Guiding Principles (2019) and UN General Assembly Resolution 78/241 (2023).

 On *accountability*, the treatment of individual criminal responsibility now cites the relevant provisions of the Rome Statute (1998) — Articles 25 and 30 on individual responsibility and the mental element, and Article 28 on superior responsibility — and states plainly that whether a programmer's design choices could satisfy those standards is unsettled and untested, and that the opacity or autonomy of a system does not by itself establish anyone's criminal responsibility. The distinction between state responsibility and individual criminal responsibility is set out, and it is the reason the governance proposals emphasise record preservation and reviewability rather than individual attribution.

 On *transparency*, the manuscript follows Holland Michel (2020) in distinguishing predictability from understandability, and proposes **reviewability** — the capacity to reconstruct after the fact why a particular engagement occurred — as the property that a legal review or investigation actually requires.

 On *civilian protection*, the four requirements of distinction, proportionality, precaution and reviewability now structure the legal assessment, with the precautionary obligation under AP I Article 57 stated in full, including the duty to give effective advance warning.

**3. Methodological limitations, particularly conflict-zone data and publicly available sources**

 Accepted. Limitations are now treated as an analytical condition shaping interpretation, not as a disclaimer at the end.

 The evidence rests on a five-level source hierarchy running from technical documentation and official acknowledgement, through attributable investigative reporting and institutional OSINT analysis, to manufacturer and operator claims; where sources conflict, the lower level is used unless there is specific evidence for the higher. The asymmetry between the two cases is explicit: far more is publicly documented about Israeli systems, and the manuscript states that a low score for Iran in target generation would record the absence of journalism rather than the absence of a system. The absence of Persian-language source access is disclosed. Because all coding was performed by one researcher, the manuscript now states that the rubric structures and documents these judgements but does not eliminate their subjectivity, and a sensitivity note identifies which finding is most vulnerable to revision if new evidence emerges.

 The full coding — per-indicator assessments, confidence ratings, the band rubric and the legal classifications — is deposited in the public repository so that the judgements can be audited independently.

**4. More specific and actionable governance mechanisms**

 Accepted, and this is where the policy discussion changed most. Seventeen mechanisms are proposed, each specifying the actor responsible, the instrument through which it would be implemented, and the indicator by which compliance could be verified. They are grouped by the point at which control can be exercised: system design and procurement (A1–A5), operational employment (B1–B4), legal review and accountability (C1–C4), and international coordination (D1–D4).

 Following this review, each mechanism is now linked explicitly to the failure mode it addresses. Volume–time saturation is met by throughput ceilings and minimum deliberation time, organizational separation of nomination from strike authorization, and automatic escalation when throughput exceeds review capacity. Loss of intervention capacity at commitment is met by abort or recall capability and encoded engagement limits. The inability to reconstruct an individual engagement is met by a reviewability specification, recording of collateral damage estimation inputs, and preservation of targeting logs and review timestamps. Drift between the system reviewed and the system deployed is met by extending the Article 36 review to employment tempo and throughput and by re-reviewing the model update or retraining.

**On the "Partly" ratings**

 I hope the revisions speak to each. Presentation and literature: the legal and human-control literature is now engaged directly, and the reference list has been audited in both directions, with the primary investigative source for the central empirical figures added. Statistical analysis: no statistical inference is now claimed anywhere; assessments are ordinal and qualitative, with confidence attached. Reproducibility: the full coding is deposited, and the codebook is published with it. Conclusions supported: claims are now tied to the level at which they are warranted, with national-level statements labelled tentative propositions from a non-representative sample, and two statements—relative overall capability and relative legal compliance—marked explicitly as not supported.

# Reviewer report: Reviewer response for version 2

**DOI:** 10.5256/f1000research.197018.r486079

**Published:** 2026-05-22

**Authors:** Adebayo Yusuf Balogun

**Affiliations:** 1 University of Tampa, Tampa, FL, USA

This article examines the growing use of artificial intelligence in military targeting systems within the Iranian-Israeli confrontation, with particular attention to technical architectures, operational deployment, legal thresholds, and the limits of human oversight. The topic is highly relevant and timely, especially given increasing global concern regarding autonomous and AI-assisted warfare. The manuscript attempts to bridge technical analysis, international humanitarian law, and regional security studies through a comparative framework.

 The article’s main strengths include its interdisciplinary scope, clearly stated research questions, structured organization, and engagement with recent discussions on AI-enabled warfare. The comparative approach between Israeli and Iranian systems is particularly valuable and contributes to ongoing debates regarding military AI governance and meaningful human control.

 At the same time, several methodological and evidentiary concerns should be addressed before the article can be considered fully scientifically sound.

 1. Is the work clearly and accurately presented and does it cite the current literature?

**Answer: Partly**

 The manuscript is generally well written and incorporates recent literature relevant to AI military applications, autonomous systems, and international humanitarian law. The structure is logical, and the discussion is accessible to both technical and policy-oriented readers.

 However, several claims are presented too definitively despite relying on investigative journalism, anonymous sources, OSINT material, or unverified operational reporting. In some sections, distinctions between confirmed evidence, allegations, analytical interpretation, and speculation are insufficiently clear. This is especially important when discussing operational accuracy, casualty attribution, and specific targeting practices.

 Recommendations

- Clearly differentiate verified information from reported or alleged claims.
- Use more cautious language when discussing contested operational details.
- Separate peer-reviewed academic evidence from media investigations and commentary.
- Strengthen engagement with scholarly literature on AI accountability, explainability, and military human-machine interaction.

2. Is the study design appropriate and is the work technically sound?

**Answer: Partly**

 The comparative case-study design is appropriate for the subject matter, and the six-dimensional analytical framework provides a useful structure for evaluation. The paper successfully integrates technical, operational, ethical, and legal dimensions into a single comparative analysis.

 Nevertheless, the technical scoring framework requires greater transparency. Numerical capability scores create an appearance of precision, but the criteria used to assign these scores are not sufficiently explained. Some evaluations appear subjective and difficult to independently verify.

 Must Be Addressed

- Explain in detail how the scoring process was conducted.
- Clarify whether scoring was performed by a single evaluator or multiple reviewers.
- Describe how uncertainty and conflicting evidence were handled during scoring.
- Include explicit justification for each major comparative score.

Additional Suggestions

- Consider reducing reliance on numerical scoring where objective measurement is not possible.
- Acknowledge the limitations of assessing classified or partially documented military systems.

3. Are sufficient details of methods and analysis provided to allow replication by others?

**Answer: Partly**

 The methodology section includes useful information regarding source selection, inclusion and exclusion criteria, and analytical dimensions. The discussion of source verification procedures is also valuable.

 However, the article does not yet provide enough methodological detail to enable full replication of the comparative analysis. The scoring framework, coding procedures, and weighting methodology remain insufficiently documented.

 Must Be Addressed

- Provide a methodological appendix or supplementary table showing:
  - Evidence sources used for each system
  - Score justification for each analytical dimension
  - Reliability assessment of each source
- Clarify whether any coding framework or standardized rubric was used.
- Explain how contradictory sources were reconciled in practice.

4. If applicable, is the statistical analysis and its interpretation appropriate?

**Answer: Not applicable**

 The study is primarily qualitative and comparative rather than statistical. Numerical values are descriptive and illustrative rather than inferential. No major concerns arise in this area.

 However, the manuscript should explicitly state that the numerical comparisons are analytical assessments rather than statistically validated measurements.

 5. Are all the source data underlying the results available to ensure full reproducibility?

**Answer: Partly**

 The inclusion of supplementary datasets and evaluation materials is a positive feature and improves transparency. The effort to provide public access to source documentation is commendable.

 Nonetheless, complete reproducibility remains difficult because many underlying claims rely on non-public, classified, or unverifiable sources, including investigative reports, military statements, and OSINT material.

 Recommendations

- Clearly identify which findings are based on independently verifiable evidence and which rely on contested reporting.
- Include a source reliability classification system.
- Clarify the limits of reproducibility given the nature of military and intelligence-related research.

6. Are the conclusions drawn adequately supported by the results?

**Answer: Partly**

 The conclusions are generally consistent with the broader analysis and appropriately emphasize concerns regarding accountability, meaningful human control, and legal governance of AI-enabled military systems.

 However, some conclusions concerning humanitarian outcomes and operational causation go beyond what the available evidence can conclusively establish. In particular, stronger caution is needed when attributing civilian casualties directly to AI systems rather than to broader operational and command decisions.

 Must Be Addressed

- Moderate causal claims regarding civilian harm and AI targeting systems.
- Distinguish clearly between technological capability, operational use, and political decision-making.
- Reframe some conclusions as informed concerns or analytical interpretations rather than established facts.

Overall Recommendation

 The article addresses an important and emerging area of research and has the potential to make a meaningful contribution to discussions on military AI, international humanitarian law, and algorithmic warfare. The interdisciplinary framing and comparative perspective are notable strengths.

 Before publication, however, the manuscript requires revisions aimed at improving methodological transparency, evidentiary precision, and analytical balance.

 Key Revisions Required for Scientific Soundness

1. Clarify the evidentiary basis for all major operational and technical claims.
2. Provide transparent and reproducible justification for the scoring framework.
3. Distinguish clearly between verified evidence, allegations, and interpretation.
4. Moderate unsupported causal conclusions regarding humanitarian impacts.
5. Expand methodological documentation to improve replicability.

With these revisions, the manuscript would provide a stronger and more credible contribution to scholarship on AI-enabled warfare and international security governance.

Is the work clearly and accurately presented and does it cite the current literature?

Partly

If applicable, is the statistical analysis and its interpretation appropriate?

Not applicable

Are all the source data underlying the results available to ensure full reproducibility?

Partly

Is the study design appropriate and is the work technically sound?

Partly

Are the conclusions drawn adequately supported by the results?

Partly

Are sufficient details of methods and analysis provided to allow replication by others?

Partly

Reviewer Expertise:

Cybersecurity, Cybersecurity Governance, Risk Management, Artificial Intelligence Security, Cloud Security, Data Privacy, Threat Intelligence, Digital Governance, Compliance and Regulatory Security, Vulnerability Management

I confirm that I have read this submission and believe that I have an appropriate level of expertise to confirm that it is of an acceptable scientific standard, however I have significant reservations, as outlined above.

# Response

**Published:** 2026-05-23

**Authors:** Lamia faris

**Affiliations:** computer science, University of Anbar, Ramadi, Al Anbar Governorate, Iraq

Dear Reviewer,

 Thank you for your thorough, constructive, and highly detailed evaluation of our manuscript. Your comments have been instrumental in strengthening the methodological transparency, evidentiary precision, and analytical balance of the paper. We are grateful for both the recognition of the manuscript’s interdisciplinary scope and comparative value, and for the specific guidance on areas requiring improvement.

 Below, we provide a detailed point-by-point response to each of your comments, organized according to the six evaluation questions in your report. For each concern, we indicate the specific revisions made. Reviewer comments are shown in ***bold italics***, and author responses follow. References to manuscript sections correspond to the revised version.

 1. Clarity of Presentation and Current Literature

 1.1 Differentiating verified from alleged claims

**Reviewer: *"Several claims are presented too definitively despite relying on investigative journalism, anonymous sources, OSINT material, or unverified operational reporting. Distinctions between confirmed evidence, allegations, analytical interpretation, and speculation are insufficiently clear."***

**Response: **We fully agree. The following revisions have been made:

**Hedging language: **A comprehensive language audit has been performed throughout the manuscript. Claims based on investigative journalism or anonymous sources now use cautious language such as “according to investigative reporting,” “according to reports,” “basing their claim on anonymous Israeli intelligence officers,” and “according to humanitarian affairs reports and local health authorities.” These phrases consistently signal the provenance and reliability level of each claim.

**Source reliability classification: **A formal four-tier source reliability classification system (T1–T4) has been developed and applied throughout the manuscript. Section 3.4 now explicitly states that this hierarchy serves as a source reliability classification, with claims supported by T1/T2 sources presented with greater certainty and those based on T3/T4 sources accompanied by hedging language. The full classification system is documented in the new Supplementary Scoring Justification Table (Sheet 2: Source Reliability), deposited on Zenodo (DOI: 10.5281/zenodo.17385265).

**Performance metrics: **The Lavender system’s 90% accuracy claim is now explicitly attributed to “Unit 8200 internal validation studies” (self-reported military figures), and the text states that “independent validation of the system’s performance remains unavailable due to operational secrecy.” The 3,700 misidentification figure is explicitly flagged as “a hypothetical misidentification figure—an extrapolation that must be treated with caution given that the underlying accuracy claim itself remains unverified through independent assessment.”

 1.2 Separating peer-reviewed from media evidence

**Reviewer: *"Separate peer-reviewed academic evidence from media investigations and commentary."***

**Response: **The expanded methodology (Section 3.2) now explicitly categorizes sources into five types: academic databases (Web of Science, Scopus, JSTOR, Google Scholar), OSINT platforms, government/military documents, investigative journalism outlets, and legal/humanitarian sources. The source priority hierarchy in Section 3.4 ranks these from (a) independently confirmed technical documentation to (e) anonymous source reporting. In the Supplementary Scoring Justification Table, every score is accompanied by the specific sources used and their tier classification, enabling readers to assess the evidentiary weight of each finding.

 1.3 Scholarly engagement

**Reviewer: *"Strengthen engagement with scholarly literature on AI accountability, explainability, and military human-machine interaction."***

**Response: **The revised manuscript engages more deeply with scholarly literature in this area, including expanded discussion of Scharre (2018) on human control spectrums and the human-in-the-loop/out-of-the-loop taxonomy, Roff and Moyes (2015) on meaningful human control aligned with ICRC and Article 36 frameworks, Ekelhof (2019) on the relationship between AI-speed decision-making and responsibility, Sharkey (2012) on the responsibility gap in lethal autonomous weapons, and Mauri (2022) on autonomous weapons under international humanitarian law. A new Key Terminology Definitions section aligns core concepts with established scholarly definitions, including MHC consistent with ICRC Guidelines No. 36.

 2. Study Design and Technical Soundness

 2.1 Scoring framework transparency (Must Be Addressed)

**Reviewer: *"Explain in detail how the scoring process was conducted. Clarify whether scoring was performed by a single evaluator or multiple reviewers. Describe how uncertainty and conflicting evidence were handled during scoring. Include explicit justification for each major comparative score."***

**Response: **This was among the most critical observations, and we have addressed each point comprehensively:

**Scoring criteria: **A new Table A (“Scoring criteria for six-dimension framework”) has been added to Section 3.3, providing explicit definitions for Low (0–30), Moderate (31–60), and High (61–100) capability levels across all six dimensions. Each cell contains specific criteria that must be met for a given score range.

**Single evaluator: **The manuscript now explicitly acknowledges that all evaluations were performed by a single researcher, which constitutes a methodological limitation. The text states: “In the absence of multiple independent raters, subjective bias in scoring cannot be fully excluded. To mitigate this, the author applied the standardized rubric in Table A consistently across all systems and relied on the source priority hierarchy in Section 3.4 to ensure evidence-based scoring.”

**Uncertainty and conflict handling: **Section 3.4 (Priority Level) describes the five-level hierarchical reconciliation process used when sources conflicted, ranging from (a) independently confirmed technical documentation to (e) anonymous source reporting. Conflict notation procedures require that serious disagreements are stated explicitly. A minimum estimation rule applies when quantitative estimates are contradictory.

**Per-score justification: **A comprehensive Supplementary Scoring Justification Table has been deposited on Zenodo (DOI: 10.5281/zenodo.17385265, Version 2.0). This workbook contains 48 individual score justifications (8 systems × 6 dimensions), each specifying: the score assigned, capability level, primary sources used, source tier(s), written justification, and a confidence flag (High/Medium/Low). This directly provides the “explicit justification for each major comparative score” the reviewer requested.

 2.2 Numerical scoring limitations (Additional Suggestion)

**Reviewer: *"Consider reducing reliance on numerical scoring where objective measurement is not possible. Acknowledge the limitations of assessing classified or partially documented military systems."***

**Response: **We have addressed both points:

**Analytical disclaimer: **Section 3.3 now includes an explicit statement: “These scores represent qualitative analytical assessments by the author based on the documented evidence hierarchy, rather than statistically validated measurements. The scoring framework is intended to facilitate structured comparison and visual representation of relative capabilities, not to convey measurement precision.” This language is also reflected in figure captions.

**Classified systems: **A new paragraph at the end of Section 4.3 acknowledges: “Military AI systems are inherently classified, and publicly available information represents only a fraction of actual technical capabilities and operational practices. The assessments presented in this study are necessarily constrained by what has been disclosed, leaked, or independently documented.” A new dedicated Limitations subsection (Section 6.4) further elaborates on data access, methodological constraints, evidence asymmetry, and scope limitations.

 3. Methodological Replication

 3.1 Methodological appendix (Must Be Addressed)

**Reviewer: *"Provide a methodological appendix or supplementary table showing: evidence sources used for each system, score justification for each analytical dimension, reliability assessment of each source."***

**Response: **This has been fully addressed through the new Supplementary Scoring Justification Table deposited on Zenodo (DOI: 10.5281/zenodo.17385265, Version 2.0). The workbook contains five sheets: (1) Scoring Justification — 48 rows with system, dimension, score, sources, source tiers, written justification, and confidence flag for every assessment; (2) Source Reliability Classification — the T1–T4 tiering system with examples and required hedging language; (3) Scoring Rubric — full Low/Moderate/High definitions for all six dimensions; (4) IHL Compliance Assessment — five IHL principles scored for both Israeli and Iranian systems with article references and justifications; (5) Metadata — DOI, license, versioning, and analytical disclaimer. This directly provides all three elements the reviewer requested: evidence sources per system, score justification per dimension, and reliability assessment per source.

 3.2 Coding framework and contradictory sources

**Reviewer: *"Clarify whether any coding framework or standardized rubric was used. Explain how contradictory sources were reconciled in practice."***

**Response: **Table A in the manuscript now serves as the standardized rubric, defining explicit criteria for Low (0–30), Moderate (31–60), and High (61–100) across each dimension. Section 3.4 describes the hierarchical reconciliation process applied when sources conflicted, including the five-level priority hierarchy, conflict notation procedures requiring explicit statement of disagreements, and the minimum estimation rule for contradictory quantitative data. New subsections 3.2.2 and 3.2.3 specify four inclusion criteria (technical detail, evidentiary support, source credibility, cross-verification) and four exclusion criteria (unverified information, speculative capabilities, non-attributable sources, internal contradictions).

 4. Statistical Analysis

**Reviewer: *"The manuscript should explicitly state that the numerical comparisons are analytical assessments rather than statistically validated measurements."***

**Response: **Agreed and implemented. Section 3.3 now contains an explicit disclaimer stating that scores represent qualitative analytical assessments, not statistical measurements. Figure captions have been updated to state the analytical basis of the visualizations. For example, Figure 3 states: “Source: Author’s comparative analysis based on documented system specifications”; Figure 4 states: “Source: Author’s compliance analysis based on IHL framework evaluation.” The Supplementary Scoring Justification Table metadata sheet reiterates this disclaimer.

 5. Source Data and Reproducibility

 5.1 Verifiable vs. contested evidence

**Reviewer: *"Clearly identify which findings are based on independently verifiable evidence and which rely on contested reporting. Include a source reliability classification system."***

**Response: **The five-level priority hierarchy in Section 3.4 now explicitly serves as a source reliability classification system, and the text states that “claims supported by Level (a) or (b) sources are presented with greater certainty, while those based primarily on Level (d) or (e) sources are accompanied by appropriate hedging language.” The Supplementary Scoring Justification Table assigns source tier(s) and a confidence flag (High/Medium/Low) to every individual score, enabling readers to assess precisely which findings rest on independently verifiable evidence (T1) and which rely on contested or single-source reporting (T3–T4). Of the 48 scores in the table, 6 are rated High confidence, 22 Medium, and 20 Low, reflecting the inherent evidential constraints of military AI research.

 5.2 Limits of reproducibility

**Reviewer: *"Clarify the limits of reproducibility given the nature of military and intelligence-related research."***

**Response: **Section 3.2 explicitly discusses this: “Information on conflict areas is both extremely biased and imprecise due to restricted access to the field, the prevalence of propaganda, and the absence of comprehensive ground-truth verification.” The new dedicated Limitations subsection (Section 6.4) further addresses this with specific discussion of: data access and verification limitations (classified systems, propaganda, no ground-truth); methodological constraints (secondary data only, single evaluator, no primary interviews or technical access); evidence asymmetry between Israeli and Iranian systems; and scope limitations (two states, one region, rapidly evolving technology). The data availability section acknowledges this is a secondary data analysis with no primary data collection.

 6. Conclusions and Causal Claims

 6.1 Moderating causal claims (Must Be Addressed)

**Reviewer: *"Moderate causal claims regarding civilian harm and AI targeting systems. Distinguish clearly between technological capability, operational use, and political decision-making."***

**Response: **This was a critical observation that we have addressed thoroughly:

**Multiple hypotheses: **The humanitarian impact section (Section 6.2) has been revised to present the disconnect between claimed precision and observed outcomes as multiple intersecting factors rather than a single causal claim. The revised text states: “These include potential limitations in AI targeting accuracy, inadequate human oversight mechanisms, broader operational and command decisions that may prioritize military objectives over civilian protection, and political decision-making processes that determine rules of engagement.” This explicitly distinguishes technological capability from operational use from political decision-making, as the reviewer requested.

**Attribution caveat: **The revised text includes an explicit methodological caveat: “It is important to note that civilian casualties in armed conflict result from complex chains of technological, operational, and political decisions, and attributing specific outcomes solely to AI system performance would oversimplify this relationship.”

 6.2 Reframing as analytical interpretations (Must Be Addressed)

**Reviewer: *"Reframe some conclusions as informed concerns or analytical interpretations rather than established facts."***

**Response: **The conclusions section (Section 7) has been revised to use explicitly interpretive framing. The paragraph discussing Gaza operations now reads: “These reports, while subject to the verification limitations inherent in active conflict zones, suggest a concerning pattern that warrants further independent investigation.” It concludes: “These observations should be understood as informed analytical concerns based on available evidence rather than definitive causal conclusions.” The phrase “apparent tension between stated precision objectives and documented civilian harm—whether attributable to technological limitations, operational tempo, command decisions, or their interaction” explicitly presents the finding as an analytical concern with multiple possible explanations.

 Additional: Dedicated Limitations Subsection

**Reviewer: *"A dedicated limitations subsection would improve the manuscript." (Implied across multiple evaluation questions)***

**Response: **A new Section 6.4 (“Study Limitations”) has been added before the Conclusions, consolidating all limitations into a single dedicated subsection. It clearly distinguishes four categories: (1) Data access and verification limitations (classified systems, propaganda, no ground-truth verification, source reliability constraints); (2) Methodological constraints (secondary data only, single evaluator, no inter-rater reliability, no primary interviews or technical access); (3) Asymmetry in evidence (Israeli systems more documented than Iranian, reflecting transparency differences not analytical effort, Iranian assessments framed as “emerging indications”); (4) Scope limitations (two states, one region, rapidly evolving technology). This consolidation addresses concerns raised in evaluation questions 1, 2, 3, 5, and 6.

 Additional: Supplementary Data Update

**Response: **The Zenodo repository (DOI: 10.5281/zenodo.17385265) has been updated to Version 2.0 with the addition of the Supplementary Scoring Justification Table. This Excel workbook contains five sheets: (1) Scoring Justification with 48 per-system, per-dimension entries including sources, tiers, justifications, and confidence flags; (2) Source Reliability Classification (T1–T4); (3) Full Scoring Rubric; (4) IHL Compliance Assessment; (5) Metadata with analytical disclaimer. The Data Availability section in the manuscript now explicitly maps which figures and tables are derived from this dataset. All supplementary data remain released under CC-BY 4.0.

 Summary of Revisions

*The following table summarizes how each “Must Be Addressed” item has been resolved:*

**1. Evidentiary basis for claims **→ Language audit + T1–T4 source classification + hedging language throughout (Sections 3.2, 3.4, throughout)

**2. Scoring framework transparency **→ Table A + single evaluator acknowledgment + analytical disclaimer + Supplementary Table on Zenodo (Section 3.3, Zenodo)

**3. Verified vs. alleged evidence **→ Five-level priority hierarchy as reliability classification + per-score confidence flags (Section 3.4, Supplementary Table)

**4. Causal conclusions moderated **→ Multiple hypotheses framing + tech/operational/political distinction + “informed analytical concerns” language (Sections 6.2, 7)

**5. Methodological replicability **→ Inclusion/exclusion criteria + scoring rubric + supplementary justification table + dedicated Limitations subsection (Sections 3.2.2, 3.2.3, 3.3, 6.4, Zenodo)

 We sincerely thank the reviewer for the exceptionally detailed and constructive evaluation. The feedback has materially improved the methodological transparency, evidentiary precision, and analytical balance of the manuscript. We believe the revisions fully address all “Must Be Addressed” points and the majority of additional suggestions, and we welcome any further feedback.

 Respectfully,

**Lamia Faris**

 College of Dentistry, Basic Science

 University of Anbar, Iraq

 den.lamia.faris@uoanbar.edu.iq

# Reviewer report: Reviewer response for version 1

**DOI:** 10.5256/f1000research.187170.r436966

**Published:** 2025-12-26

**Authors:** Elshan Hashimov

**Affiliations:** 1 National Defense University, Baku, Azerbaijan

**1. Summary of the article**

 The article analyzes the use of artificial intelligence in military targeting systems in the Iranian - Israeli confrontation. It focuses on technical architecture, operational practices, and compliance with international humanitarian law (IHL). The author examines Israeli systems such as Lavender, Besorah, SMASH fire control systems, Iron Dome, and specialized robotic platforms (IRIS and Goshawk) and compares them with Iranian developments in autonomous drones, swarm systems, AI enhanced missiles (Mobin with DSMAC guidance), and AI supported cyber and information operations.

 Methodologically, the article uses secondary data from investigative journalism, OSINT, military documents, and academic literature, evaluated using a six-dimension analytical framework: data integration, algorithmic architecture, human oversight, deployment scale, operational accuracy, and ethical or legal compliance.

 The results emphasize higher technological sophistication, data fusion capacity, and integrated architectures on the Israeli side, and a more asymmetric, cost effective, and proxy-oriented approach on the Iranian side. The author then assesses these systems against IHL principles (distinction, proportionality, precaution) and discusses issues of meaningful human control, accountability, and strategic stability, arguing that current uses of AI in targeting create serious risks for civilians and increase escalation pressures, and that robust international governance is urgently required.

**2. Major comments (issues that should be addressed)**

**2.1 Study design and methodological transparency**

 The overall mixed methods comparative design is reasonable for a topic where primary empirical access is highly constrained. However, to be technically sound and replicable the methodology needs to be specified more rigorously.

1. **Source selection and search strategy**

The paper lists broad categories of sources (journal articles, authority documents, OSINT, investigative journalism) and general quality criteria, but does not give a clear search strategy, time window, or inclusion and exclusion criteria.

 It would substantially improve reproducibility if the author specified:

-
  -
    - Databases and platforms searched (for example Web of Science, Scopus, specific OSINT platforms, named media outlets).
    - Time frame for documents.
    - Language limitations, if any.
    - Explicit inclusion and exclusion criteria (for example, only sources with independently verifiable evidence, only technical descriptions above a certain level of detail).
    - Whether any formal protocol (systematic review style) was followed.

These clarifications are essential for other researchers to reproduce or update the analysis.

1. **Operationalization of the six-dimension framework**

The six analytical dimensions are conceptually clear, but it is not explained how systems were scored or compared on each dimension.

 Tables and figures (for example the comparative technology capability charts and matrices) appear to present qualitative judgements, but the underlying coding scheme, rating scales, and decision rules are not described.

 Please add:

1.
  -
    - Definitions for what constitutes low, medium, or high capability on each dimension.
    - How conflicting sources were reconciled when assigning a rating.
    - Whether any inter rater checking or sensitivity analysis was performed (even informally).
2. **Use of quantitative performance numbers**

The article cites specific performance metrics such as a reported 90 percent accuracy for Lavender and 5 - 10 percent error rates for AI targeting in general.

 These numbers are crucial for later humanitarian impact estimates (for example the extrapolation from 37,000 targets to approximately 3,700 potential misidentifications).

 The article should:

- Clearly reference each numeric claim to its original source.
- Explain whether these are self-reported military validation figures, independent estimates, or journalistic reconstructions.
- Distinguish clearly between documented data and author extrapolations, and, where extrapolations are made, state assumptions and uncertainties explicitly.

At present, the conclusions about error rates and humanitarian impact are plausible but methodologically under specified.

**2.2 Scope of claims versus quality of evidence**

 The paper sometimes moves from descriptive or qualitative evidence to strong normative or causal claims (for example that certain systems are a primary cause of unprecedented civilian casualties) without always marking the inferential step.

 The discussion of Gaza casualty figures and allegedly systematic practices such as striking targets at home at night is heavily based on investigative journalism and anonymous sources.

 This material is important and topical, but to keep the article scientifically robust:

-
  - Clearly separate what is documented and cross checked from what remains contested or partially corroborated.
  - Use cautious language (for example "reported practices" or "alleged protocols") where the evidence base is primarily journalistic or based on anonymous testimonies.
  - Make more explicit the uncertainty surrounding any causal linkage between specific AI systems and observed casualty patterns.

The conclusions should reflect this graded evidential status more consistently.

**2.3 Balance between Israeli and Iranian empirical depth**

 The empirical material on Israeli systems (Lavender, Besorah, Iron Dome, SMASH, IRIS, Goshawk) is relatively detailed at both technical and operational levels.

 By contrast, the treatment of Iranian systems, especially drone swarms, ground robots, and cyber operations, relies more on high level descriptions and generalized assessments of "increasing sophistication" without comparable technical granularity.

 To strengthen the comparative purpose of the article, I suggest:

- Either enriching the Iranian side with more technical and operational detail (where available), or
- Making explicit that the comparison is asymmetrical because of differences in transparency and data availability and framing some Iranian sections more tentatively as "emerging indications" rather than symmetric case studies.

**2.4 Ethical and legal analysis structure**

 The ethical and legal sections are a strong element of the paper, but they would benefit from clearer structuring and closer linkage to the earlier technical analysis.

 The discussion of distinction, proportionality, and precaution is well aligned with IHL, but sometimes read as a general critique of AI in warfare rather than a structured evaluation of the systems previously described.

 I recommend:

-
  - For each main system group (for example Israeli targeting systems, Israeli defensive systems, Iranian drones, Iranian cyber operations) briefly stating how they fare with respect to each principle, using the six-dimension framework.
  - More explicitly relating the proposed international regulatory measures in Table 7 to specific technical or organizational deficiencies identified earlier.

This would make the paper more analytically tight and easier to use for policy discussions.

 The manuscript puts forward several important policy recommendations, including the urgent need for strengthened international governance, a moratorium on fully autonomous lethal systems, and more robust accountability mechanisms. These recommendations are relevant and well-grounded in contemporary debates. However, the causal linkages between the empirical findings and each specific recommendation are not always articulated explicitly.

 To reinforce the scientific rigor and traceability of the arguments, the conclusions section would benefit from a concise subsection that clearly maps key empirical observations to corresponding policy implications. For example:

1. Reduced levels of human oversight in Israeli systems such as Lavender and Besorah increase the risk of undermining meaningful human control;  which supports the need for technical and procedural safeguards, including minimum human review and decision times prior to target engagement.
2. Hardware limitations and system integration challenges on the Iranian side increase the likelihood of wider proliferation of low cost and lower precision AI enabled weapons; - highlighting the necessity for global normative frameworks that apply equally to both advanced and resource constrained states.

Such a structured explanation would strengthen the logical coherence of the final recommendations and ensure that the policy proposals are more directly and transparently supported by the evidence presented throughout the manuscript.

**3. Minor comments and suggestions**

1. Language and style

The manuscript is generally clear and readable, but there are scattered typographical and grammatical issues (for example "technical Architecture mappings", inconsistent hyphenation, singular or plural mismatches). A careful language edit would improve professionalism.

1. **Terminology consistency**

Terms such as "autonomous weapons", "AI supported targeting systems", "defensive autonomous weapons systems", and "LAWS" are used somewhat interchangeably. Consider defining your terminology early and using it consistently.

 Similarly, "meaningful human control" should be aligned with existing ICRC and Article 36 discussions where possible.

1. **Figures and tables**

The figures and tables (for example Iranian cyber and information operations structure, Iranian AI drone capability chart, technology capability comparison) are helpful. It would be useful to ensure that each is explicitly referenced and briefly explained in the text at the point where it first becomes relevant, with a clear statement of the data sources and whether the figure is conceptual or derived from quantitative scoring.

1. **Data availability section**

The Zenodo repository description is detailed and appropriate. You might also state explicitly which figures and tables in the article are directly generated from that dataset, to underline reproducibility.

1. **Limitations**

While limitations are touched upon (biases in conflict zone information, propaganda, limited verification), it would help to gather them into a dedicated limitations subsection, clearly distinguishing:

-
  -
    - Data access and verification limitations.
    - Methodological constraints (secondary data only, absence of primary interviews or technical access).
    - Scope limitations (focus on two states within one region).

**4. Overall assessment**

 The manuscript addresses a highly topical and important subject, combining technical, strategic, and legal perspectives on AI assisted targeting in the Iranian - Israeli context. The conceptual framework is sound, and the article makes a useful contribution, particularly by juxtaposing a technologically advanced precision orientation with an asymmetric, resource constrained approach.

 To be fully scientifically sound and maximally useful for both academic and policy audiences, the work needs clearer methodological specification, more explicit treatment of uncertainties and contested evidence, and a tighter linkage between empirical findings and legal or policy conclusions. These issues are, in my view, addressable through revisions.

Is the work clearly and accurately presented and does it cite the current literature?

Yes

If applicable, is the statistical analysis and its interpretation appropriate?

Not applicable

Are all the source data underlying the results available to ensure full reproducibility?

Yes

Is the study design appropriate and is the work technically sound?

Partly

Are the conclusions drawn adequately supported by the results?

Partly

Are sufficient details of methods and analysis provided to allow replication by others?

Partly

Reviewer Expertise:

My research focuses on the study, development, and practical implementation of military defense systems. This includes the analysis of modern defense technologies, the creation of protective architectures for armed forces, and the integration of innovative solutions aimed at enhancing operational safety and combat effectiveness. I investigate both the theoretical foundations and the applied aspects of defense systems engineering, ensuring that new concepts can be translated into real deployment and support capabilities. Particular attention is given to the improvement of resilience, adaptability, and efficiency of military systems in diverse operational environments.

I confirm that I have read this submission and believe that I have an appropriate level of expertise to confirm that it is of an acceptable scientific standard, however I have significant reservations, as outlined above.

# Response

**Published:** 2026-02-21

**Authors:** Lamia faris

**Affiliations:** computer science, University of Anbar, Ramadi, Al Anbar Governorate, Iraq

We sincerely thank the reviewers for their thoughtful comments and constructive feedback, which have helped us improve the quality of our manuscript. We carefully considered each point raised and made the following revisions
